# Metallotexaphyrins as MRI-Active Catalytic Antioxidants for Neurodegenerative Disease: A Study on Alzheimer’s Disease

**DOI:** 10.1016/j.chempr.2019.12.016

**Published:** 2020-03-12

**Authors:** James T. Brewster, Gregory D. Thiabaud, Peter Harvey, Hadiqa Zafar, James F. Reuther, Simone Dell’Acqua, Rachel M. Johnson, Harrison D. Root, Pedro Metola, Alan Jasanoff, Luigi Casella, Jonathan L. Sessler

**Affiliations:** 1 Department of Chemistry, the University of Texas at Austin, Austin, TX 78712-1224, USA; 2 Department of Biological Engineering, Massachusetts Institute of Technology, Cambridge, MA 02139, USA; 3 Sir Peter Mansfield Imaging Centre, Division of Clinical Neuroscience, School of Medicine, University of Nottingham, Nottingham NG7 2RD, UK; 4 Department of Chemistry, University of Massachusetts Lowell, Lowell, MA 01854, USA; 5 Department of Chemistry, University of Pavia, Via Taramelli 12, 27100 Pavia, Italy; 6 Accelerated Research Initiative, University of Texas at Austin, Austin, TX 78712, USA; 7 Center for Supramolecular Chemistry and Catalysis, Shanghai University, Shanghai, China

**Keywords:** neurodegeneration, metalloantioxidant, MRI, Alzheimer’s disease, amyloid beta, expanded porphyrin, texaphyrin, amyloid aggregation, *C. elegans* AD models, amyloid beta modifications by ROS and RNS

## Abstract

The complex etiology of neurodegeneration continues to stifle efforts to develop effective therapeutics. New agents elucidating key pathways causing neurodegeneration might serve to increase our understanding and potentially lead to improved treatments. Here, we demonstrate that a water-soluble manganese(II) texaphyrin (MMn) is a suitable magnetic resonance imaging (MRI) contrast agent for detecting larger amyloid beta constructs. The imaging potential of MMn was inferred on the basis of *in vitro* studies and *in vivo* detection in Alzheimer’s disease *C. elegans* models via MRI and ICP-MS. *In vitro* antioxidant- and cellular-based assays provide support for the notion that this porphyrin analog shows promise as a therapeutic agent able to mitigate the oxidative and nitrative toxic effects considered causal in neurodegeneration. The present report marks the first elaboration of an MRI-active metalloantioxidant that confers diagnostic and therapeutic benefit in Alzheimer’s disease models without conjugation of a radioisotope, targeting moiety, or therapeutic payload.

## Highlights

• Manganese texaphyrin binds to Aβ constructs (i.e., monomer, fibrils, and aggregates)
• MRI studies demonstrate the utility of metallotexaphyrins for detecting Aβ aggregates
• Antioxidant assays show potential utility of MMn for treating CNS neurodegeneration

## The Bigger Picture

Neurodegenerative disease is correlated with aberrant oxidation and nitration of small-molecule neurotransmitters, proteins, DNA, and cellular membrane components. Cellular damage enhanced by these modifications propagates disease progression, leading to death. Effective tools that enable insight into the complex etiology of CNS diseases could allow researchers to find a cure. Part of this effort is focused on developing imaging modalities that can assist in understanding these diseases. Here, we demonstrate a new theranostic strategy that shows promise in addressing the oxidative and nitrative malfunctions considered causal in disease progression. The approach detailed in this report also allows the MRI of aggregated Aβ, which could lead to improvements in disease diagnosis. The present work is thus expected to set the stage for the mechanistic-based design of theranostics, useful in understanding and treating neurodegeneration.

## Graphical Abstract

**Figure:**
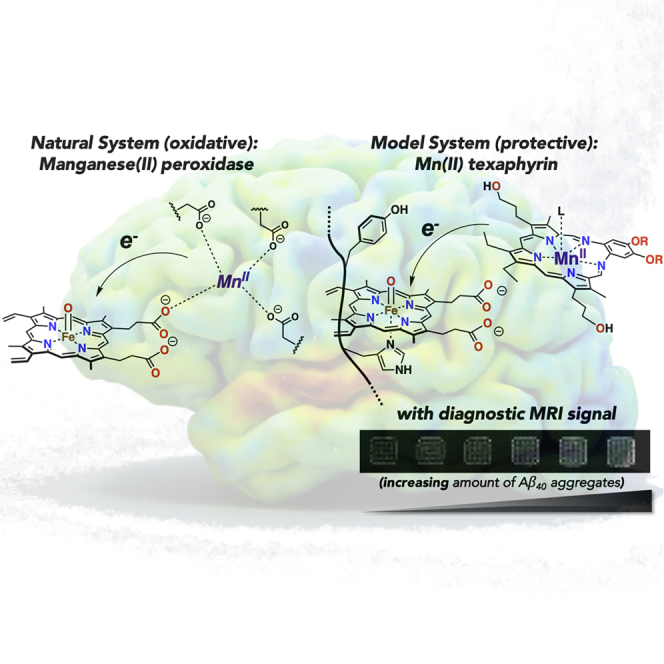

## Teaser

Sessler et al. have studied manganese(II) texaphyrin as a catalytic metalloantioxidant theranostic agent for the detection of Aβ aggregates. A series of *in vitro* and *in vivo* analyses allowed for the inference that metallotexaphyrins could be used for MRI-based detection of Aβ biomarkers. *In vitro* and cellular-based assays coupled with mechanistic analysis demonstrated an efficacious protective effect against oxidative and nitrative damage. The present results could thus guide future efforts in developing new theranostic modalities for use in neurodegenerative disease.

## Introduction

Many seminal advances in physiology and medicine have been made by hewing to a reductionism-centered mantra wherein discrete biological functions are attributed to individual processes. However, an increased understanding of the complex interactions between cellular components (e.g., DNA, RNA, proteins, and small molecules) is leading to a more nuanced view.^1^ For instance, it is now recognized that a number of key biological effects arise from interaction webs involving multiple biochemical processes operating within a scale-free network.^2^ Such an appreciation is considered particularly relevant to the problem of understanding the central nervous system (CNS) and diseases that affect it.^3^

Among CNS disorders, neurodegeneration is particularly insidious. It defines a series of complex disease states that lead to death. Current therapies, at best, ameliorate the symptoms with limited long-term benefit to patients or relief to caregivers.^4^ The complex etiology of neurodegeneration derives from known and unknown initiators operating simultaneously within a feedback loop that is thought to exacerbate downstream toxicity.^5^ In particular, Alzheimer’s disease (AD) has been postulated to arise from abnormal protein deposits, excitotoxicity, disruption of metal-ion homeostasis, reduction in endogenous antioxidants, and neuroinflammation, among other hypotheses.^6^ Most, if not all, of these putative malfunctions are correlated with an increase in reactive oxygen species (ROS) and reactive nitrogen species (RNS).^7^ Both ROS and RNS are thought to disrupt standard cellular processes giving rise to dysfunction and neuronal cell death.^8^ Here, we report that a first-generation water-soluble manganese(II) texaphyrin (MMn) displays cell permeability and allows for the detection of larger order amyloid beta (Aβ) constructs (i.e., aggregates) by means of magnetic resonance imaging (MRI). Inductively coupled plasma-mass spectrometry (ICP-MS) and MRI analyses of *in vivo* (*C. elegans*) AD models provided further support for this notion. Based on *in vitro* mechanistic studies, this texaphyrin system also mitigates the oxidative and nitrative toxicity effects considered causal in CNS neurodegeneration. It thus shows promise as a new tool that may aid in understanding, imaging, and treating neurodegenerative disease.

Deciphering the precise biochemical deviations from a normal network that are associated with CNS neurodegeneration is considered key to finding a cure.^9^, ^10^, ^11^ Within this paradigm, significant efforts have focused on developing accurate biomarkers through cerebrospinal fluid (CSF) protein analysis and positron emission tomography (PET) imaging.^12^^,^^13^ These test methods have proven useful in furthering our understanding of disease progression; however, they can be invasive and expensive. MRI has emerged as a potential complement to CSF and PET analyses.^14^ Recent efforts have also focused on the use of theranostics, wherein a fluorophore, MRI-active metal ion, or radioisotope is conjugated with a therapeutic functionality.^15^ Many of these conjugates have relied on either Pittsburgh compound B (PiB)- or curcumin-based systems for imaging combined with an Aβ aggregation inhibitor to provide therapeutic efficacy. MR-based imaging systems for the study of neurodegeneration have generally relied on Gd-DOTA or related aminopolycarboxylate ligands conjugated to antibodies or other targeting moieties. Cell and blood-brain-barrier permeability remains a major issue in such agents and could represent a serious impediment to regulatory approval and ultimate clinical utility.^16^

Expanded porphyrins, porphyrin-like systems containing a larger internal cavity than natural tetrapyrrolic congeners, have received considerable attention in recent years.^17^ One such system is texaphyrin, a penta-aza Schiff base macrocycle.^18^ Compared with porphyrins, texaphyrins contain an approximately 20% larger core and display a unique electronic structure. These systems also exhibit efficacious biological activity with cell permeability and potential in human medicine.^19^ In fact, specific water-soluble gadolinium(III) and lutetium(III) texaphyrin derivatives, known as MGd and MLu, respectively, were explored early on as potential therapeutics; the pro-oxidative MGd was studied through phase III clinical trials as a radiosensitizer, and MLu was studied through phase I clinical trials for photoangioplasty.^20^, ^21^, ^22^ Notably, MGd demonstrated a high maximum tolerated dose (22.3 mg kg^−1^) and a median half-life of 7.4 h and could be detected within brain metastases even 14 h after administration, as determined by MRI. Recently, dual-modal MGd-platinum(IV) conjugates have shown promise as potential cancer drug leads.^23^

**Figure 1:**
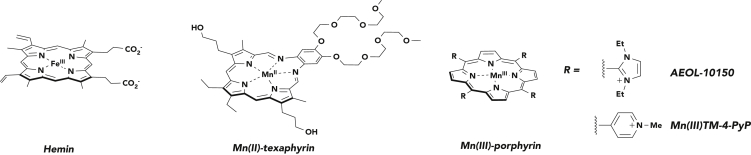
Chemical Structures of Representative Porphyrinoids As discussed in the text proper, hemin catalyzes oxidative and nitrative damage. Manganese(II) texaphyrin (MMn) is the subject of this study. Manganese(III) tetramethylpyridinium porphyrin (MnTMPyP) is a manganese porphyrin metalloantioxidant. AEOL-10150 has successfully completed a phase I trial in ALS and healthy patients and has shown efficacy in animal models for radiation- and nerve-agent-induced toxicity. Axial ligands are omitted for clarity. See main text for further discussion of these agents.

Mn(III) porphyrin and corrole metalloantioxidants have demonstrated an efficacious therapeutic effect that is thought to be mediated via decomposition of peroxynitrite (ONOO^−^) and, in some instances, superoxide and hydrogen peroxide (H_2_O_2_) (Figure 1).^24^ In particular, AEOL-10150, an octaethyl imidazolium Mn(III) porphyrin, has shown broad-spectrum superoxide dismutase (SOD)-like activity in animal models of amyotrophic lateral sclerosis (ALS), idiopathic pulmonary fibrosis, and nerve agent exposure (Figure 1).^25^ AEOL-10150 has also successfully completed a phase I clinical trial in ALS and healthy patients.^26^ Recent studies involving a water-soluble Fe(III)-corrole have also demonstrated utility in attenuating Aβ-Cu(II)-mediated ROS.^23^ However, the precise therapeutic mechanism remains recondite as Fe(III)- and Mn(III)-porphyrin and corrole complexes also catalyze oxidative transformations (e.g., peroxidase activity).^27^^,^^28^ Limited cellular uptake and acute toxicity in the case of polycationic pyridinium Mn porphyrins are also an issue.

**Figure 2:**
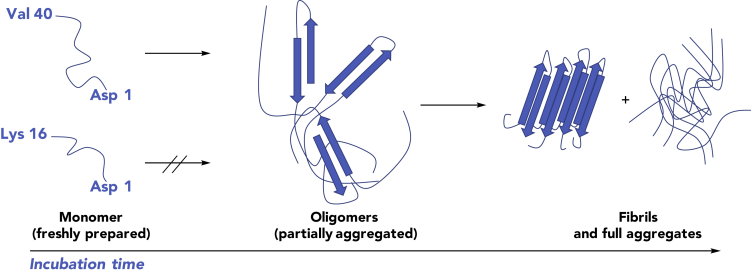
The Aggregation State of Aβ Peptides as a Function of Incubation Time The three Aβ_40_ constructs utilized in this study: (left) freshly dissolved peptide is predominantly monomeric in solution, (middle) soluble oligomeric species (partially aggregated) are produced after incubation in solution, and (right) fibrils and fully soluble and insoluble aggregates are formed after prolonged incubation. Aβ_16_ remains monomeric in solution.

The interaction of hemin and other water-soluble phthalocyanines, corroles, and porphyrins with Aβ has been noted.^29^, ^30^, ^31^, ^32^ We thus envisaged that coupling the Aβ-recognition properties of metalloporphyrins with the biological activity of metallotexaphyrins would allow for MRI recognition of Aβ species with attendant utility as a potential metalloantioxidant therapeutic (Figure 2). As detailed further below, we have found that metallotexaphyrins, in particular MMn, interact with amyloid constructs, as inferred from ultraviolet-visible (UV-vis), fluorescence, and circular dichroism (CD) spectroscopies, as well as MRI studies. The protective effect of MMn against oxidative and nitrative damage was also demonstrated by means of *in vitro* studies with Aβ peptides and serotonin as model protein and small-molecule neurotransmitter substrates, respectively, and in cell culture with Neuro-2A cells. The protective and oxidative properties of Mn(III) tetramethylpyridinium porphyrin (MnTMPyP), a gold-standard Mn porphyrin metalloantioxidant, were also studied *in vitro* and in Neuro-2A cell cultures. These studies revealed rapid decomposition of MnTMPyP under oxidative conditions consistent with a sacrificial substrate-based antioxidant mechanism. Studies involving *C. elegans* models were also carried out with MMn and provide support for the notion that this first-generation metallotexaphyrin may be administered and detected in amyloid plaque-bearing AD models. It and related systems could thus have a role to play in the study and eventual management of neurodegenerative disease.

## Results and Discussion

### Spectroscopic Analyses of Metallotexaphyrin-Aβ Interactions

**Figure 3:**
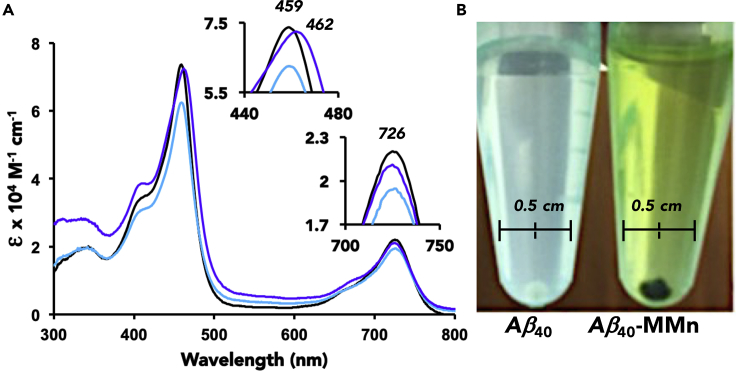
Interaction of MMn with Monomeric and Aggregated Aβ_40_ (A) Titration with MMn (5 μM) and monomeric Aβ_40_ with 0 (black), 1 (light blue), and 20 (purple) equiv and (B) photos of aggregated Aβ_40_ (100 μM; aged 2 months) in the absence and presence of MMn (100 μM) in PBS and after washing with PBS. The pellet shown in the right-most image was obtained by centrifugation and is in methanol.

Initial studies of the interactions between MMn and Aβ_40_ involved UV-vis spectroscopic analyses. This technique was chosen because changes in the intensity or wavelength (energy) of the Soret band (π-to-π∗ transition) of metalloporphyrinoids are often indicative of axial coordination.^33^ Reduction in the molar absorptivity can also arise from coordination within a peptide or protein tertiary structure.^34^ MMn, like other porphyrinoids, is intensely colored. It is dark green in most media in which it is soluble and characterized by strong Soret- and Q-like bands at λ_max_ = 459 and 726 nm, respectively, in pH 7.4 phosphate-buffered saline (PBS). The addition of monomeric Aβ_40_ (5 μM, 1 equiv) into a PBS solution of MMn (5 μM) resulted in a decrease in spectral intensity for the Soret-like band (ε = 73,700 → 62,500 M^−1^cm^−1^; λ_max_ = 459 nm) (Figure S4). Increasing the ratio of Aβ_40_ led to an enhancement in the molar absorptivity (ε = 72,450 M^−1^cm^−1^ at 20 equiv) with a slight spectral shift in the absorption maximum also being seen (λ_max_ = 462 nm; Figure 3). UV-vis spectral analyses provided support for the conclusion that the Aβ_40_ peptide (ε = 1,480 M^−1^cm^−1^ at 280 nm) remains soluble upon addition to MMn. Specifically, no sign of precipitation was observed, even when tested at high concentrations of Aβ_40_ (i.e., 100 μM; Figure S5). A titration of MMn with human serum albumin (HSA) showed a similar reduction in absorbance intensity but yielded no shift in the Soret or Q bands (Figure S6). Considered in tandem, the changes to the UV-vis spectral features of MMn observed in the presence of monomeric Aβ_40_ are consistent with the coordination of one or more axial ligands to the texaphyrin metal center. However, the potential formation of a soluble, higher-order Aβ_40_ species (e.g., a dimeric-MMn complex) in solution cannot be excluded. Under these same conditions, a titration of MnTMPyP, a commercially available porphyrin considered as a gold-standard metalloantioxidant, with Aβ_40_ yielded no change in the observed spectrum (Figure S7). Similar spectral changes to the texaphyrin profile were, however, seen when Aβ_40_ was added to MGd (Figure S7). The marked differences between MMn (and MGd) and MnTMPyP when exposed to Aβ_40_ are believed to arise from differences in the structure and charge of the overall complex. MnTMPyP is decorated with four pyridinium functionalities positioned perpendicular to the porphyrin core, whereas MMn is formally monocationic, planar, and functionalized with flexible pendant substituents capable of hydrogen bonding.

Since Aβ_16_ remains monomeric in solution and conserves the relevant amino acids considered responsible for Aβ coordination chemistry, an analogous titration was carried out between MMn and Aβ_16_ (0–80 equiv) in an effort to differentiate between the effects of aggregation (hydrophobic interactions) and axial coordination on the observed UV-vis spectral changes. UV-vis spectra of MMn recorded as it was exposed to Aβ_16_ were characterized by an initial reduction in spectral intensity, just as was observed in the corresponding Aβ_40_ studies. This decrease was followed by a corresponding increase in spectral intensity. In analogy to what was seen in the case of monomeric Aβ_40_, approximately 17.5 equiv of Aβ_16_ (as opposed to 15 equiv for Aβ_40_) were required to reach an absorbance plateau. Subsequently, no further discernible increase or change in the Soret band (λ_max_) was observed (i.e., upon addition of >20 equiv Aβ_16_; cf. Figure S8). The spectral changes induced by Aβ_16_ and monomeric Aβ_40_ were thus attributed to axial ligand binding rather than sequestration by oligomeric or aggregated peptide species.

MMn was also titrated with Aβ_40_ (0–20 equiv) that was pre-incubated for 14 days in PBS at room temperature. This pre-incubated material is believed to contain higher-order oligomeric species (i.e., partial aggregates containing β sheets; cf. Figure 2). Treating MMn with oligomeric Aβ_40_ (1 equiv) engenders a decrease in the UV-vis spectral intensity (ε = 73,700 → ε = 45,600 M^−1^cm^−1^), a finding attributed to both axial ligand binding and incorporation of MMn within the aggregate structure. Upon adding additional oligomeric Aβ_40_ (up to 20 equiv), an increase in spectral intensity (ε = 50,000 M^−1^cm^−1^) and a bathochromic shift in the Soret band maximum (to λ_max_ = 462 nm) were observed (Figure S9). Analyses of the relative spectral intensity of the Soret and Q bands (ratio of absorbance at 459 and 726 nm) revealed a minute change in this ratio for aggregated (3.39:1) and monomeric (3.43:1) forms of Aβ_40_. This was taken as further support for the suggestion that the UV-vis spectral changes seen for MMn upon exposure to Aβ_40_ originate from both axial coordination to the Mn(II) center and texaphyrin encapsulation within Aβ_40_ oligomers, fibrils, and aggregates.

To gain insights into the interactions between MMn and fully aggregated Aβ species, we mixed constructs composed of Aβ_40_ that had been aged for 2 months (a highly aggregated form) with MMn (100 μM, 1 equiv). After centrifugation, a green pellet was obtained. Even after washing and vortex mixing with deionized (DI) water (three times) and resuspension in methanol, the pellet remained green (Figure 3). A time-dependent UV-vis analysis of the spectral changes seen for MMn when exposed to this same aggregated Aβ_40_ species was carried out by following the decrease in the absorbance intensity of the Soret band (λ = 459 nm) as a function of time. Upon addition, an immediate decrease was observed. A further reduction in the absorbance intensity was then seen over the ensuing 30 min (Figure S10).

A competition experiment involving HSA and monomeric Aβ_40_ was also carried out in an effort to discriminate between selectivity for Aβ_40_ versus general biomolecule interactions. The first addition of Aβ_40_ (25 μM) to a preformed MMn (5 μM)-HSA (5.5 μM) complex induced a slight decrease in the absorption intensity of the Soret (5.8%) and Q (6.3%) bands. Subsequent addition of Aβ_40_ (25 μM) again resulted in a further decrease in the absorption intensity of the Soret (4.6%; 10.4% total) and Q (6.8%; 13.1% total) bands with concomitant bathochromic shifts in the Soret band from 459 to 460 nm and Q band from 726 to 728 nm (Figure S11). Increasing the concentration of Aβ_40_ (125 μM) and HSA (600 μM) yielded a green pellet by centrifugation in the presence and absence of HSA after the induction of Aβ fibrillization through storage at 4°C for 48 h (Figure S12).^35^ However, the MMn-Aβ_40_ pellet formed in the presence of both HSA and Aβ_40_ was smaller (ca. 0.2 cm) than that formed when MMn was treated with just Aβ_40_ (ca. 0.35 cm). No pellet was formed from MMn in the absence of Aβ_40_.

Further attempts to probe the interactions between MMn and oligomeric Aβ were made with an Aβ_40_ (aged 1 week) Thioflavin T (ThT) assay.^36^ The benzothiazole scaffold for ThT is a recognized binding agent for Aβ sequences conserved among many PET agents (e.g., the FDA-approved Vizamyl [flutemetamol (^18^F)]) and theranostics used in AD research.^37^ New small molecules with binding affinities similar to that of ThT could thus prove useful for clinical application.^30^ As important, the ThT assay can provide evidence of binding within Aβ_40_ because of the well-defined ThT-Aβ_40_ emission profile.^36^ Changes in the observed fluorescence correspond to ThT displacement or structural modification of the Aβ_40_ aggregate. To exploit this assay, we added MMn (1 to 10 equiv) in aliquots to Aβ_40_ (aged 1 week) containing ThT (ThT-Aβ_40_ constructs). These additions engendered a series of changes, including spectral shifts and an overall decrease in the intensity (Figure S13). Through-space quenching of bound or free MMn, as a result of the matched absorption spectrum of MMn and emission profile of ThT-Aβ_40_ constructs, accounts for some of the initial decrease (Figures S14 and S15). Continued modification of the fluorescence profile was seen upon the addition of each aliquot of MMn, a finding consistent with the presence of multiple binding sites within the Aβ_40_ aggregate. The observed modifications to the fluorescence profile are thus largely attributed MMn-induced structural reorganization that is triggered by binding to aggregated Aβ_40_.

We carried out high-performance liquid chromatography (HPLC) analyses in an effort to elucidate further the interactions between ThT (100 μM), MMn (100 μM), or MLu (100 μM) and 2-month-aged Aβ_40_ aggregates (100 μM) (Figure S16). After incubation at room temperature for 24 h, 77% ThT remained in the supernatant, as determined by integration against a ThT control (100 μM). Co-incubation with MMn or MLu (24 h at room temperature) effected little significant displacement given that 66.7% ThT and 7% MMn remained in the supernatant, as determined by HPLC analysis using ThT and MMn standards. A similar HPLC study using the diamagnetic MLu revealed that only 5.4% of the MLu remained in the supernatant (Figure S17). NMR spectroscopic experiments carried out with preformed ThT-Aβ_40_ samples (ThT [0.47 mM] and Aβ_40_ [0.40 mM]) in conjunction with MLu (0.49 mM) revealed signs of ThT displacement after incubation for 24 h at room temperature (Figures S18 and S19). Additional NMR spectroscopic analyses using monomeric Aβ_40_ (1.9 mM) and MLu (2.1 mM) in DMSO-*d*_*6*_ revealed changes to the proton signals ascribed to MLu and the Aβ_40_ peptide. The most notable difference after the addition of Aβ_40_ was a change in the chemical shift for the axial acetate anions originally bound to MLu; this is as would be expected under a scenario where the Aβ coordinates the metallotexaphyrin (Figure S20). In concert, these findings are taken as evidence that metallotexaphyrins bind well to aggregated Aβ, most likely through multiple binding sites, even if the specifics of the interaction are not fully elucidated.

**Figure 4:**
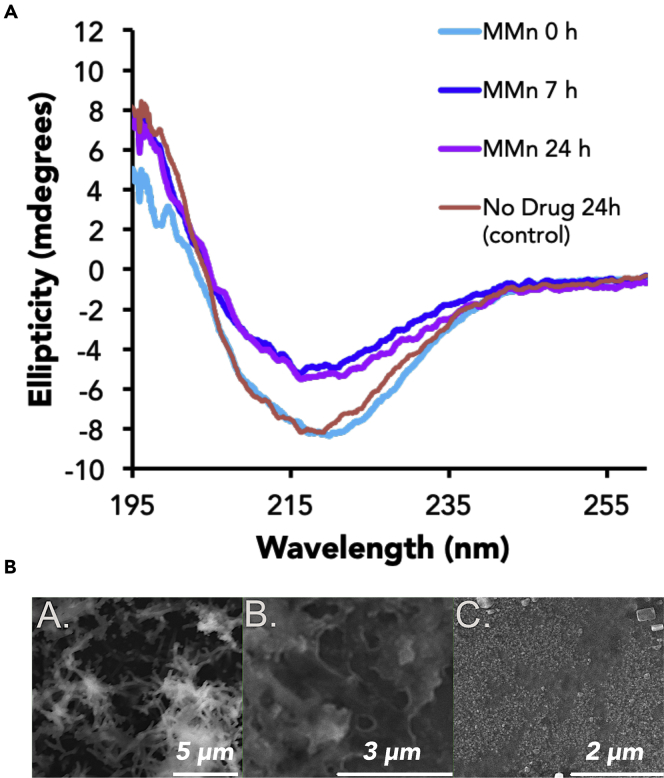
Modification of Aggregated Aβ_40_ by MMn as Monitored by CD Spectroscopy and SEM Analyses CD spectra (left) of aggregated (aged for 30 days) Aβ_40_ (40 μM) were recorded before and after the addition of MMn (20 μM) at t = 0, 7, and 24 h. SEM photographs (right) of (A) Aβ_40_ aggregate, (B) an Aβ_40_ aggregate incubated with MMn for 24 h, and (C) monomeric Aβ_40_ are shown.

Clinical trials aimed at the modification of higher-order oligomeric and aggregated Aβ have yet to yield viable therapeutics.^11^ An ability to produce and detect such modifications remains of interest in terms of assessing the potential impact of other treatment modalities. CD studies designed to assess the ability of MMn (20 μM) to interact with and modify the aggregate structure of Aβ (20 μM, aged 30 days) revealed no marked differences in the CD spectrum immediately after mixing. However, noticeable changes relative to the initial CD spectrum were observed 7 and 24 h after mixing (Figure 4). In particular, the intensity of the negative CD band at 220 nm seen for aggregated Aβ_40_ underwent a decrease in intensity of approximately 35% at 24 h with an attendant shift in the wavelength minimum to ca. 218 nm. These data are interpreted in terms of a reduction in the percentage of β sheet composition and modification of the overall aggregate structure.^38^ Control studies monitoring the progression over 24 h with aged Aβ_40_ in the absence of MMn revealed no significant change (Figure 4).

Scanning electron microscopy (SEM) provided further support for the proposed modification of the Aβ_40_ aggregate structure (Figure 4). The Aβ_40_ aggregate (40 μM) utilized for CD experiments (aged 30 days plus 24 h) was characterized by fibril structures, observed at 5 μm (Figure 4A). In contrast, the Aβ_40_ aggregate (aged 30 days) incubated with MMn (20 μM) for 24 h displayed features at 3 μm more closely resembling those of an amorphous species (Figure 4B). Freshly prepared Aβ_40_ showed no discrete features even when magnified to 2 μm (Figure 4C).

### *In Vitro* and *In Vivo* MRI

As a general rule, paramagnetic Gd(III) and high-spin Mn(II) ions enhance the relaxation rates of nearby proton nuclei. Not surprisingly, these cations have been incorporated into MRI contrast agents (CAs).^39^ Gd-based systems remain the current clinical standard. However, Mn-based CAs have recently been explored as surrogates for Gd systems and as new intramolecular MRI probes.^40^^,^^41^ Detailed MRI studies on MGd have previously been reported,^42^ including human trials where evidence of tumor-targeted imaging via enhanced drug uptake within tumor cells was obtained.^18^^,^^19^ However, the congeneric Mn(II) system MMn has yet to be the subject of extensive study in the context of MRI.^43^^,^^44^

MRI CAs are generally described by relaxivities, *r*_1_ and *r*_2_, in mM^−1^ s^−1^. These parameters are defined as the concentration-dependent enhancement of longitudinal (R_1_) and transverse (R_2_) water proton relaxation rates, each given in s^−1^, respectively. At the field used in this study (7 T), MMn displayed exceptional relaxivities in non-coordinating buffers (7 T, HEPES [pH 7.4], 25°C; MMn *r*_1_ = 4.59 ± 0.12 mM^−1^ s^−1^ and *r*_2_ = 32.2 ± 0.3 mM^−1^ s^−1^; MGd *r*_1_ = 16.0 ± 0.5 mM^−1^ s^−1^ and *r*_2_ = 23.5 ± 0.6 mM^−1^ s^−1^; Gd-DTPA *r*_1_ = 4.93 ± 0.06 mM^−1^ s^−1^ and *r*_2_ = 6.65 ± 0.02 mM^−1^ s^−1^) with well-behaved (i.e., linear) relaxation rates as a function of concentration. The longitudinal relaxivity of MMn in the coordinating buffer (i.e., PBS) was slightly reduced at *r*_1_ = 1.2 mM^−1^ s^−1^. Current CAs are known to be limited at these higher fields and previous examples of Aβ-binding MRI agents have, in general, been investigated at a lower magnetic-field strength.^39^^,^^40^^,^^45^ The ability of MMn to engender high relaxivities at a higher field could make such agents of clinical interest in the context of neurodegenerative disease. Studies with Aβ peptides were carried out as a first step toward exploring this potential.

**Figure 5:**
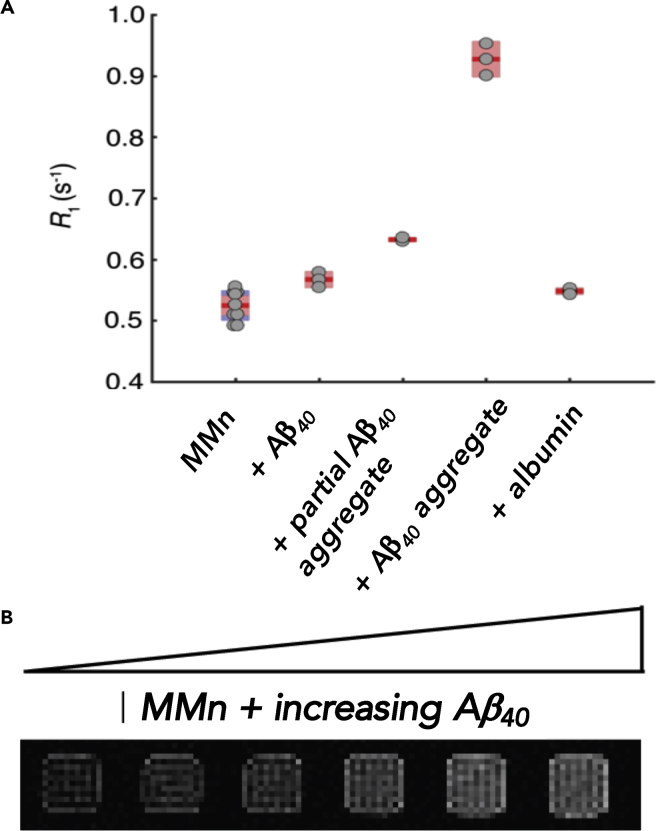
*In Vitro* MRI Analyses of MMn with Various Aβ_40_ Constructs (A) The longitudinal relaxation rate (R_1_) of MMn (140 μM) with monomeric (0.5 equiv, 70 μM), partial (1 week, 0.5 equiv, 70 μM), and aggregated (>2 months) Aβ_40_ (0.5 equiv, 70 μM) in comparison with MMn alone (140 μM) and in the presence of BSA (0.5 equiv, 70 μM). Dots represent individual samples; the mean (red line) and 95% confidence level for the mean (light red) are also displayed. Standard deviations are either indicated in blue or not shown if within the 95% confidence level. (B) Phantom T1-weighted MRI images of 140 μM MMn were recorded in the presence of increasing concentrations of Aβ_40_ aggregate (0–70 μM) in PBS at 7 T and 25°C. Samples are normalized to MMn alone, shown in the left-most box.

Changes in the relaxation rates (R_1_ and R_2_) after addition of monomeric and aggregated Aβ_40_ (70 μM) to solutions of MMn (70 or 140 μM) at 25°C in PBS (pH 7.4) were monitored at 7 T with a multi-spin multi-echo scanning sequence. Bovine serum albumin (BSA, 70 μM) was used as a control biomolecule (Figure 5). All of the additives tested gave rise to an increase in the longitudinal relaxation rates of MMn. Control studies demonstrated that Aβ_40_ alone displayed no significant effect on the relaxation rate of the buffer (R_1_ = 0.400 ± 0.005 s^−1^ versus the PBS control R_1_ = 0.398 ± 0.005 s^−1^). Relative to the relaxation value generated by MMn alone, aggregated Aβ_40_ induced the largest increase in R_1_ (Figure 5A). The modest increase observed for monomeric Aβ_40_ and albumin coupled with the observed enhancement in the R_1_ parameter in the case of aggregated Aβ is taken as a further indication that such agents could find utility in the MRI-based detection of Aβ aggregate constructs. Interestingly, with the fully aggregated Aβ_40_, the R_1_ increase was substantially larger for 2 equiv of MMn (ca. 62% versus MMn alone) than for 1:1 MMn/Aβ_40_ (ca. 37% versus MMn alone) (Figure S21). Considered in light of the ThT-Aβ_40_ fluorescence assay results described above, these data are taken as a further indication that MMn binds to multiple sites within aggregated Aβ_40_. The MRI properties of Gd(III) texaphyrin (MGd, 70 μM) in the presence of aggregated Aβ_28_ (500 μM) and BSA (600 μM) were also monitored at 7 T with a multi-spin multi-echo scanning sequence at 25°C in PBS (pH 7.4). However, these studies failed to show selectivity or significant changes in either R_1_ or R_2_ in either case (Figure S22).

Access to the intracellular intrinsically disordered proteins and peptides (IDPs) remains difficult for most MRI CAs and often comes at the cost of desirable physicochemical properties (e.g., LogP and solubility).^16^^,^^40^^,^^41^ Cell-uptake studies using MMn (30 μM), LogP 0.69, and the Neuro-2A cell line with a 9 h incubation period produced a green cell pellet with a 5.5-fold increase in the Mn content (2.2 ppb per million cells) versus the no-drug control (0.4 ppb per million cells), as quantified by ICP-MS analysis (Figure S23). MRI of the resultant cell pellet suspended in PBS provided additional support for the proposed increase in uptake given that both R_1_ (from 0.382 ± 0.004 to 0.412 ± 0.004 s^−1^) and R_2_ (from 2.43 ± 0.03 to 2.71 ± 0.03 s^−1^) were enhanced in comparison with the no-MMn control.

In order to probe the possible utility of MMn for *in vivo* MRI-based detection of neurodegenerative disease, we incubated an AD model of *C. elegans* (CL4176) expressing amyloid plaques in the presence of MMn (1 mL/100 μM).^46^ The resulting MMn content, measured by MRI and ICP-MS, was compared to what was seen in the case of the wild-type strain, N2. On the basis of reported drug absorption and retention within *C. elegans*, we envisaged that MMn would interact and be retained within the amyloid plaques, yielding higher MRI and ICP-MS signals than the control.^47^ We also used extended incubation times to allow for multiple uptake and excretion cycles. Control experiments using *E. coli* OP50 with MMn and worms without MMn were carried out concurrently with the experiments involving the N2 and CL4176 strains (Figure S24). MRI studies on the resulting pellets suspended in HEPES buffer (10 mM [pH 7.4], 1 mg pellet per 100 μL HEPES) showed an enhanced R_1_ signal in the CL4176 AD model (0.394 ± 0.012 s^−1^) when they were administered MMn versus the N2 treated with MMn in an equivalent way (0.360 ± 0.002 s^−1^), as well as no-MMn CL4176 (0.364 ± 0.003 s^−1^), N2 (0.344 ± 0.003 s^−1^), and *E. coli* with MMn control studies (0.333 ± 0.002 s^−1^). A similar trend was also seen in the case of the R_2_ with relevant values: CL4176 AD model with MMn (3.44 ± 0.20 s^−1^), N2 with MMn (2.82 ± 0.03 s^−1^), no-drug CL4176 (3.14 ± 0.02 s^−1^), no-drug N2 (2.59 ± 0.10 s^−1^), and *E. coli* with MMn control (2.450 ± 0.04 s^−1^) (Figure S25). A second batch displayed a similar trend (Figure S26).

**Figure 6:**
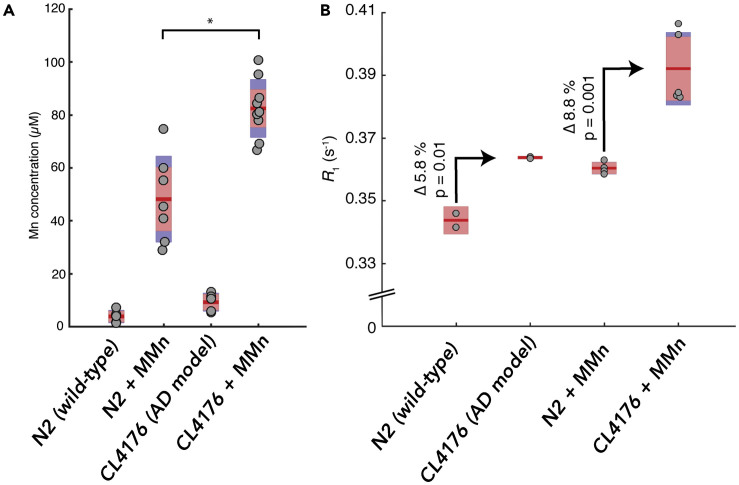
ICP-MS and *In Vivo* MRI Analyses of MMn in *C. elegans* AD Models Mn concentrations (A), as determined by ICP-MS, and (B) MRI analyses of MMn for pellets of wild-type (N2) and AD model (CL4176) *C. elegans* with and without MMn. Pellets were standardized by dissolving every 1 mg *C. elegans* in 100 μL HEPES and digesting in HNO_3_. n = 5–8 per sample, 2 replicates of each for ICP-MS measurements and n = 2–5 for MRI measurements. Dots represent individual samples; the mean (red line) and 95% confidence level for the mean (light red) are shown. Standard deviation (blue) is shown where larger than the 95% confidence level. *Two-sample t test on ICP-MS variance between MMn-wild-type (WT) and -AD models; p = 0.00018.

As expected, both batches of *C. elegans* incubated with MMn showed higher Mn signal in the N2 and CL4176 strains than did the same strain without the texaphyrin (Figure 6). ICP-MS analyses of pellet suspensions (1 mg *C. elegans* per 100 μL HEPES) revealed Mn uptake values of 82 ± 11 μM (SD) for the CL4176 AD model and 48 ± 11 μM for the wild-type N2 (Figure S27). The increased metal uptake in the AD model is taken as evidence of the accumulation of MMn within the amyloid plaque over multiple uptake-drug-excretion cycles.

### *In Vitro* ROS and RNS Reactivity Studies

Nitrative and oxidative damage is prevalent in AD brains.^48^ Oxidized and nitrated neurotransmitters, DNA bases, proteins, metalloenzymes, and cellular membrane components are also correlated with disease progression. These chemical changes are thought to alter up- and downstream biochemical processes via inactivation, hyperactivation, and off-target effects.^49^^,^^50^ Recent studies have thus led to the suggestion that oxidation and nitration of the amyloid peptide and tau proteins leads to enhanced cytotoxicity with attendant neuronal cell death.^51^^,^^52^ MMn is known to display distinct redox behavior in comparison with its related Mn(III)porphyrin and corrole congeners.^53^ Previous work on MMn has also illustrated the possibility of deactivating ONOO^−^ catalytically in the presence of sodium ascorbate (NaAsc) with attendant application in preventing formation of nitrotyrosine in ALS mice models.^54^^,^^55^ The propensity of metallotexaphyrins to interact with Aβ constructs coupled with the unique redox capabilities of MMn led us to explore whether this specific metallotexaphyrin could be used to mitigate the effects of hemin-mediated oxidation and nitration and copper-mediated oxidation of small-molecule neurotransmitters and amyloid peptides. The pro-oxidative and protective (antioxidant) activity of MnTMPyP has also previously been reported.^24^^,^^56^ It was thus studied for comparative purposes.

**Figure 7:**
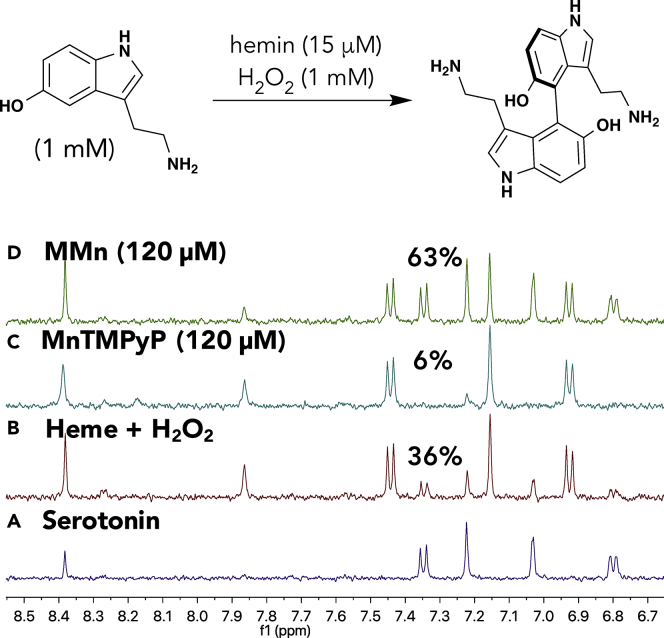
^1^H NMR Spectroscopic Study of the Hemin-Mediated Oxidation of Serotonin NMR yields were calculated by comparing the spectra of (A) serotonin (1 mM) in pH 7.4 PBS at 37°C in the presence of (B) hemin (15 μM) and an oxidative mixture containing H_2_O_2_ (1 mM) and sodium ascorbate (40 μM), (C) an H_2_O_2_-ascorbate oxidative mixture plus MnTMPyP (120 μM), and (D) an H_2_O_2_-ascorbate oxidative mixture plus MMn (120 μM) recorded after a reaction time of 120 min in all cases.

Serotonin was used as the first test substrate. When hemin is activated in the presence of hydrogen peroxide, serotonin undergoes an oxidative transformation to yield the neurotoxic 4,4′-serotonin dimer (5,5′-dihydroxy-4,4′-bitryptamine [DHBT]). The antioxidant (protective) effect of MMn was studied with serotonin (1 mM) with hemin (15 μM) and an oxidative mixture consisting of hydrogen peroxide (1 mM) and NaAsc (40 μM) in PBS (pH 7.4) at 37°C for 2 h. By comparing the changes observed in the corresponding ^1^H NMR spectra in D_2_O, we could compare the antioxidant effect of MMn against that of MnTMPyP, a well-studied Mn(III)-porphyrin metalloantioxidant, and the pro-oxidative hemin control (Figure 7). Under these conditions, MMn proved the most protective, leaving behind 63% of unmodified serotonin. In contrast, the hemin/H_2_O_2_/ascorbate and hemin/H_2_O_2_/ascorbate/MnTMPyP mixtures showed a marked increase in serotonin oxidation with 36% and 4% remaining serotonin, respectively. An analogous experiment using hemin (15 μM) and H_2_O_2_ (1 mM) without NaAsc revealed that MMn was protective, yielding 70% of unmodified serotonin versus 60% and 40% remaining for hemin/H_2_O_2_ and hemin/H_2_O_2_/MnTMPyP, respectively (Figure S28). Additional NMR spectroscopic experiments designed to evaluate the protective effect of MMn against Cu(II), another metal ion considered relevant to oxidation in CNS disorders, were also carried out (Figure S28). Qualitative ^1^H NMR spectroscopic analyses using Cu(II)Cl_2_ (100 μM), serotonin (1 mM), and H_2_O_2_ (1 mM) without and with MMn (120 μM) revealed that prolonged incubation times and high concentrations of the metal salt were necessary for producing low levels of oxidation. Shorter reaction times and lower concentrations of Cu(II) led to no detectable oxidation, as inferred from ^1^H NMR spectral studies.

**Figure 8:**
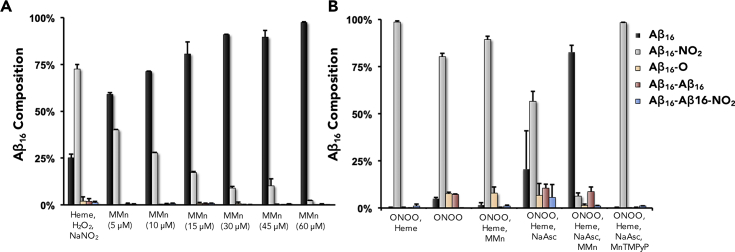
HPLC-MS Analyses of the Oxidative and Nitrative Modification of Aβ_16_ (A) Aβ_16_ incubated under hemin-nitration conditions using heme-H_2_O_2_-NaNO_2_ with varying concentrations of MMn. Percent composition of Aβ_16_ is shown in black and, as expected, increases with the [MMn]. (B) The protective effect of MMn was also tested against heme-peroxynitrite-mediated modification of Aβ_16_ in comparison with MnTMPyP. Sodium ascorbate (NaAsc) was added to regenerate the MMn catalyst under the reaction conditions. Reported values are averages with the corresponding standard deviation as obtained from two independent measurements.

MMn also proved efficient at deactivating the oxidative modification of histidine (H13-H14), nitration of tyrosine (Y10), and oxidative dimerization of tyrosine (Y10) in Aβ_16_ by the more reactive hemin, as determined by high-performance liquid chromatography-tandem mass spectrometry (HPLC-MS/MS) analysis. The Aβ_16_ sequence serves as a soluble surrogate for larger amyloid species or other proteins that could suffer degradation when treated with the oxidative mixture of hemin-hydrogen peroxide (15 μM-1 mM) or copper(II) (15, 60, or 150 μM) with hydrogen peroxide (1 mM) or nitrative mixtures of hemin-hydrogen peroxide-sodium nitrite (15 μM-1 mM-1 mM) and hemin-ONOO^−^ (15 μM-1 mM) at 37°C in phosphate buffer (pH 7.4, 5 mM). A range of reaction variables—such as the use of acidic pH thought relevant to neurodegeneration,^57^ solution ionic strength (5 versus 50 mM), reaction time (2–24 h), and the presence or absence of MnTMPyP, MGd, and curcumin—were also studied in the context of hemin-mediated oxidation and nitration of Aβ_16_ (Figures S29–S32). Within the range of a normal physiological pH of 7.4 in low-ionic-strength 5 mM phosphate buffer, only 25.1% ± 2.1% (SD) of the Aβ_16_ remained in the case of the additive-free control, composed of Aβ_16_ (300 μM), hemin (15 μM), H_2_O_2_ (1 mM), and NaNO_2_ (1 mM). MMn was found to militate against oxidation and nitration of Aβ_16_ with an IC_50_ = 4.22 μM and a maximal inhibition of 97.4% ± 0.4% at 60 μM MMn under nitrative conditions (Figure 8A). A similar protective effect by MMn was also seen under oxidative conditions of Aβ_16_ (300 μM), hemin (15 μM), H_2_O_2_ (1 mM), and MMn (30 μM), yielding 98% ± 0.4% of unchanged Aβ_16_. Control experiments without MMn, with MGd, and with the sacrificial antioxidant curcumin gave rise to 83.5% ± 5% (Δ = −13.9%), 77.5% ± 5.5% (Δ = −19.9%), and 84.5% ± 2% (Δ = −12.9%) of unchanged Aβ_16_, respectively. At a more acidic pH of 6.3 and the higher ionic strength (i.e., 50 mM phosphate buffer), under nitrative conditions, the amount of remaining Aβ_16_ (300 μM) decreased from 63.5% ± 1.4% at pH 7.4 to 33.3% at pH 6.3 (heme-H_2_O_2_-NaNO_2_; 10 μM-1 mM-1 mM). However, the protective effect of MMn (30 μM) was enhanced such that 98.8% ± 0.6% of the Aβ_16_ remained intact. This increased benefit is believed to arise from deactivation rates that are accelerated in more acidic media. The results of the Cu(II)-H_2_O_2_ experiments proved difficult to quantify. This difficulty reflects the minimal oxidative reactivity of Cu(II) at levels appropriate for HPLC-MS analysis (Figure S33). Under conditions analogous to those tested in the case of hemin, Cu(II) (15 μM) with H_2_O_2_ (1 mM) incubated at 37°C for 2 h in phosphate buffer (pH 7.4, 5 mM) returned 96.9% ± 1.7% of unchanged Aβ_16_. The addition of MMn (30 μM) to Aβ_16_ (300 μM), Cu(II) (15 μM), and H_2_O_2_ (1 mM) yielded 98.7% ± 0.4% unchanged Aβ_16_.

It is worth stressing that although it promotes the oxidation of serotonin, the gold-standard control system, MnTMPyP, was found to be protective against the oxidation and nitration of Aβ_16_. In particular, MnTMPyP showed an excellent protective effect against oxidative modification by yielding 97.4% ± 0.6% unmodified Aβ_16_. MnTMPyP also proved protective against nitrative modification by yielding 88.7% ± 0.6% unmodified Aβ_16_ (Figure S34). These are both similar to the values observed for MMn (98% ± 0.4% for oxidation and 91% ± 0.1% for nitration). The observed protective effect of MnTMPyP is thought to arise from rapid oxidative degradation of hemin and MnTMPyP via the iron- and Mn-oxo porphyrin intermediates. This loss in complex arising from MnTMPyP serving as a sacrificial antioxidant would, in turn, we propose, preclude extensive oxidation or nitration of this amyloid peptide (i.e., Aβ_16_) during the time course of the 2 h reactivity study. UV-vis analyses provided further support for the notion that MnTMPyP (5 μM) serves as a sacrificial substrate in the presence of hemin (15 μM) and H_2_O_2_ (1 mM) given that only 13% MnTMPyP and 60% hemin remain after incubation for 10 min at 37°C in phosphate buffer (pH 7.4) (Figure S35). A control experiment with hemin alone under the same conditions yielded 56% unmodified hemin.

Reactivity studies designed to test the protective effect against ONOO^−^-mediated damage of Aβ16 were also carried out. Here, a mixture of hemin (15 μM), Aβ_16_ (300 μM), and ONOO^−^ (1 mM) were incubated at 37°C for 30 min in phosphate buffer (5 mM, pH 7.4) and yielded 0.2% ± 0.1% unmodified Aβ_16_. In the presence of MMn (30 μM) with NaAsc (500 μM) as the reductant, 82.6% ± 3.7% of the original Aβ_16_ remained. Notably, in the presence of MnTMPyP and NaAsc, only 0.4% Aβ_16_ was observed intact under otherwise identical conditions (Figure 8B). We attribute this lack of protection for MnTMPyP to the rapid oxidative action of ONOO^−^ and formation of an oxidative high-valent Mn- or Fe-oxo species that could oxidize Aβ_16_ prior to decomposition.^58^ According to early mechanistic studies, such oxo species are not thought to be produced when MMn is used to deactivate ONOO^−^ in the presence of NaAsc.^54^ Control experiments with NaAsc (500 μM), MMn (30 μM) alone (not containing a reductant to regenerate the active Mn(II) species), and MGd as a texaphyrin control containing a non-redox-active metal conferred little protection, such that 20.6% ± 20.4%, 1.6% ± 1.2%, and 1.6% ± 0.2% Aβ_16_ remained intact, respectively (Figure S32).

UV-vis spectroscopic studies revealed that upon addition of MMn (1 equiv, 10 μM) to the six-coordinate 2:1 low-spin Aβ_16_-heme (200-10 μM) complex, formed by the addition of an excess of Aβ_16_ to hemin, a hypsochromic shift in the absorption of the hemin-derived features from λ_max_ = 412 to 409 nm occurred (Figure S36). Such changes can be ascribed to two possible mechanisms. First, upon addition of MMn, the reversion from 412 to 409 nm in the Soret band could reflect deconstruction of the 2:1 heme-Aβ_16_ construct to yield a mixed low- (2:1) and high-spin (1:1) Aβ_16_-heme species via MMn displacement of the heme bound to Aβ_16_.^59^ A separate mechanism could also be operable wherein MMn co-binds within the 2:1 Aβ_16_-heme construct to distrupt the Aβ axial coordination to hemin or modification of the secondary coordination environment responsible for the enhanced peroxidase activity. To such an extent that this latter rationale is correct, it would support a mechanism where MMn-mediated protection results from a proximity effect, as well as an attenuation of the heme peroxidase activity that is normally enhanced upon coordination of hemin to amyloid peptides.^59^ Both proposed mechanisms would be expected to induce changes to the UV-vis spectrum of the 2:1 hemin-Aβ_16_ complex.

**Scheme 1:**
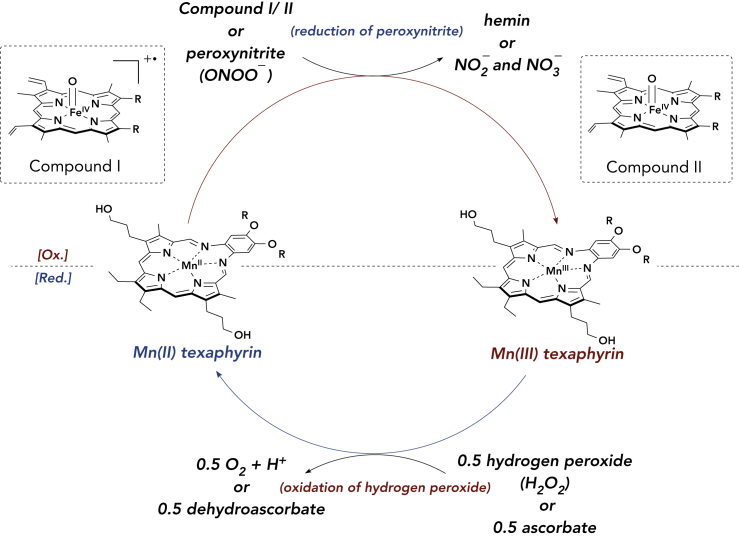
Proposed Mechanism for the MMn-Based Deactivation of Hemin- and Peroxynitrite (ONOO^−^)-Mediated Oxidation and Nitration, as well as Oxidative Decomposition of Hydrogen Peroxide (H_2_O_2_) Redox cycle of Mn(II/III) texaphyrin. Compounds I and II are generated by the reaction of hemin with H_2_O_2_. The Aβ peptide and axial ligands are omitted for clarity.

A proposed mechanism for the deactivation of iron-oxo-mediated oxidation and nitration is shown in Scheme 1. In briefl, the hemin-Aβ complex is activated by H_2_O_2_ or ONOO^−^ to yield a highly oxidative porphyrin π-radical cation/iron(IV)-oxo species (compound I in Scheme 1A).^60^ The resultant species readily oxidizes hydrogen peroxide or nitrite as well as tyrosine, histidine, or methionine residues in Aβ (producing compound II in Scheme 1). MMn could confer a protective effect by reducing compound I and/or compound II, resulting in the formation of an oxidized Mn(III) texaphyrin. In contrast to Mn peroxidase and certain model Mn complexes, such as Mn(III)TMPyP (*vide infra*), the resulting oxidized Mn(III) texaphyrin fails to carry out additional oxidative chemistry on the peptide or small redox-active molecules. The oxidized Mn(III) texaphyrin can then be reduced by ascorbate or oxidative decomposition of H_2_O_2_ to O_2_ and 2 H^+^ (Figure S37).^54^ Support for this H_2_O_2_-decomposition mechanism comes from UV-vis analyses. Complete reduction of Mn(III) texaphyrin (generated with ONOO^−^) was observed after the addition of 10 equiv H_2_O_2_ within 3 min at room temperature in PBS (Figure S38). A control experiment that involved monitoring the reduction of Mn(III) texaphyrin to Mn(II) texaphyrin without H_2_O_2_ yielded approximately 7% reduced Mn(III) after 20 min when left open to air at room temperature. Under the reaction conditions containing a large excess of H_2_O_2_ (1 mM) versus MMn (30 μM), it is expected that the H_2_O_2_ oxidation mechanism will dominate the catalytic cycle to regenerate Mn(II) texaphyrin. No oxidation of Mn(II) texaphyrin to the corresponding Mn(III) species was observed with H_2_O_2_ alone (Figure S39).

As implied above, these proposed MMn-relevant mechanisms stand in contrast to what is thought to occur with Mn(III)TMPyP. Under oxidizing conditions, such as those employed in this study, the Mn(III) center of MnTMPyP is oxidized to give a Mn(IV)-oxo species that promotes self-decomposition and substrate oxidation. The mixed protective (antioxidant) and oxidative properties of MnTMPyP is thus attributed to variations in the respective redox profile of MnTMPyP versus various biological substrates.^61^ Indeed, in instances where the substrate is more prone to oxidative modification (e.g., serotonin or methionine), a pro-oxidative-type mechanism dominates.^55^ In cases where the substrate is less labile under oxidative conditions (e.g., histidine and tyrosine) and generation of the oxidative species is slow (i.e., hemin-hydrogen peroxide), MnTMPyP instead acts as an antioxidant engendering a protective effect as a sacrificial substrate. Under more strongly oxidizing conditions, such as hemin-ONOO^−^, MnTMPyP again fails to convey any protection. The observed toxic effect for MnTMPyP in *in vitro* cell culture could also reflect the release of free Mn as a result of the rapid oxidative decomposition of this particular porphyrin complex seen upon treatment with hemin and H_2_O_2_.^61^

### Cellular Assays and Stability Studies

*In vitro* toxicity experiments with MMn using Neuro-2A revealed that MMn displays an IC_50_ of 95 μM after an 18 h incubation time at 37°C, as inferred from a standard MTT assay (Figure S40). MMn has been previously determined to be well tolerated *in vivo*, as deduced from studies involving 2.5 mg kg^−1^ daily injections in ALS G93A mice.^55^ Notably, in these same studies, HPLC analysis of Mn texaphyrin-treated mice (end of study) revealed lower levels of nitrotyrosine in the spinal cord than in untreated controls. MMn-based nanoparticles have also been evaluated *in vivo* by subjecting healthy female BABL/c mice (n = 5) to high-dose injections (10 mg kg^−1^).^42^ Again, good tolerability was seen.

*In vitro* stability studies in the presence of ascorbate, albumin, zinc, copper, and citric acid revealed good stability at 37°C over 24 h (Figure S41). In the presence of the oxidative mixture composed of hemin and H_2_O_2_, MMn displayed increased stability versus hemin and MnTMPyP when incubated at 37°C (Figure S42). MMn, MnTMPyP, and hemin all displayed moderate stability when incubated with ONOO^−^ (2 equiv) at 37°C for 0.5 h with 80%, 84%, and 71% of these complexes, respectively, after the addition of excess NaAsc (Figure S43).

**Figure 9:**
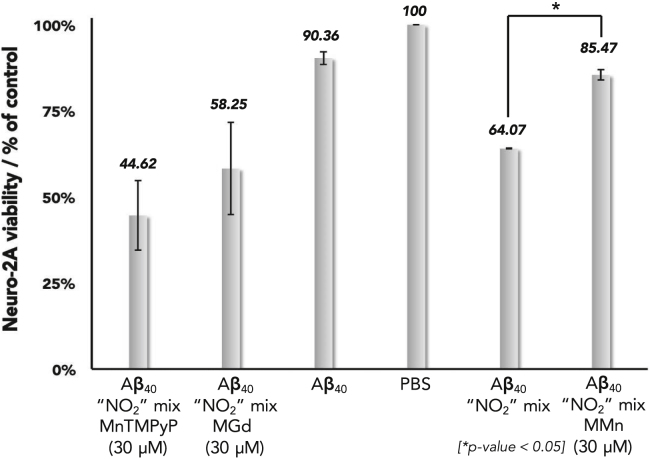
Cellular (Neuro-2A) MTT Assay Neuro-2A cell viability upon exposure to Aβ_40_ and mixed oxidized-nitrated Aβ_40_ (hemin-H_2_O_2_-NaNO_2_) with and without MMn, MGd, and MnTMPyP. Reported values are the average with standard deviation of three independent assays each tested four times. The Aβ_40_ “NO_2_” mix is an average of two independent assays each tested four times. *Two-sample t test on percent cell viability (MTT assay) between the Aβ_40_ “NO_2_” mix with and without MMn; p = 0.00016.

The cellular protective effect of MMn was demonstrated via MTT assays with Neuro-2A cells. Previous studies have shown that the cytotoxic effects of Aβ are dependent upon the aggregate structure (i.e., fibril, oligomer, and aggregate).^62^^,^^63^ Oxidative and nitrative modifications of the peptide alters its aggregation properties and can enhance cytotoxic pathways.^64^, ^65^, ^66^, ^67^ We thus sought to determine how partial oxidation and nitration of Aβ_40_, coupled with modification of the aggregated Aβ_40_ architecture, affects cell viability. The Aβ_40_ peptide was chosen since, together with Aβ_42_, it is one of the predominant Aβ species found within the AD brain. Aβ_40_ also displays a slower aggregation rate and lower toxicity than Aβ_42_, allowing for enhanced control in cell experiments. We assessed the protective effects of MMn and MnTMPyP by incubating Aβ_40_ for 3 h at 37°C in the presence of a nitration mixture containing hemin, H_2_O_2_, and NaNO_2_ in PBS. MGd was utilized as a negative control because of its ability to propagate oxidative damage.^19^^,^^23^ We then stored the reaction mixture at 4°C overnight to allow the formation of additional toxic fibrils^35^ before transferring it to wells containing Neuro-2A cells and subjecting it to incubation for an additional 24 h. In comparison to the unmodified Aβ_40_ not subjected to the nitration conditions (90.4% ± 2%), the mixed oxidized-nitrated Aβ_40_ was more toxic, resulting in only 64.1% ± 0.1% cell viability. In the presence of MMn (30 μM), the neuroblastoma Neuro-2A cell viability was increased to 85.5% ± 1.5%. MGd (30 μM) displayed cytotoxicity similar to that of the mixed oxidized-nitrated Aβ_40_ system, where only 58.25% ± 13% cell viability was observed (Figure 9). MnTMPyP (30 μM) also produced a lower level of cell viability (44.62% ± 10%). On the basis of these experiments, we conclude that MMn provides a protective effect against the oxidative and nitrative modifications that are thought to yield the neurotoxic fibril and oligomeric species that lead to cell death. In the event, the incubation with MMn translates into improved cell viability.

Qualitative HPLC-MS analyses were carried out in an effort to correlate further the level of Aβ_40_ oxidation and the observed survival effects seen in Neuro-2A (Figure S44). A mixture of Aβ_40_ (100 μM), hemin (15 μM), and H_2_O_2_ (1 mM) was incubated with or without MMn (30 μM), MnTMPyP (30 μM), or MGd (30 μM) in PBS at 37°C for 3 h. MMn conferred the greatest protective effect, yielding an approximately 1:1 ratio of oxidized (Aβ_40_-[O] to native Aβ_40_). This stands in contrast to the sample containing MGd, where the resultant HPLC trace showed a 1.7:1 ratio of Aβ_40_-[O]/Aβ_40_. Finally, MnTMPyP induced a high level of oxidative modification with an approximately 3:1 ratio of Aβ_40_-[O] to Aβ_40_ being seen. Tandem mass spectrometry (MS/MS) analysis proved consistent with oxidation occurring primarily at His13-14 and Met35. When considered in conjunction with the N2 cell data, the observed toxicity data are found to correlate with the levels of oxidative and nitrative modification in the case of the Aβ_40_ peptide. Previous studies have demonstrated that variations in cell toxicity are correlated with the Aβ_40_ aggregation state, meaning that they could be subject to potential modulation (i.e., enhanced or retarded toxicity) by porphyrinoid additives.^30^, ^31^, ^32^ MMn appears particularly effective in this regard.

### Conclusions

In conclusion, we have demonstrated that metallotexaphyrins interact with Aβ peptides and aggregates and could hold promise for the MRI-based recognition of amyloid aggregates. The first-generation water-soluble MMn that is the subject of the present study also protects against oxidative and nitrative damage of small molecules and peptides and reduces Aβ-mediated cell death. MMn was also shown to permeate the cellular membrane and be taken up within *C. elegans* animal models of AD. The present results thus serve to highlight the potential of MRI-active metalloantioxidants as potential treatment- and diagnostic-related modalities for use in the diagnosis and management of neurodegenerative disease.

## Experimental Procedures

### Synthesis of Aβ_16_ and Aβ_40_

Aβ peptides were prepared via Fmoc amino solid-phase peptide synthesis with a Liberty Blue microwave peptide synthesizer. Preparative reverse-phase high-performance liquid chromatography (RP-HPLC) purification of peptides was performed with an Agilent Zorbax SB-C_18_ Prep HT column 21.2 × 250 mm. Analytical RP-HPLC characterization of peptides was performed with an Agilent Zorbax column 4.6 × 250 mm. An Agilent Technologies 6530 Accurate Mass QT of LC/MS was used for high-resolution mass spectra of purified peptides. Solvents used were HPLC grade.

### Synthesis of MGd, MMn, and MLu

Motexafin gadolinium (gadolinium(III) texaphyrin [MGd]) was a gift from Pharmacyclics Inc., recently acquired by Abbvie. MLu and MMn were synthesized according to literature protocols.^53^^,^^68^

### Cell-Uptake Studies

Neuro-2A cells were grown until confluent in T-75 flasks. MMn was added (100 μM) and cells were incubated for 9 h, upon which cells were washed twice with PBS, incubated with trypsin (1×) for 2 min, diluted with supplemented Eagle's minimal essential medium (EMEM), counted, and pelleted by centrifugation (3 min at 2,000 rpm). After supernatant removal, the pellet was suspended and washed one more time with PBS (5 mL), and the pellet was reformed by centrifugation (3 min at 2,000 rpm). The cell pellet was digested in concentrated nitric acid (100 μL) for 15 h at 60°C and diluted with miliQ water to a final concentration of 2% HNO_3_ before being analyzed by ICP-MS.

### *C. elegans* Culture

The *C. elegans* strains N2 and CL4176 were provided by CGC (nematodes shipped on 35 mm diameter plates), which is funded by the NIH Office of Research Infrastructure Programs (P40-OD010440). *C. elegans* were grown and maintained as described in “WormBook: The Online Review of *C. elegans* Biology” by Theresa Stiernagle (https://www.ncbi.nlm.nih.gov/books/NBK19649/).

### *C. elegans* MMn Uptake

After the *C. elegans* animals, N2 and CL4176, were received from the CGC (University of Minnesota) on petri dishes, agar matrix was cut in small chunks (0.5 × 0.5 cm) with a razor blade and transferred on nematode growth medium (NGM) agar plates previously seeded with *E. coli* strain OP50 as a food source. NGM plates were then placed at 16°C in order to increase the population of *C. elegans* before drug exposure and prevent significant plaque formation in the CL4176 AD model. After 7 days, MMn was added to plates (1 mL per plate of a solution at 100 μM), which were incubated at 25°C for 48 h. The animals were then transferred to Eppendorf tubes (previously weighted empty on an analytical balance) with 1.5 mL of water. The tubes were put on ice for 5 min and centrifuged (14,000 rpm for 1 min), and the supernatant was removed. The plate was washed again with 1.5 mL of water and centrifuged again. The resulting pellets were washed twice with 1 mL of DI water. *C. elegans* pellets were lyophilized over a period of 15 h before being weighed (dry pellet weights obtained by lyophilization were used to normalize the MRI and ICP-MS results).

### Preparation of Peroxynitrite

Three separate solutions of NaNO_2_ (0.6 M), HCl (0.68 M) with H_2_O_2_ (0.72 M), and NaOH (3.6 M) were prepared and stored in the freezer at −20°C for 30 min. The solutions were transferred to an ice bath and allowed to sit for 30 min. The NaNO_2_ solution was added to a 100 mL beaker placed in an ice bath and stirred at >500 rpm. The HCl/H_2_O_2_ solution and NaOH solutions were added sequentially such that the NaOH came immediately after the HCl/H_2_O_2_ solution. These solutions must be added in sequence (i.e., one then the other) with as little time between the two additions as possible. The best results were obtained by adding the two solutions with a very small offset. The mixture turned yellow and was stirred in the ice bath for 5 min. MnO_2_ was added portion-wise until gas evolution ceased. The mixture was split into aliquots and centrifuged, and the peroxynitrite solution was decanted with a glass pipette. The concentration could be calculated with λ_max_ = 302 nm ε = 1,670 M^−1^cm^−1^. The best results were obtained by centrifugation in a cold room at 4°C. [peroxynitrite] = 72–86 mM.

### Serotonin-Hemin Reactivity Studies

Serotonin (1 mM), H_2_O_2_ (1 mM), hemin (15 μM), and sodium ascorbate (40 μM) without and with MMn (120 μM) or MnTMPyP (120 μM) were incubated in PBS (pH 7.4, 1.5 mL total volume) at 37°C for 2 h in the dark. The resulting mixture was passed through a reverse-phase tC18 SPE (Waters Sep-Pak, Waters) column containing 10 g of the C-18 substrate. The column was washed with 25 mL of 5% acetonitrile: H_2_O to separate the serotonin and 4,4′-serotonin dimer from paramagnetic species. The resulting solution was concentrated with a rotary evaporator. The mixture was taken up in D_2_O (0.7 mL) and analyzed via ^1^H NMR spectroscopy. NMR spectroscopic yields were calculated by comparing the relative concentration of serotonin to the 4,4′-serotonin dimer. Note that additional washing of the column failed to yield additional serotonin or the 4,4′-serotonin dimer.

### Aβ_16_ and Aβ_40_ Reactivity Studies

#### Hemin Oxidative Reactivity Conditions

Hemin (15 μM) and H_2_O_2_ (1 mM) and Aβ_16_ (300 μM) or Aβ_40_ (100 and 200 μM) in PBS (200 μL total volume) were incubated together at 37°C. The resulting mixtures were immediately subjected to HPLC-MS analysis. Vials were cooled to 4°C inside the HPLC-MS queue to prevent additional reactivity.

#### Hemin Nitrative Reactivity Conditions

Hemin (15 μM), NaNO_2_ (1 mM), H_2_O_2_ (1 mM), and Aβ_16_ (300 μM) or Aβ_40_ (100 and 200 μM) in PBS (200 μL total volume) were incubated together at 37°C. The resulting mixtures were immediately subjected to HPLC-MS analysis. Vials were cooled to 4°C inside the HPLC-MS queue to prevent additional reactivity.

#### Hemin-Peroxynitrite Nitrative Reactivity Conditions

Hemin (15 μM), peroxynitrite (1 mM), and Aβ_16_ (300 μM) in PBS (200 μL total volume) were incubated together at 37°C. All compounds were added and then vortexed to provide a homogenous mixture. Immediately after peroxynitrite was added, the mixture was vortexed again and allowed to incubate at 37°C for 30 min. The resulting mixtures were immediately subjected to HPLC-MS analysis. Vials were cooled to 4°C inside the HPLC-MS queue to prevent additional reactivity.

### Cell Culture

The neuroblastoma cell line Neuro-2A was bought from ATCC. Cells were grown in EMEM containing 10% heat-inactivated fetal bovine serum (FBS) and antibiotics (100 mg/mL streptomycin and 100 U/mL penicillin) at 37°C under 5% CO_2_ atmosphere. The cells were sub-cultured once every 4 to 5 days.

### Neuro-2A MTT Assay

Aβ_40_ (90 μM) was incubated in PBS alone or in the presence of H_2_O_2_ (1 mM), hemin (15 μM), and NaNO_2_ (1 mM) (referred to as nitration mixture) for 3 h at 37°C followed by 24 h at 4°C. 250 μL of each of the solutions was transferred into each well containing about 50,000 Neuro-2A cells (plated 24 h earlier) in 250 μL of supplemented EMEM without phenol red. After 24 h, the medium was removed and replaced with 500 μL of fresh EMEM not containing FBS or phenol red. To this was added 200 μL of an MTT solution at 3 mg/mL in EMEM not containing phenol red or FBS. After 4 h at 37°C, the supernatant was removed and 200 μL of DMSO was added to each well to dissolve the formazan purple crystals. Aliquots (50 μL) of each well were transferred into a 96-well plate well, and the absorbance at 570 nm was read with a plate reader (see description above).

## Supplemental Information

Document S1. Supplemental Experimental Procedures and Figures S1–S44

Document S2. Article plus Supplemental Information
