# Supplementary material for: Metallotexaphyrins as MRI-Active Catalytic Antioxidants for Neurodegenerative Disease: A Study on Alzheimer’s Disease
Source: Chem. 2020 Mar 12;6(3):703–24. doi: 10.1016/j.chempr.2019.12.016 (PMC7074011; doi:10.1016/j.chempr.2019.12.016)
Supplement: Document S1. Supplemental Experimental Procedures and Figures S1–S44 [file mmc1.pdf]

Chem, Volume *6*

Supplemental Information

Metallotexaphyrins as MRI-Active Catalytic Antioxidants for Neurodegenerative Disease: A Study on Alzheimer's Disease

James T. Brewster II, Gregory D. Thiabaud, Peter Harvey, Hadiqa Zafar, James F. Reuther, Simone Dell'Acqua, Rachel M. Johnson, Harrison D. Root, Pedro Metola, Alan Jasanoff, Luigi Casella, and Jonathan L. Sessler

Supplemental Data Items

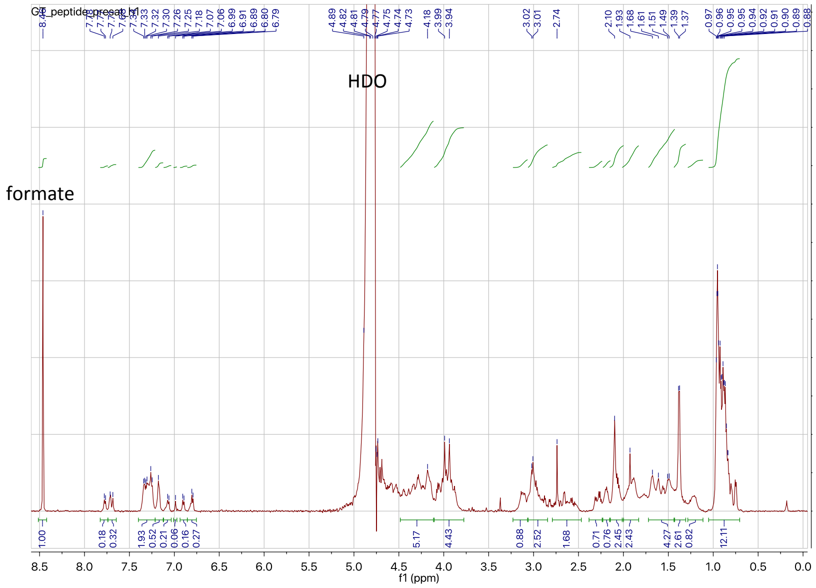

Figure S1: ^{1}H NMR spectrum of Aβ_{40} peptide (10% D_2O in PBS, 600 MHz, 298 K)

Target Compound Screening Report

Results Acquired by The University of Texas at Austin Mass Spectrometry Facility

**Data File**	MSF18-1297(GT923)_hrESIposcolumn1.d	**Sample Name**	1297(GT923)	**Comment**	1297(GT923)
**Position**	P1-B6	**Instrument Name**	Instrument 1	**User Name**	
**Acq Method**	New_pos_column_12min.m	**Acquired Time**	9/19/2018 12:30:45 PM	**DA Method**	KS.m

MS Zoomed Spectrum

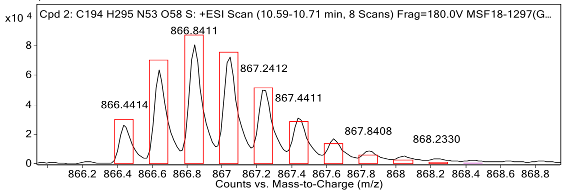

MS Spectrum Peak List

Obs. m/z	Calc. m/z	Charge	Abundance	Formula	Ion Species	Tgt Mass Error (ppm)
866.4414	866.4370	5	27333	C194H295N53O58S	(M+5H)+5	-5.17
866.6411	866.6375	5	64013	C194H295N53O58S	(M+5H)+5	-4.07
866.8411	866.8381	5	81273	C194H295N53O58S	(M+5H)+5	-3.51
867.0408	867.0386	5	73297	C194H295N53O58S	(M+5H)+5	-2.53
867.2412	867.2390	5	52042	C194H295N53O58S	(M+5H)+5	-2.45
867.4411	867.4395	5	31967	C194H295N53O58S	(M+5H)+5	-1.87
867.6407	867.6400	5	17455	C194H295N53O58S	(M+5H)+5	-0.9
867.8408	867.8404	5	9429	C194H295N53O58S	(M+5H)+5	-0.47
868.0397	868.0409	5	5678	C194H295N53O58S	(M+5H)+5	1.37
868.2330	868.2413	5	3975	C194H295N53O58S	(M+5H)+5	9.54

--- End Of Report ---

Agilent Technologies | Page 1 of 1 | Printed at: 1:00 PM on:9/19/2018

Figure S2: High-resolution mass spectrum (ESI) of Aβ_{40} peptide as $[M+5H^+]^{5+}$ species

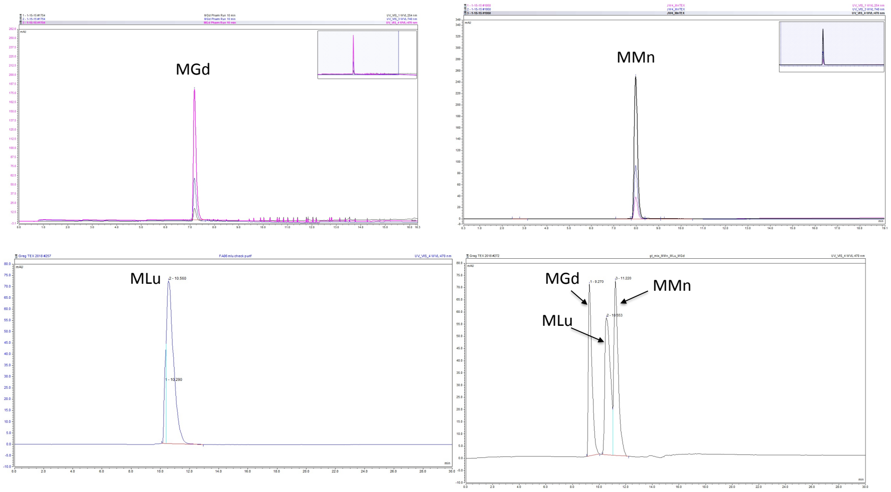

Figure S3: RP-HPLC chromatograms (detector set at 254, 470, and 740 nm) of MGd (top left), MLu (bottom left), MMn (top right), and co-elution of the three metallotexaphyrins (bottom right).

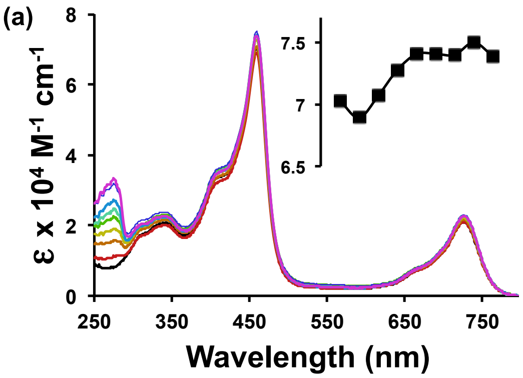

Figure S4. UV-vis spectra of MMn (5 μM) recorded upon the addition of Aβ_{40} (0-20 equiv.; black to pink) in PBS (pH 7.15) at 23 °C and insert of the molar absorptivity at $\lambda = 459$ nm (0-20 equiv.; in 2.5 equiv. aliquots). *An Aβ_{40} stock solution was prepared, the appropriate equivalents were added to a 1 mL Eppendorf tube, lyophilized, and re-dissolved in 1 mL of an MMn (5 μM) solution. The calculated value for the 17.5 equivalent data point is approximately 19 equivalents and was attributed to the method of preparation (i.e., transfer and lyophilize).*

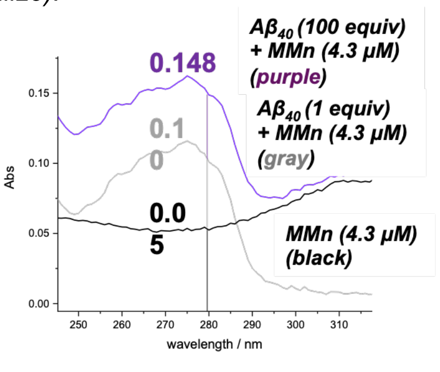

$Abs_{MMn} + Abs_{Ab40} \approx Abs_{Ab40+MMn}$

⇔ almost no precipitation of Aβ_{40}

Figure S5. UV-vis spectra of MMn (4.3 μM; *black*), Aβ_{40} (67 μM; *gray*), and MMn plus Aβ_{40} (*purple*) in PBS (pH 7.15) at 23 °C. Note: The molar absorptivity of the Aβ_{40} peptide (ε) is 1,480 $M^{-1}cm^{-1}$ at 280 nm.

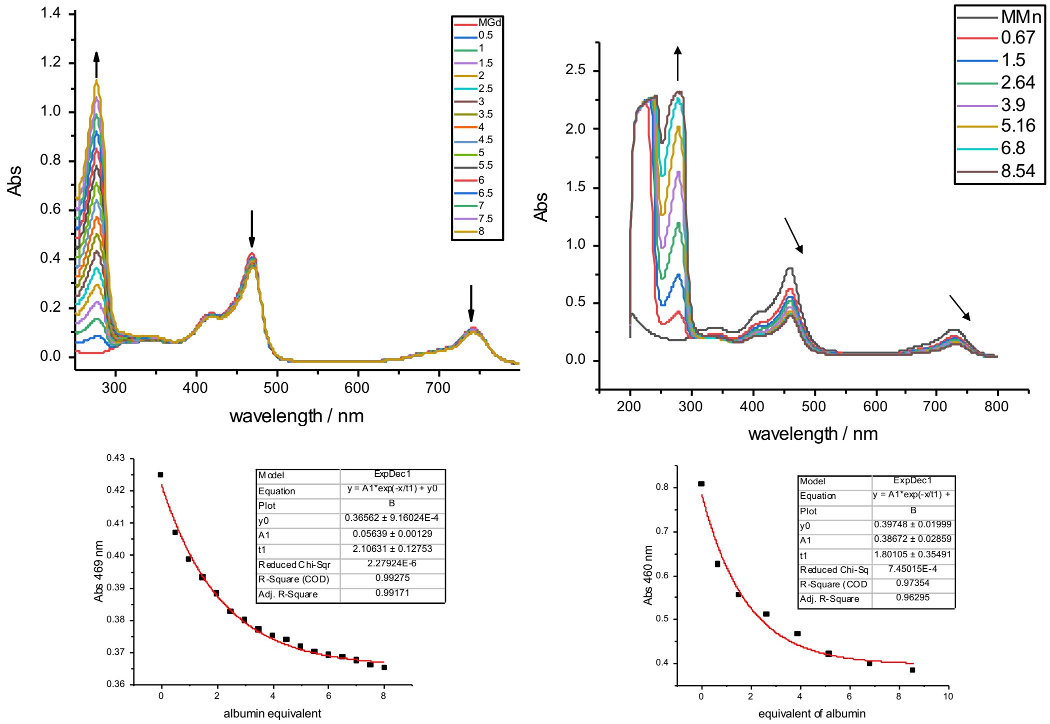

Figure S6. UV-vis (PBS, 300 K) spectral titrations of (left) MGd (5.6 μM) and (right) MMn (11.8 μM) with human serum albumin (HSA). Under each titration is plotted the evolution of the λ_{max} (Soret band) intensity against the number of equivalents of albumin added.

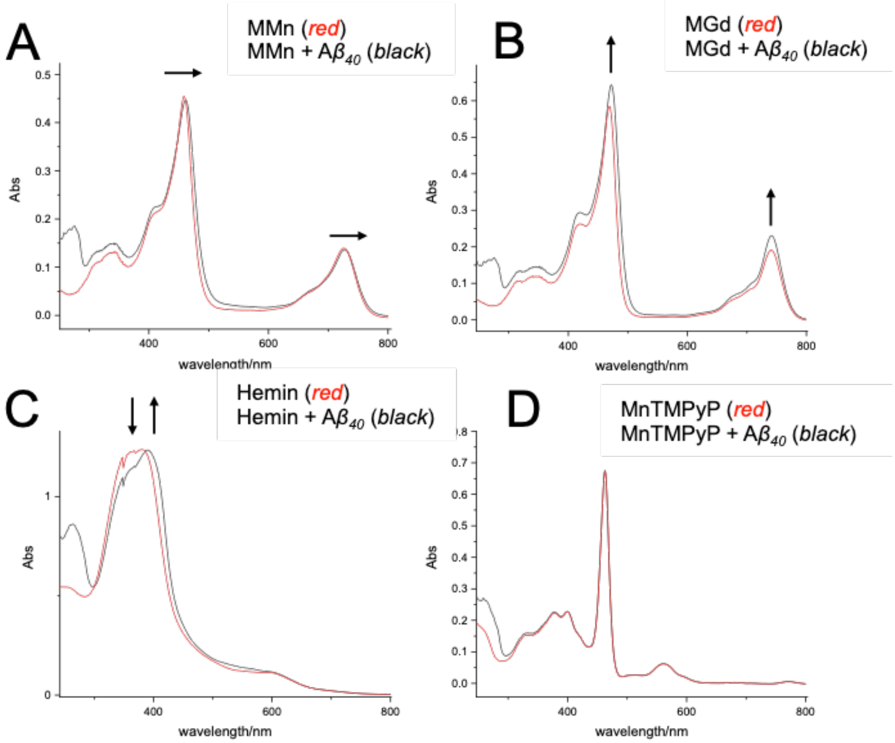

Figure S7. UV-vis spectra (300 K, PBS) of (a) MMn (7 μM), (b) MGd (7 μM), (c) hemin (14μM), and (d) Mn^{III}TM-4-PyP (7 μM) before (red) and after (black) addition of monomeric $A\beta_{40}$ (10 equiv.).

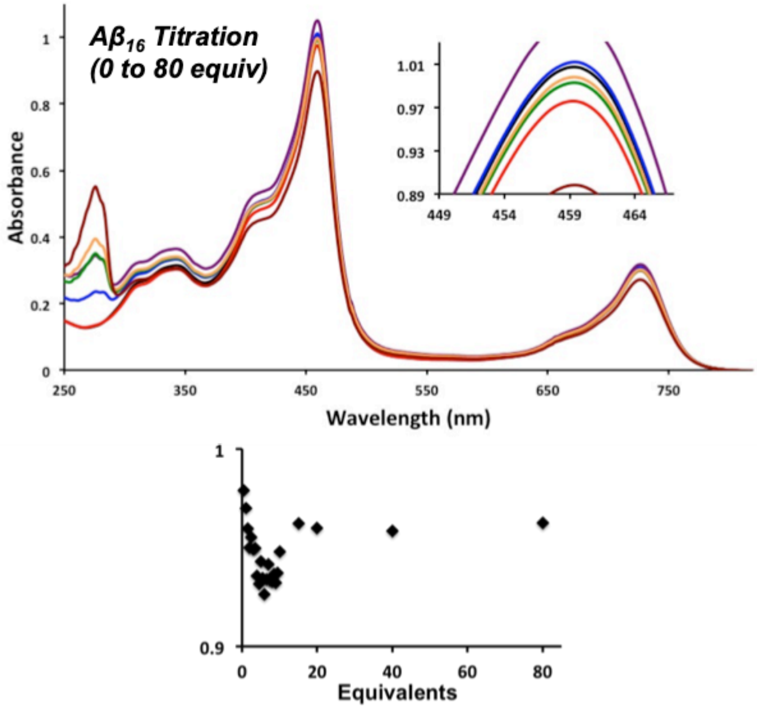

Figure S8. UV-vis spectra of MMn (5 μM) recorded upon the addition of $A\beta_{16}$ [0-80 equiv.; *black* (0 equiv.), *red* (1 equiv.), *blue* (10 equiv.), *purple* (20 equiv.), *green* (30 equiv.), *yellow* (40 equiv.), and *brown-red* (80 equiv.)] in PBS (pH 7.15) at 23 °C and insert of the molar absorptivity focused on $\lambda = 459$ nm (0-80 equiv.). Also shown is a plot of the absorbance at $\lambda = 459$ nm as a function of added $A\beta_{16}$ (0-80 equiv.).

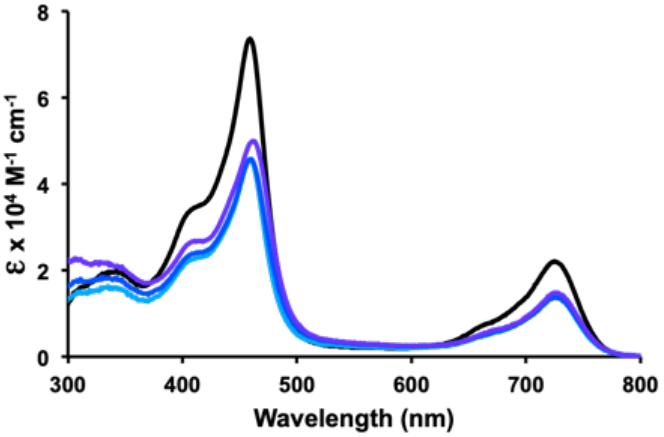

Figure S9. UV-vis spectra of MMn (5 μM) recorded upon the addition of oligomeric $A\beta_{40}$; 0 (black), 1 (light blue), 5 (blue), and 20 equiv. (purple) in PBS (pH 7.15) at 23 °C.

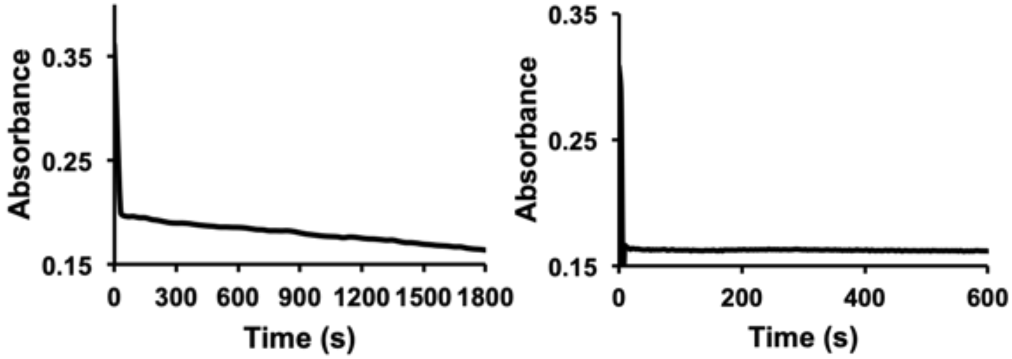

Figure S10. (left) Inferred binding of MMn (5 μM) to 2-month aged Aβ_{40} aggregates (100 μM) as determined by monitoring the changes in UV-vis spectral intensity (Abs at 460 nm in PBS at 300 K). Also shown (right) is the corresponding trace for MMn alone, which is taken as evidence that the change seen in the left is not due to photobleaching.

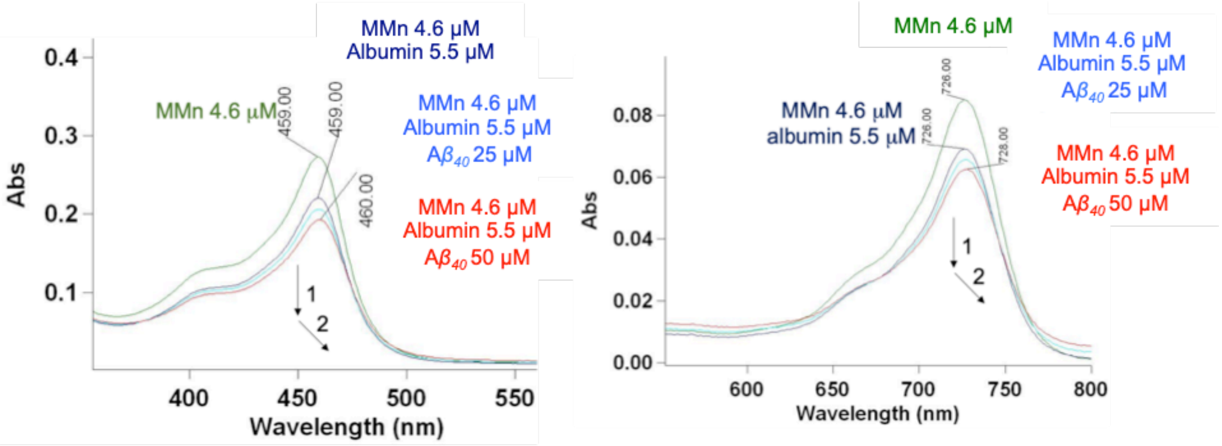

Figure S11. UV-vis spectra of MMn (4.6 μM) recorded in the presence and absence of albumin (5.5 μM) and with and without Aβ_{40} (25 and 50 μM). (left) Soret and (right) Q-band.

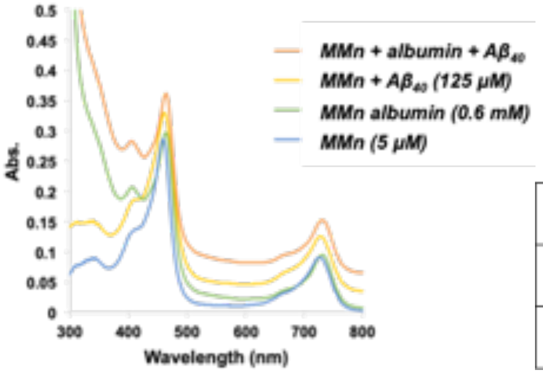

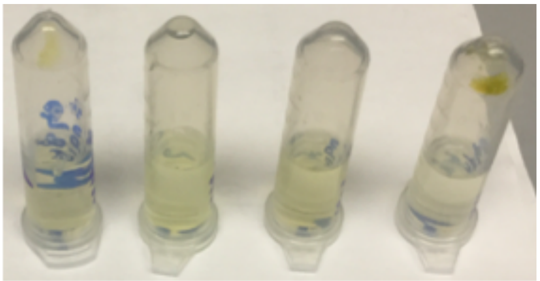

MMn 5 μM	+	+	+	+
Albumin 0.6 mM	+		+	
Aβ40 125 μM	+			+

Figure S12. (left) UV-vis spectra (PBS, 300 K) of MMn (5 μM) recorded in the absence and in the presence of albumin (0.6 mM) and with and without Aβ_{40} (125 μM) added in solid form. (right) The picture displays the same solutions after storing at 4 °C for 48 h. Pellets were obtained by centrifugation at 14000 rpm for 5 min. ***Note***: *the baseline shows the absorbance seen upon the addition of amyloid peptide and is thought to reflect its partial insolubility*.

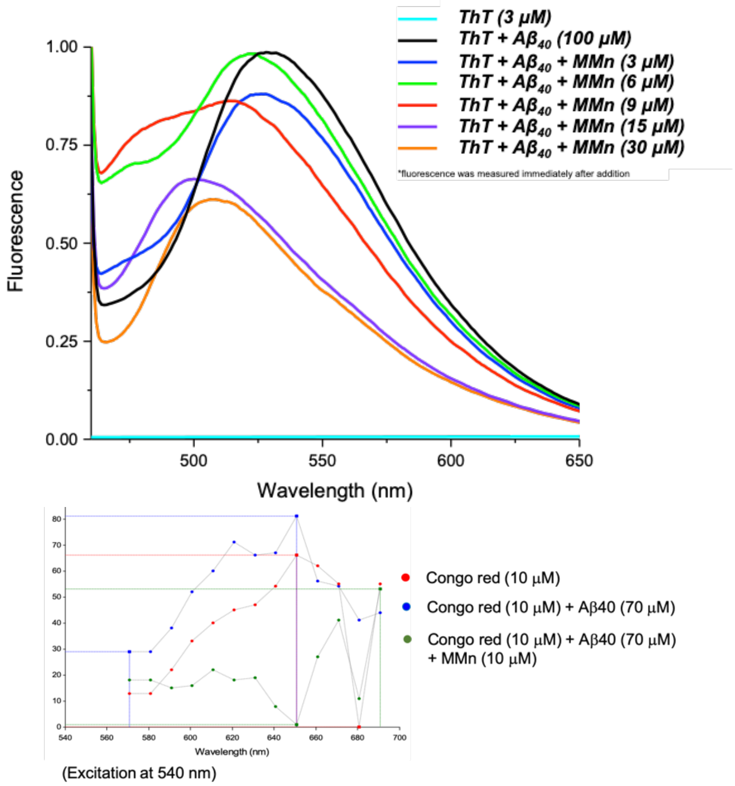

Figure S13. (top) Thioflavin T (ThT) fluorescence displacement assay using ThT (3 μM) with 1-week aged oligomeric Aβ_{40} (100 μM) and changes seen upon the addition of MMn (0 to 10 equiv.). (bottom) Congo Red (CR) fluorescence displacement assay using CR (10 μM) with 1-month aged aggregated Aβ_{40} (70 μM) and changes seen upon the addition of MMn (10 μM).

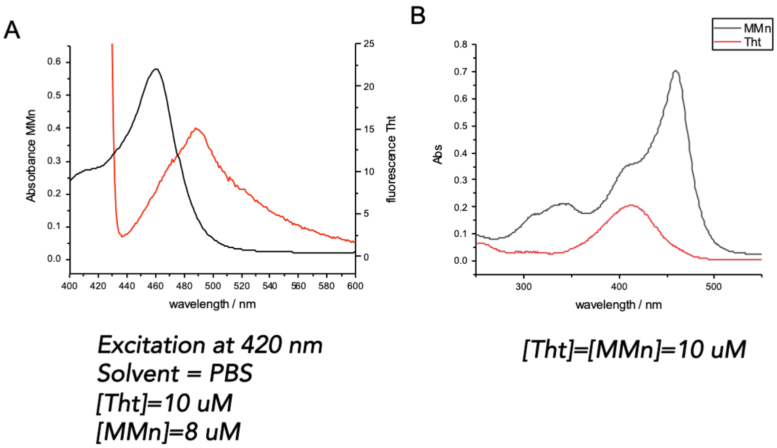

Figure S14. (a) Overlaid fluorescence and (b) UV-vis spectra of MMn (*black*) and ThT (*red*).

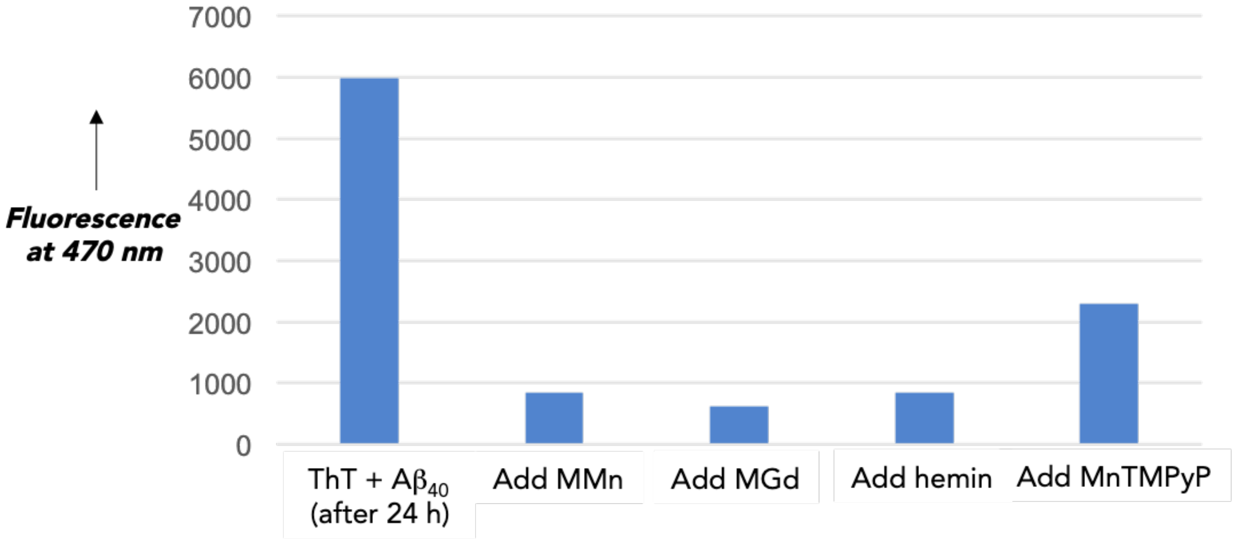

Figure S15. Fluorescence intensity values for ThT-Aβ_{40} alone and with MMn, MGd, hemin, or MnTMPyP.

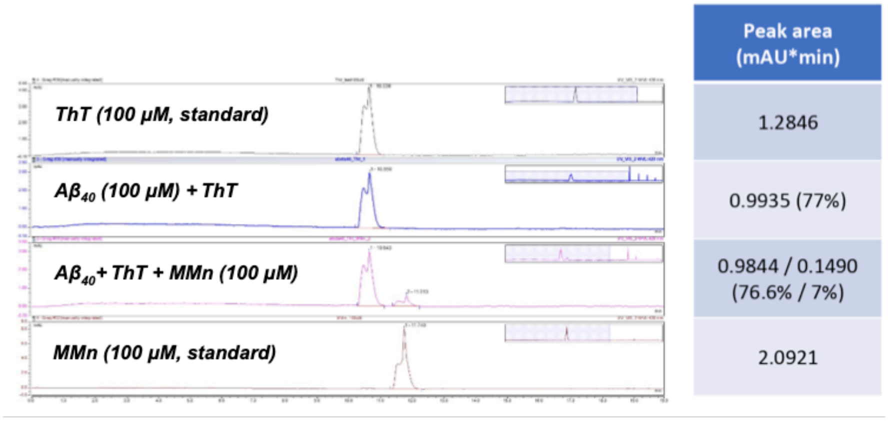

Figure S16. HPLC-MS analysis of ThT (100 μM) and MMn (100 μM) with 2-month aged Aβ_{40} (100 μM). Samples were incubated in PBS at 37 °C for 24 h then centrifuged before the supernatant was analyzed by HPLC. The area under the curve for ThT was measured at 420 nm while a wavelength of 470 nm used for MMn.

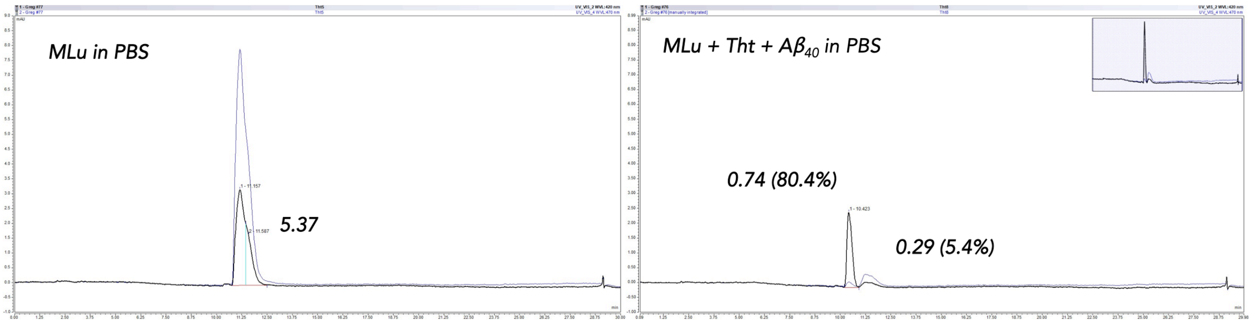

Figure S17. HPLC-MS analysis of ThT (100 μM) and MLu (100 μM) uptake within 2-month aged Aβ_{40} (67 μM). Samples were incubated in PBS at 37 °C for 40 h then centrifuged before the supernatant was analyzed by HPLC. The area under the curve for ThT was measured at 420 nm while a wavelength of 470 nm used for MMn.

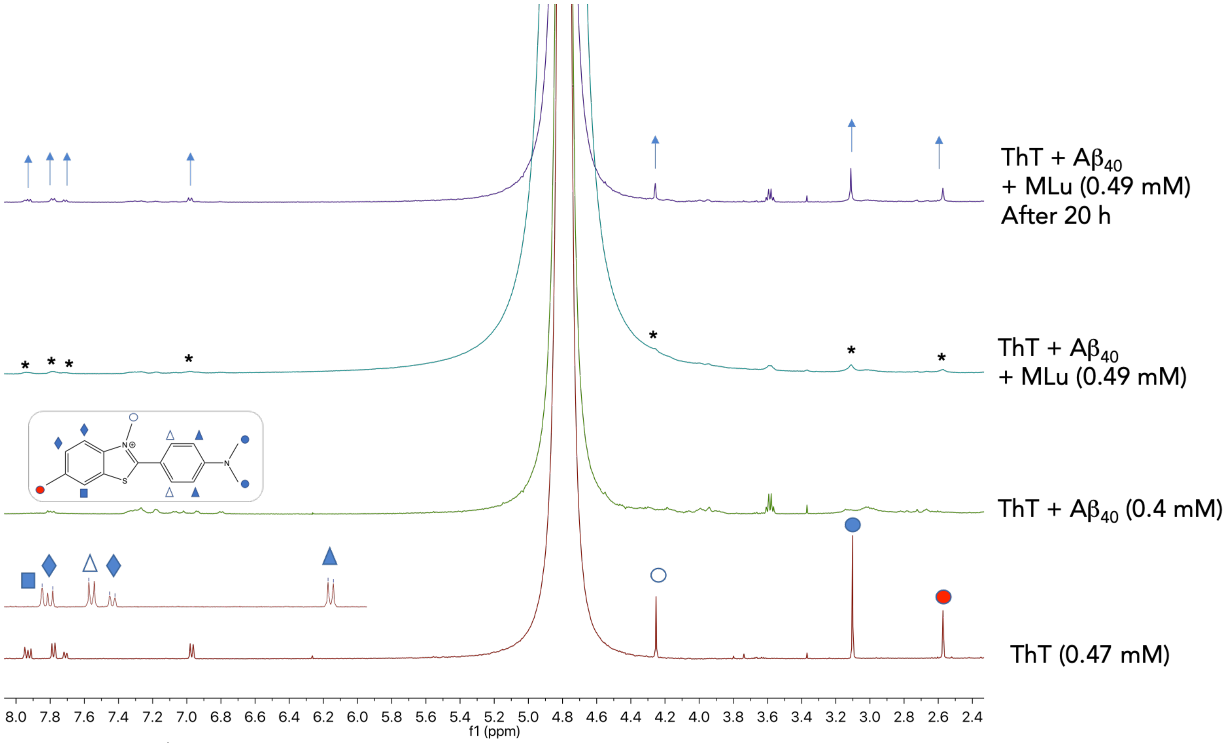

Figure S18. ^{1}H NMR spectra (500 MHz, 300 K, D_2O) of ThT with $A\beta_{40}$ and with $A\beta_{40}$ and MLu. The spectrum of the latter sample was recorded immediately after addition and after a 20 h incubation period at room temperature.

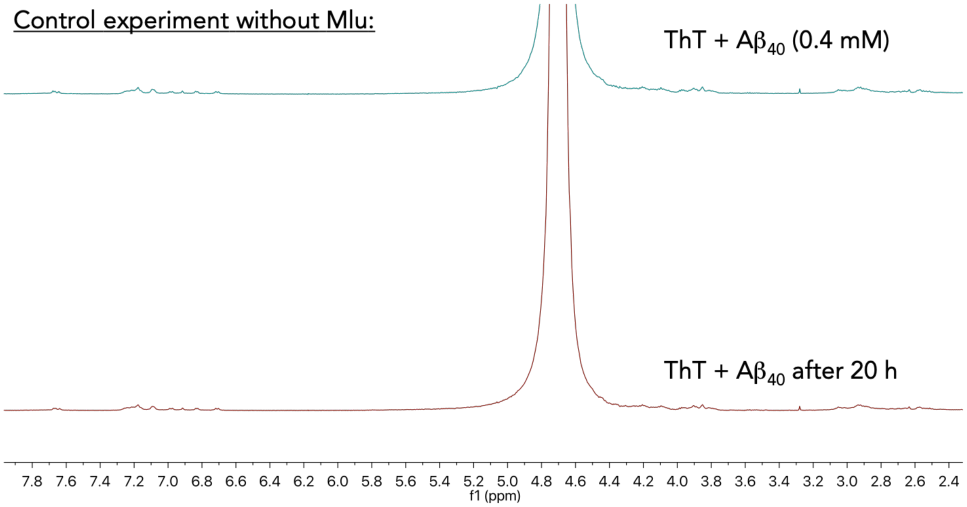

Figure S19. Control experiment showing ThT is not displaced when incubated with $A\beta_{40}$ under the same conditions.

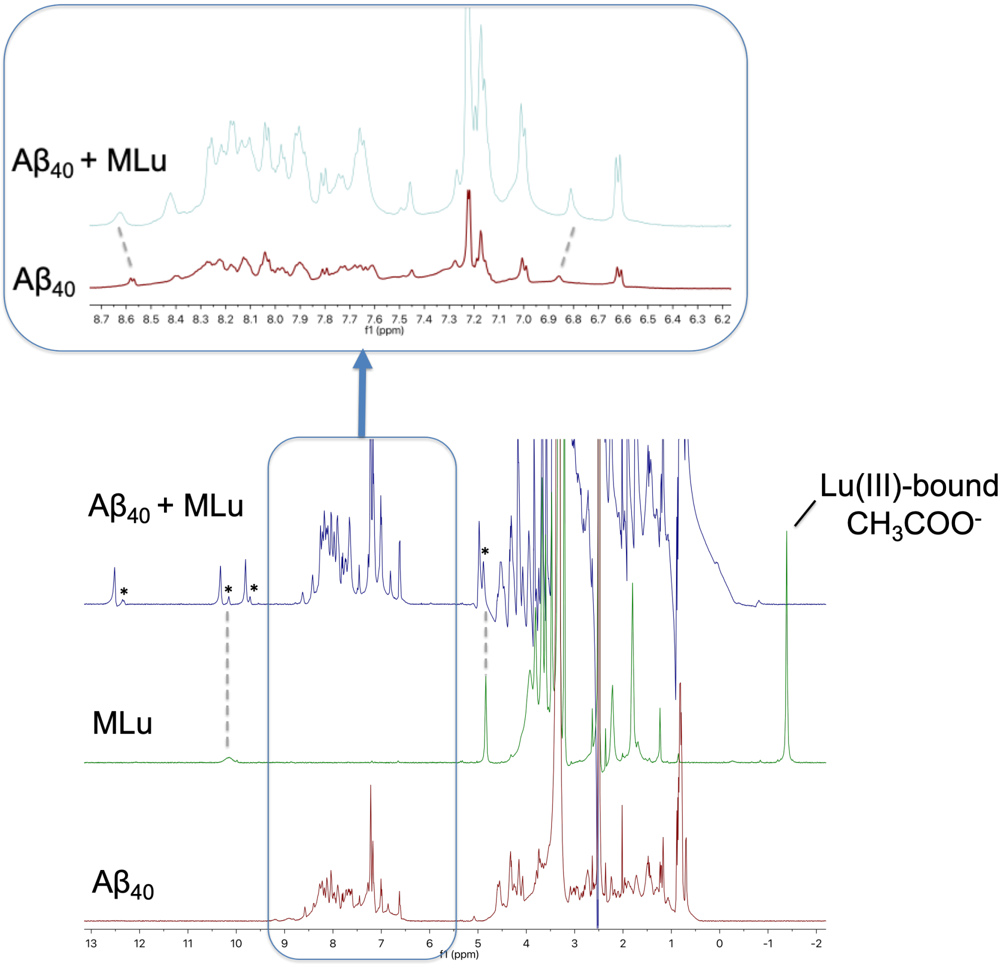

Figure S20. ^{1}H NMR spectra (500 MHz, 300 K, DMSO-d_6) of Aβ_{40} (0.66 mM, freshly prepared), MLu (2.78 mM), and a mixture of Aβ_{40} with MLu ([Aβ_{40}] = 1.9 mM, [MLu] = 2.1 mM). *Upon addition of the Aβ_{40} peptide, the acetate peak was observed to shift from its original position at -1.5 ppm.*

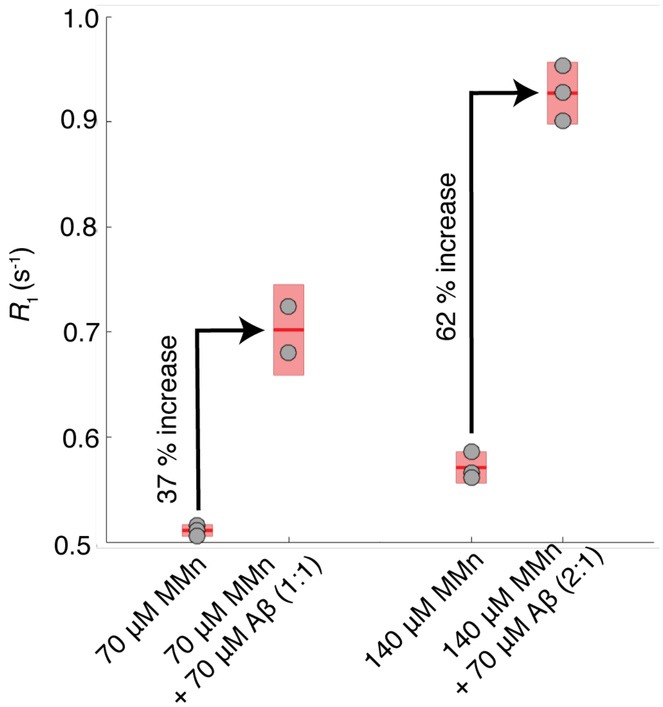

Figure S21. Variation in R_1 seen upon the addition of one and two equiv. of MMn to fully aggregated Aβ$_{40}$ (aged >2 months).

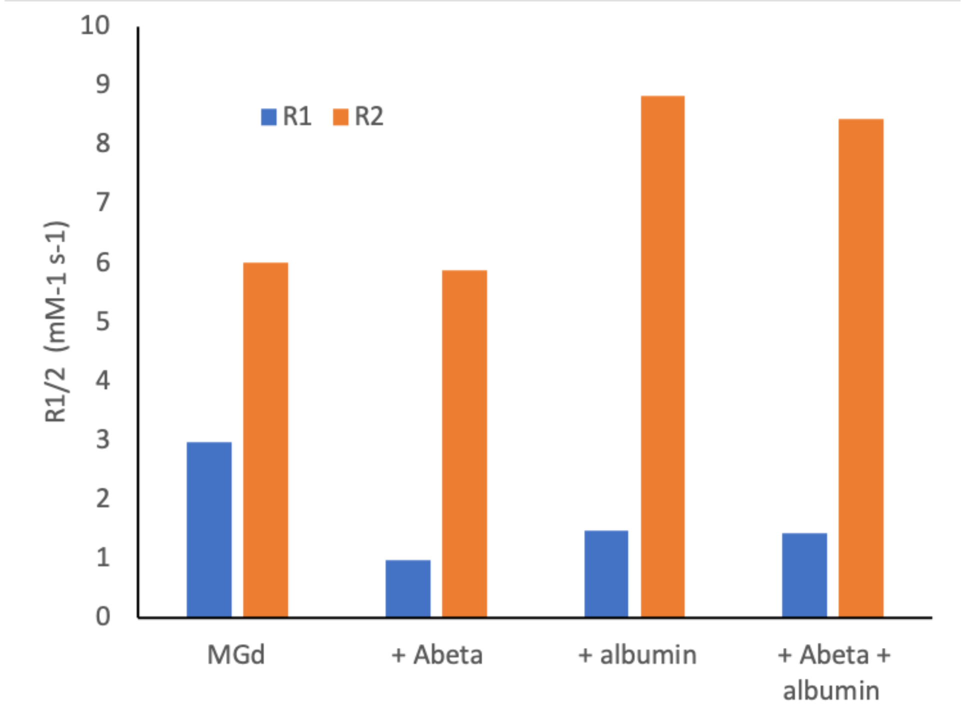

Figure S22. MRI (R_1 & R_2) analyses of MGd alone and in the presence of aggregated Aβ$_{28}$ (500 μM), BSA (600 μM), and a combination of Aβ$_{28}$ and BSA.

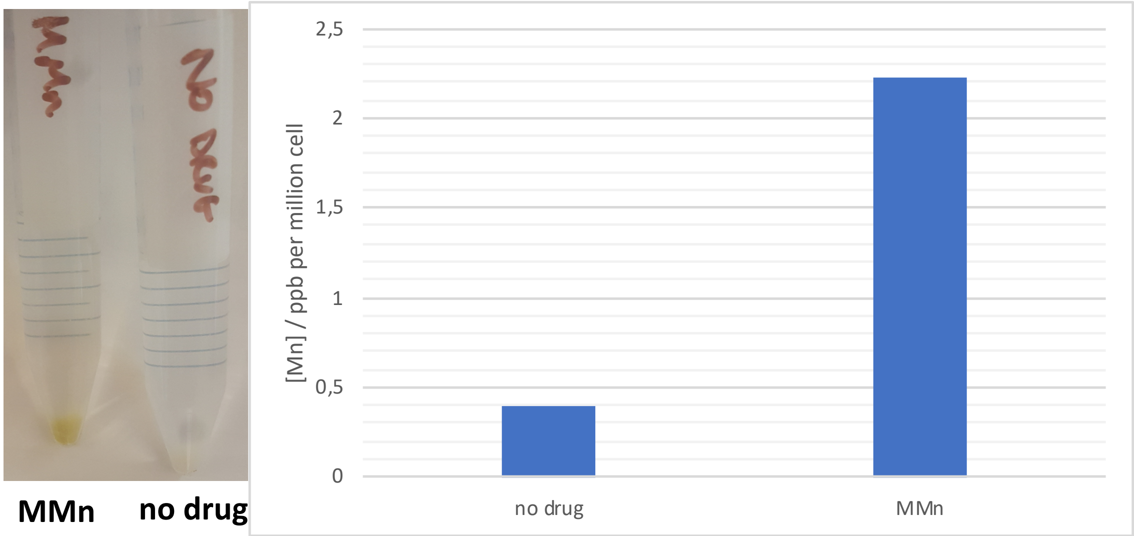

Figure S23. (left) Neuro-2A cell pellet images and (right) Mn concentration of the MMn ("drug") and no drug cell pellets measured by ICP-MS. Values are in ppb per million cells. Neuro-2A cells were grown in 5% CO_2 at 37 °C in EMEM supplemented with 10% fetal bovine serum (Invitrogen-Gibco) for 9 hours. MMn was dosed at 30 μM.

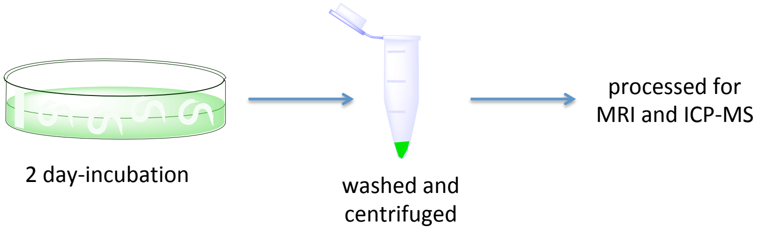

Figure S24. Preparation of *C. elegans* for MRI and ICP-MS analysis. *Pellets weighing over 2.5 mg were discarded as these samples were more than one standard deviation from the mean pellet weight.*

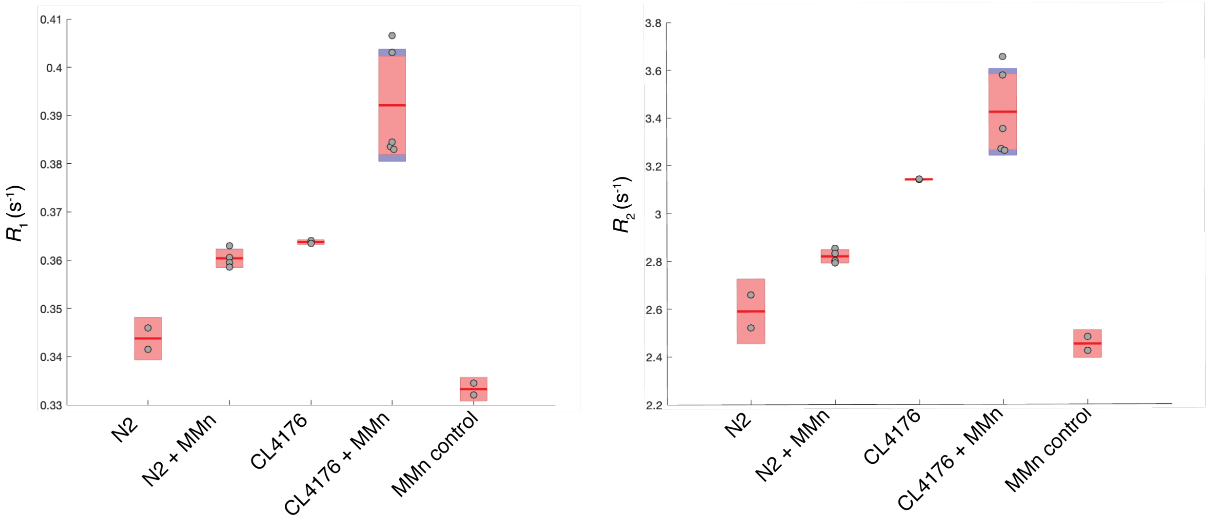

Figure S25. MRI data of *C. elegans* samples in HEPES buffer (1 mg pellet/ 100 μL HEPES).

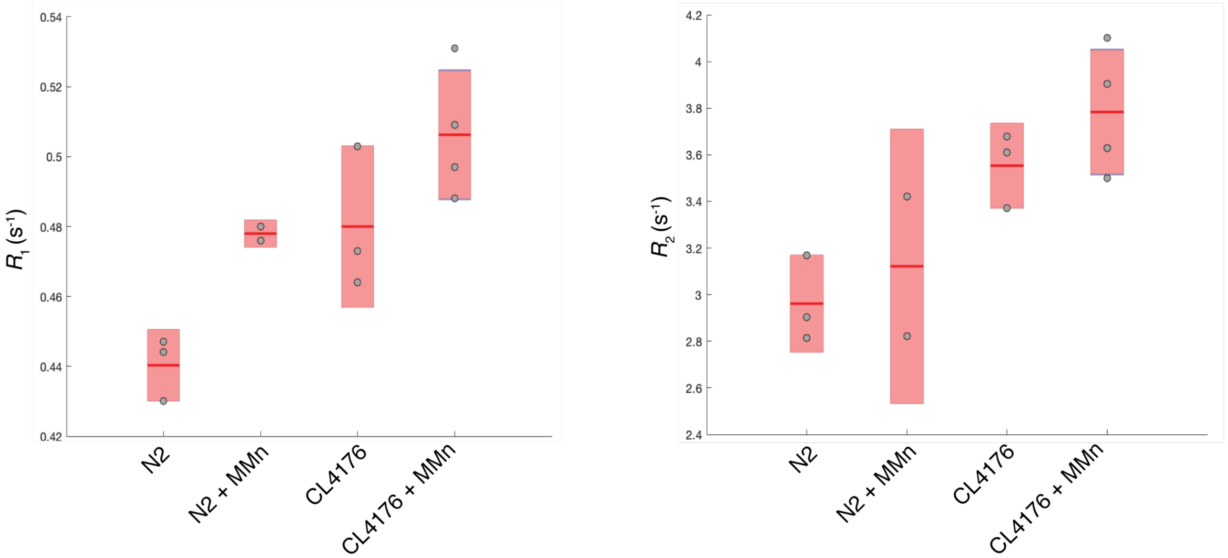

Figure S26. MRI data of a second batch *C. elegans* samples in HEPES buffer (1 mg pellet/ 100 µL HEPES). Differences between experiments may reflect varying levels of metabolic degradation or decomposition following the experiment yielding varying levels of MMn and free manganese ions with a different MRI signal.

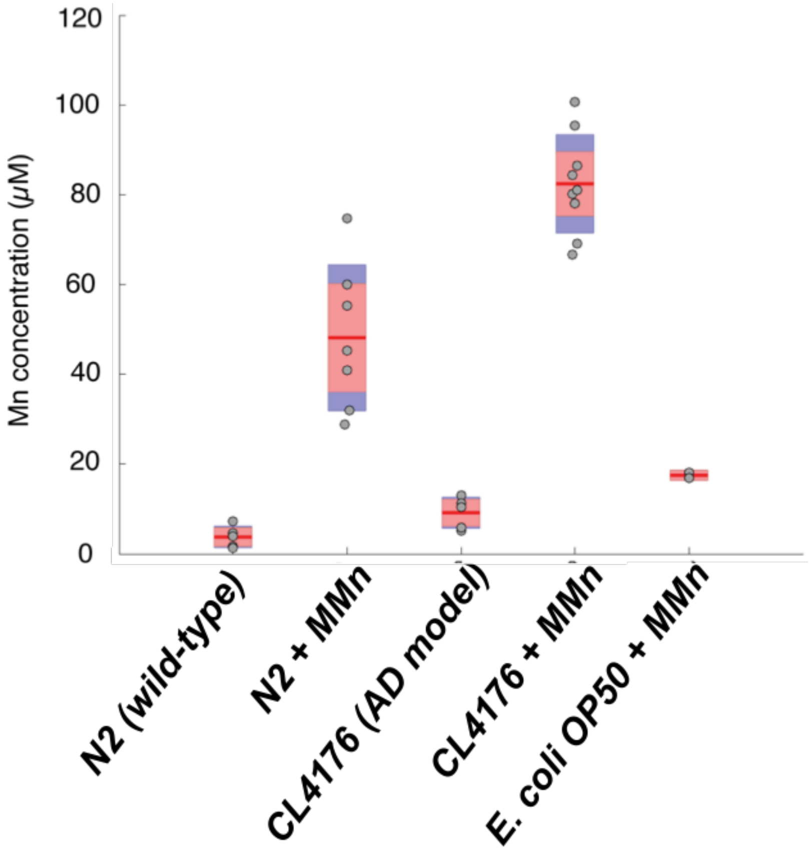

Figure S27. Complete ICP-MS analysis showing wild-type (N2) with and without MMn, AD model (CL4176) with and without MMn, and the "No Worm" control containing only the *E. coli* food source incubated with MMn.

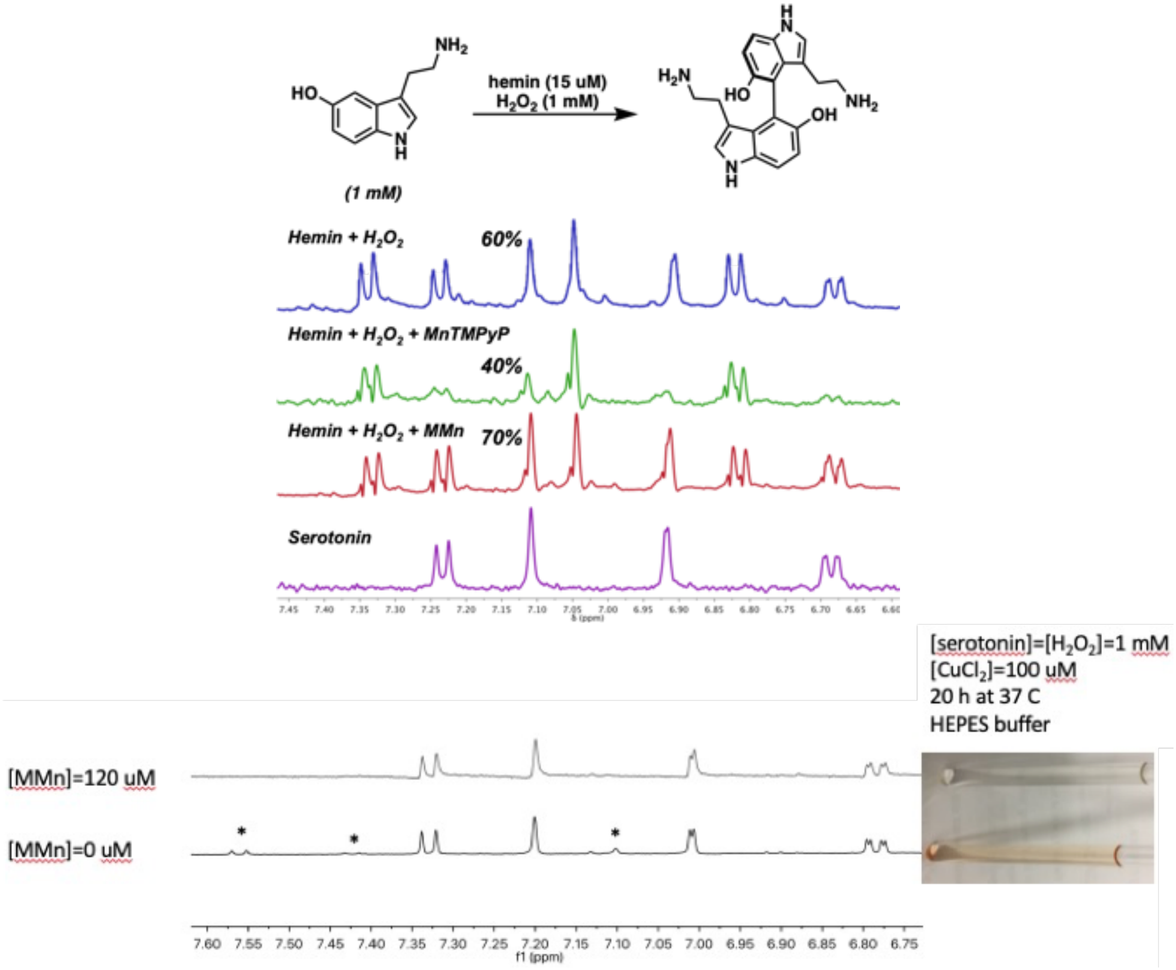

Figure S28. (top) Quantitative ^{1}H NMR spectral analysis of hemin-mediated oxidation of serotonin with MMn and MnTMPyP. (bottom) Qualitative ^{1}H NMR spectral analysis of Cu(II)-mediated oxidation of serotonin with and without MMn.

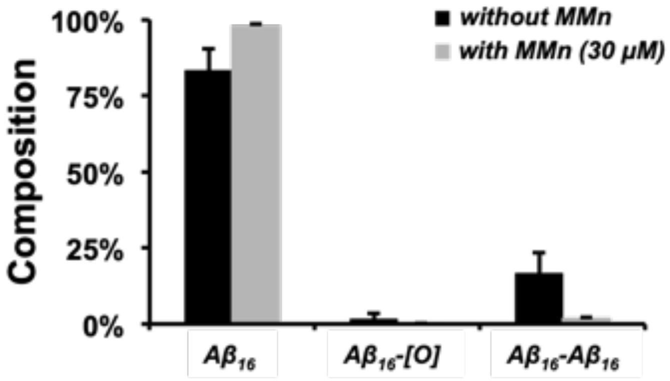

Figure S29. Oxidative modification of $A\beta_{16}$ (300 μM) using hemin (15 μM) and H_2O_2 (1 mM) in 5 mM phosphate buffer (pH 7.4). Precise values with standard deviations were calculated for $A\beta_{16}$, $A\beta_{16}$ containing an oxidized histidine residue (H13/H14), and the $A\beta_{16}$-$A\beta_{16}$ tyrosine dimer. Each experiment is an average of two independent runs. MS/MS spectra of the ion series obtained upon peptide fragmentation confirms that nitration occurs to Tyr10. MS/MS fragmentation also indicated that His13 or His14 are oxidized.

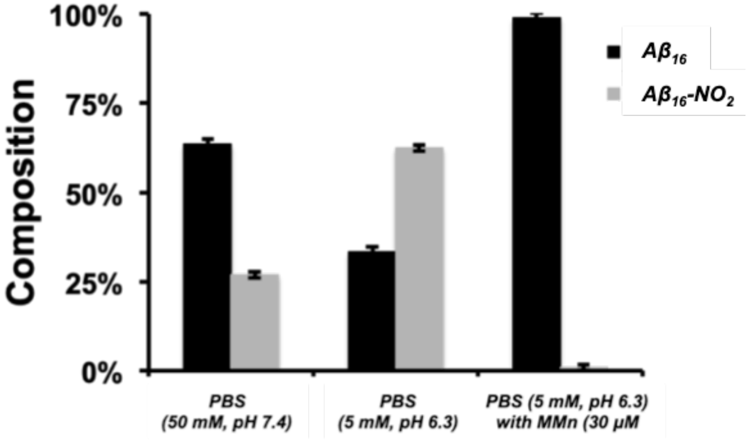

Figure S30. Nitrative modification of Aβ$_{16}$ (300 μM) using hemin (15 μM), H_2O_2 (1 mM), and $NaNO_2$ (1 mM) using 5 and 50 mM phosphate buffer at pH 7.4 and 6.3. Precise values with standard deviations were calculated for Aβ$_{16}$ and Aβ$_{16}$ containing a nitrated tyrosine residue. Each experiment is an average of two independent runs.

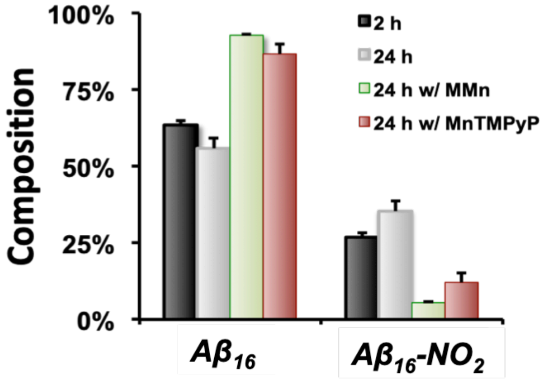

Figure S31. Nitrative modification of Aβ$_{16}$ (300 μM) using hemin (15 μM), H_2O_2 (1 mM), and $NaNO_2$ (1 mM) in 5 mM phosphate buffer at pH 7.4 with and without MMn (30 μM) or MnTMPyP (30 μM). Precise values with standard deviations were calculated for Aβ$_{16}$ and Aβ$_{16}$ containing a nitrated tyrosine residue. Each experiment is an average of two independent runs.

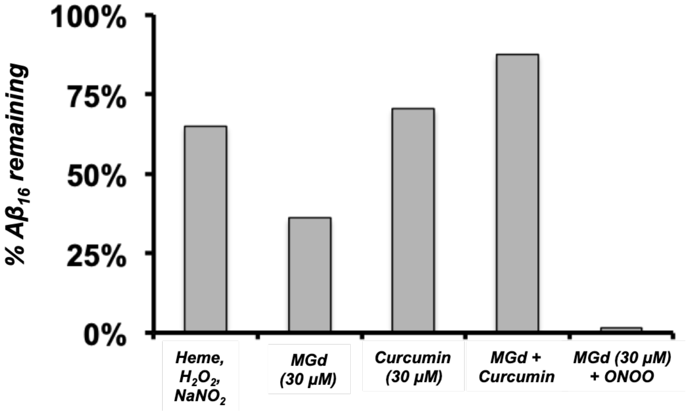

Figure S32. Oxidative and nitrative modification of Aβ$_{16}$ (300 μM) using hemin (10 μM), H_2O_2 (1 mM), and $NaNO_2$ (1 mM) in 5 mM phosphate buffer at pH 7.4 with and without MGd (30 μM), curcumin (30 μM), or MGd and curcumin. Precise values were calculated for Aβ$_{16}$ and oxidized products.

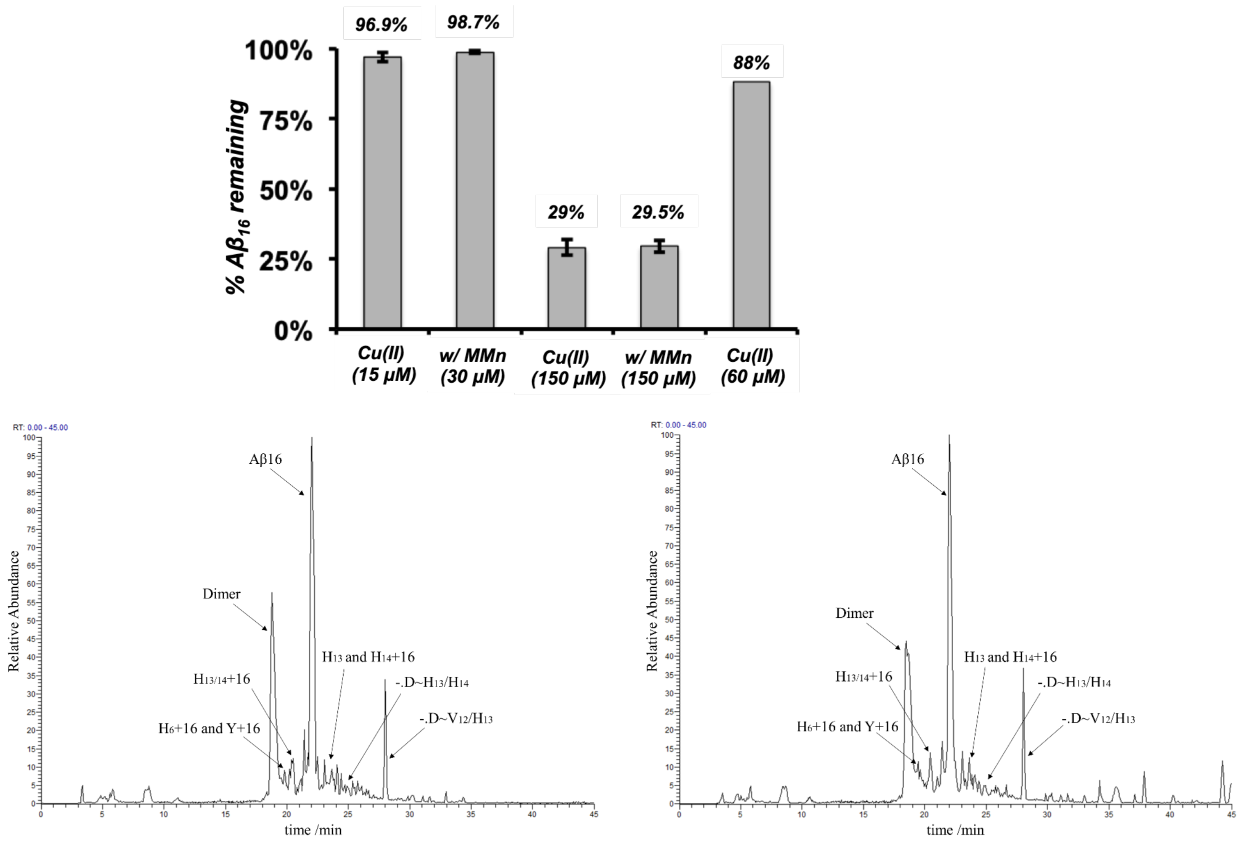

Figure S33. (top) Oxidative modification of Aβ16 (300 μM) using Cu(II) and H_2O_2 (1 mM) in 5 mM phosphate buffer at pH 7.4 with and without MMn (30 μM). Precise values with standard deviations were calculated for Aβ16 and Aβ16 containing a nitrated tyrosine residue. (bottom) HPLC traces using Cu(II) (150 μM) with (*left*) and without MMn (150 μM) (*right*).

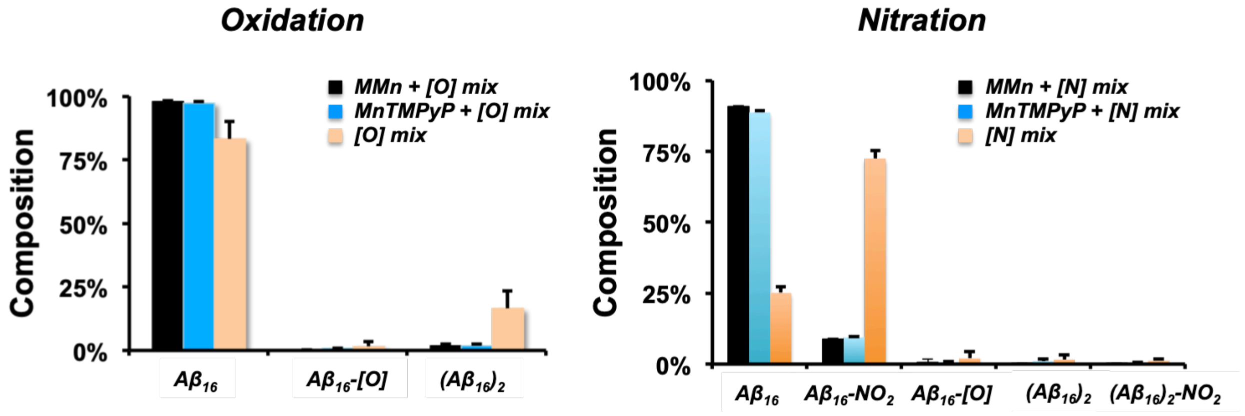

Figure S34. Oxidative and nitrative modification of Aβ16 (300 μM) using hemin (15 μM), H_2O_2 (1 mM), and $NaNO_2$ (1 mM) in 5 mM phosphate buffer at pH 7.4 with and without MMn (30 μM) or MnTMPyP (30 μM). Precise values were calculated for the shown species (*x-axis*). Each experiment is an average of two independent runs.

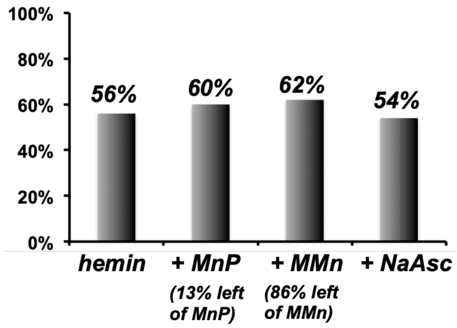

Figure S35. Time dependent percentages for the oxidative decomposition of hemin (15 μM) with and without MnTMPyP (denoted MnP; 5 μM), MMn (5 μM), and sodium ascorbate (40 μM) in PBS (pH 7.15) incubated at 37 °C for 10 minutes.

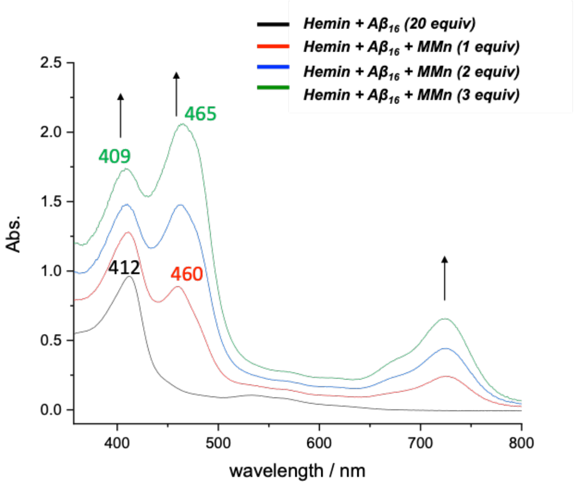

Figure S36. UV-vis spectra (300 K, PBS) showing the effect of MMn (1, 2, and 3 equiv.) on the spectral features of hemin-$(A\beta_{16})_2$. The 2:1 adduct was formed by adding 200 μM of $A\beta_{16}$ to 10 μM of hemin as inferred from the observation of the λ_{max} = 412 nm.

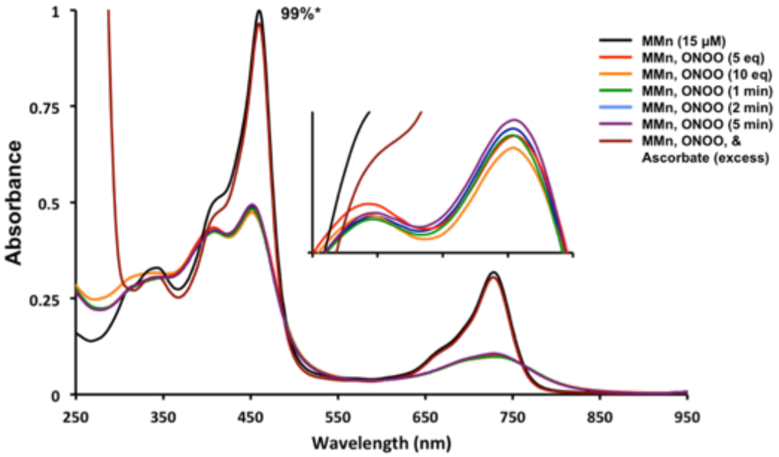

Figure S37. UV-vis spectra of MMn in the presence of peroxynitrite (5 and 10 equiv.) followed by addition of sodium ascorbate (excess) and incubating 5 minutes at room temperature. The insert shows changes to the Mn(**III**) texaphyrin Soret band. **Attributed to dilution*

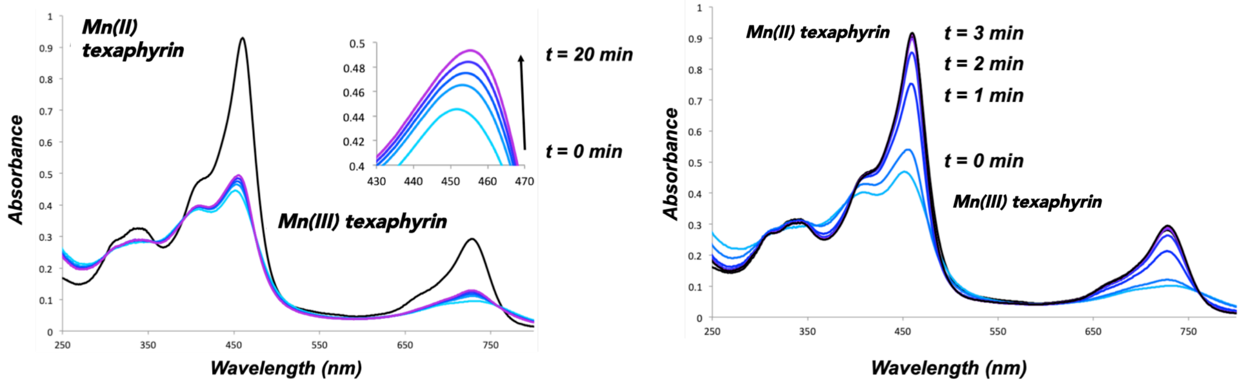

Figure S38. Oxidative disproportionation of H_2O_2 by Mn(III) texaphyrin (15 μM). Mn(III) texaphyrin was prepared by oxidation of Mn(II) texaphyrin with peroxynitrite (150 μM). Auto-reduction in the presence of air going from black (MMn) to light blue to purple (top). Reduction mediated by H_2O_2 going from black (MMn) to light blue to purple (bottom).

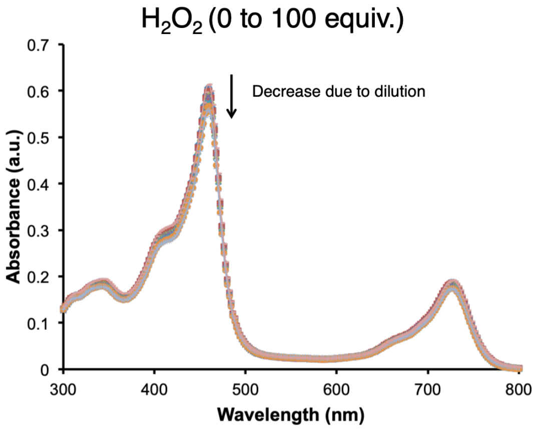

Figure S39. UV-vis spectroscopic titration of Mn(II) texaphyrin (10 μM) with increasing concentrations of H_2O_2 (0 to 100 equiv.).

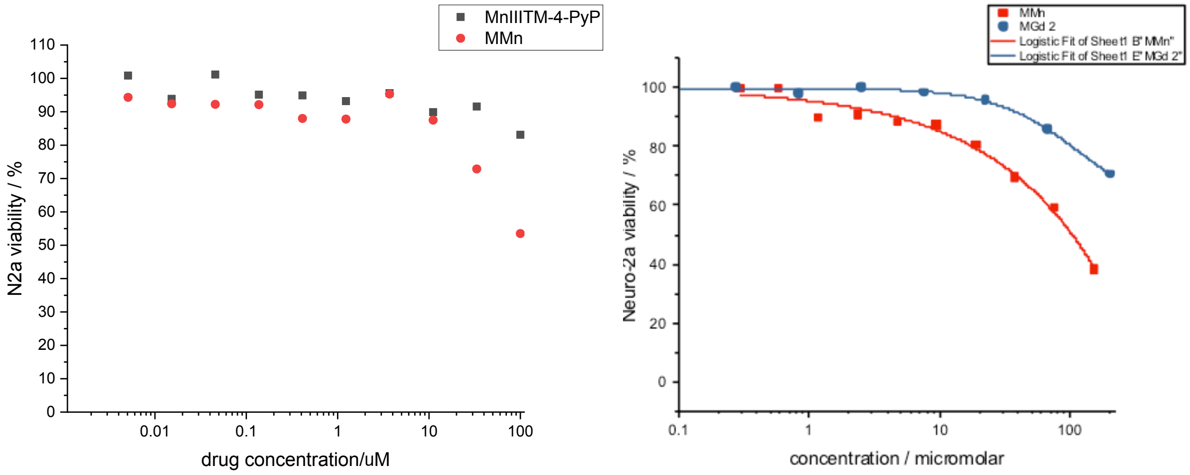

Figure S40. MTT assay measuring the antiproliferative activity against Neuro-2A cells seen after a 24 h exposure time using MMn (red curves, right & left), MnTMPyP (grey square dots, right), and MGd (blue, right).

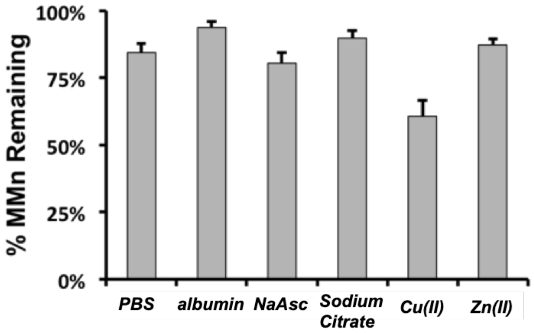

Figure S41. Stability of MMn (5 μM) evaluated by UV-vis spectroscopy after incubating for 24 hours at 37 °C in PBS (5 mM, pH 7.4) while exposed to air: (**1**) in PBS alone, (**2**) with albumin (600 μM), (**3**) with sodium ascorbate (40 μM), (**4**) with sodium citrate (100 μM) as a chelating agent, (**5**) with copper(**II**) (8 μM), and (**6**) with zinc(**II**) (10 μM). Data is an average of three independent runs with error bars showing the standard deviations (SD).

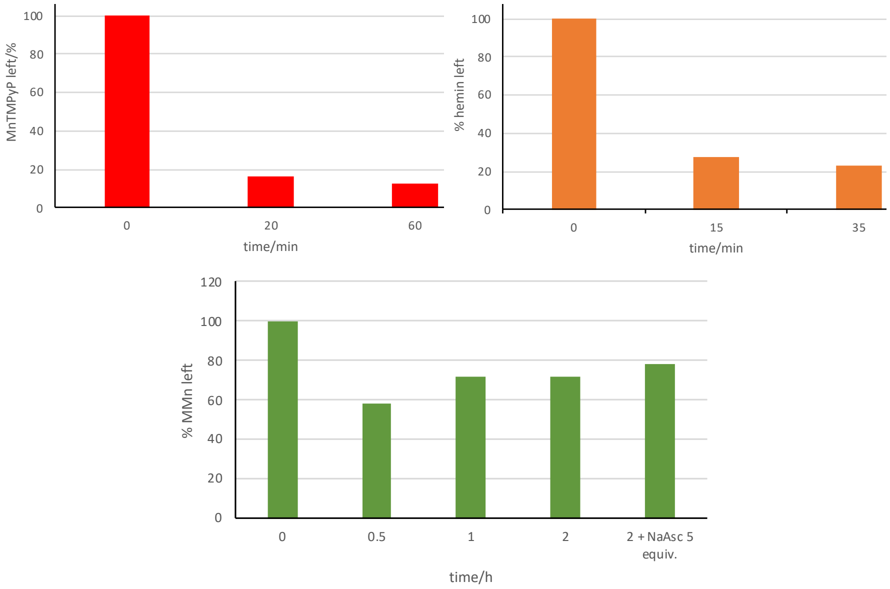

Figure S42. Stability of MnTMPyP (top left), hemin (top right), and MMn (bottom) under oxidative conditions (peroxidase-like activity, [hemin] = 15 □M, [H_2O_2] = 1 mM) with incubation at 37 °C as monitored by means of UV-visible spectroscopy in PBS.

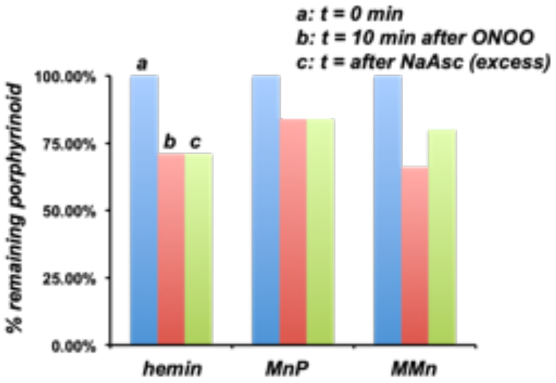

Figure S43. Stability of MnTMPyP (top left), hemin (top right), and MMn (bottom) under oxidative and nitrative conditions using [hemin] = 15 □M, [$ONOO^-$] = 30 □M) with incubation at 37 °C and monitoring by UV-visible spectroscopy in PBS.

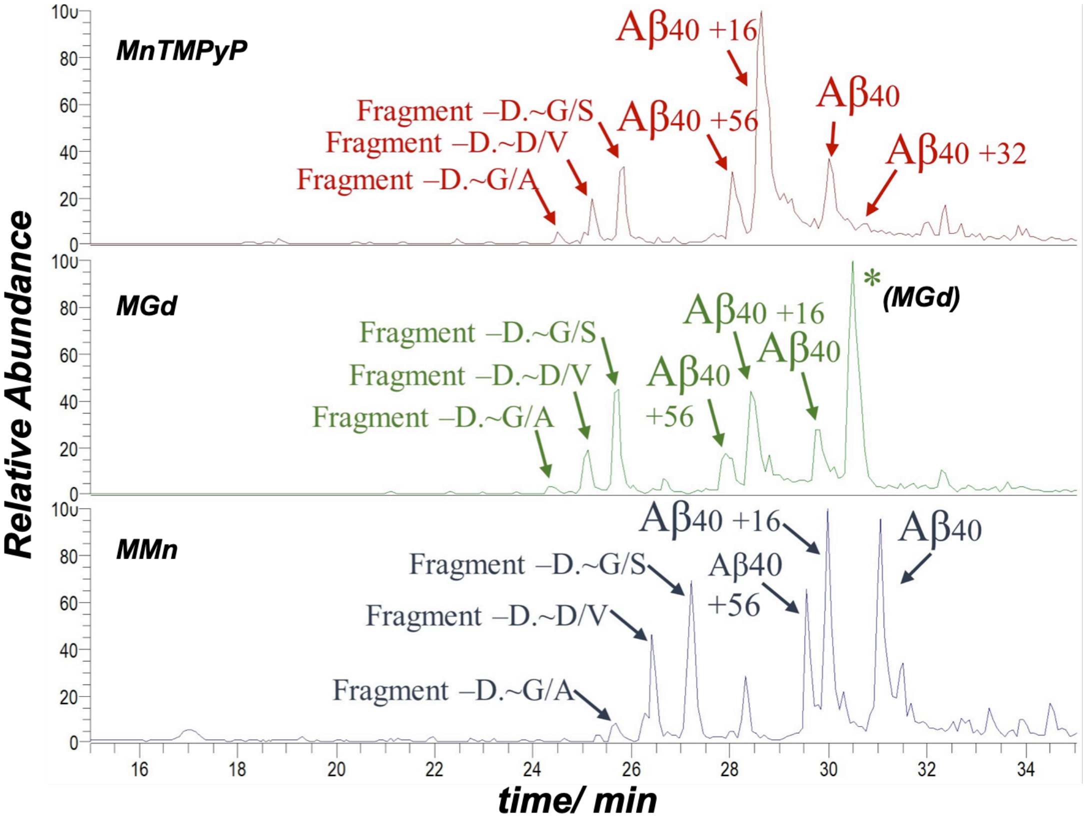

Figure S44. Qualitative HPLC-MS carried out to correlate the level of oxidation of the Aβ_{40} peptide with the cytotoxicity towards N2 cells seen in the MTT assay. MS/MS fragmentation of Aβ_{40}+16 and +32 revealed that oxidative modification is predominately comprised of oxidation at His13/His14 and Met35 with a minor contribution from oxidation at Tyr10.

Supplemental Experimental Procedures

General procedures

Starting materials were purchased from Fisher Scientific or Sigma Aldrich and used without further purification unless otherwise specified. Solvents were purified using a solvent purifier system (Vacuum Atmospheres). Dichloromethane (DCM) was freshly distilled after being dried over CaH_2 under argon. Reaction progress was monitored by thin-layer chromatography (TLC silica gel 60 F_{254}, Silicycle UltraPure Silica gels). Analytical RP-HPLC analyses for MMn and MLu were performed on a Thermo scientific Dionex Ultimate 3000 equipped with a PDA detector. The analytical column was a Syncronis C18 column, 5 μm, 4.6 x 250 mm (Thermo Scientific); the mobile phase consisted of a increasing gradient (from 10% to 99 % in 20 min) of acetonitrile into water, both containing 0.1% acetic acid. In this case the flow rate was 1.2 ml / min. Texaphyrins were monitored at 254, 470, and 740 nm. MMn, MGd, and MLu were purified on reverse phase-tC18 SPE (Waters Sep-Pak, Waters) columns containing 10 g of C-18 using an increasing gradient of acetonitrile in either 0.1 M ammonium acetate/ 1% acetic acid aqueous solution or 0.1 M potassium nitrate aqueous solution as the eluent, depending on which counter anion (AcO^- or NO_3^-) was desired as the ancillary ligand. Mass spectrometric analyses were carried out in The University of Texas at Austin Mass Spectrometry Facility. Low-resolution and high-resolution electro- spray mass spectrometric (ESI-MS) analyses were carried out using Thermo Finnigan LTQ and Qq-FTICR (7 Telsa) instruments, respectively. Inductively coupled plasma mass spectrometry (ICP-MS) was carried out at the Bioimaging and Chemical Analysis Facilities Core of the Center for Environmental Health Sciences at MIT using an Agilent 7900 ICP-MS system. In general, samples were digested for 2 hours at 70 °C in

concentrated nitric acid, before being diluted into 2% nitric acid to a working concentration of 10-100 ppb. Nine external standards (0-500 ppb) of the target metal were used to calibrate the instrument and 10 ppb Er was used as an internal standard in each sample. Samples were run in duplicate or triplicate to minimize systematic errors. ^{1}H-NMR spectra were recorded using a Varian 400 and 600 (solvent suppression) MHz as well as a Bruker AVII HD 500 with Prodigy liquid nitrogen cryoprobe (purchased with NIH grant 1-S10-OD021508-01). UV-Vis spectra were recorded using a Varian Cary 5000 spectrophotometer at room temperature. Fluorescence spectra were recorded on a Photon Technology International Fluorescence Master fluorimeter. The source was a 75 W Xenon short arc lamp. A cell length of 10 mm was used in all UV-Vis and fluorescence studies. Phantom MRI data were acquired in a 12 cm outer diameter birdcage transceiver for imaging in a 20 cm bore Bruker 7 T Avance III MRI scanner. Samples at varying concentrations (0 up to 500 μM), as determined by inductively coupled plasma mass spectrometry (ICP-MS) in HEPES (10 mM, pH 7.4) or Dulbecco's PBS (as supplied) were loaded into the wells of a 384-well clear polystyrene plate (Thermo Scientific™ Nunc™), which had been pre-cut in half to optimally fit the coil. Unused wells were filled with buffer. A 2 mm slice was imaged through the samples with the field of view of 5 x 5 cm and the data matrices were 256 x 256 points. Longitudinal (R_1) and transverse (R_2) relaxation measurements were acquired using multi-spin multi-echo (MSME) sequences (flip angle = 180°). R_1 parameters; TE = 12 ms, TR = 100, 200 300, 400, 500, 600, 700 800, 900, 1000, 1200, 1500, 3000, 5000 ms. R_2 parameters; TR = 5000 ms, TE = 12, 24, 36, 48, 60, 72, 84, 96, 108, 120, 132, 144, 156, 168, 280, 192, 204, 216, 228, 240, 252, 264, 276, 288, 300, 312, 324, 336, 348, 360 ms. Custom routines written in Matlab (Mathworks, Natick, MA) were used to reconstruct the images and compute relaxation time constants by fitting image intensity data to exponential decay curves. Relaxivity values (r_1 & r_2) were generated from the gradient of the slope obtained by plotting the calculated relaxation rates against the concentration of the agent in each well. Electron Paramagnetic Resonance (EPR) spectroscopy was carried out using a Bruker EMX Plus spectrometer, X-band (9.5 GHz). Experimental settings were amplitude modulation = 10 G and microwave power = 2 mW. The observed spectra were taken as an average of four scans. Circular dichroism (CD) analyses were carried out using a Jasco J-815 CD spectrometer in The University of Texas at Austin Targeted Therapeutic Drug Discovery & Development Program (Ti3D). The data shown in this report was taken as an average of 3 scans from 260 to 195 nm using a 0.05 nm wavelength step. SEM analyses were prepared by drop casting 20 μM samples onto silica wafers (University Wafers) then gently dried with nitrogen. Images were taken on a FEI Quanta 650 ESEM in The University of Texas at Austin-Texas Materials Institute. There are inherent difficulties in working with $A\beta_{40}$ that are often ignored (e.g., aggregation state, solubility, left over additive to solubilize the peptide, etc.). The methods discussed in the ESI proved reproducible throughout the studies and when carried out by multiple authors.

Peptide synthesis. Aβ peptides were prepared via Fmoc amino solid-phase peptide synthesis using a Liberty Blue microwave peptide synthesizer. Preparative RP-HPLC purification of peptides was performed using an Agilent Zorbax SB-C_{18} Prep HT column 21.2 × 250 mm. Analytical RP-HPLC characterization of peptides was performed using an Agilent Zorbax column 4.6 × 250 mm. An Agilent Technologies 6530 Accurate Mass QT of LC/MS was used for high-resolution mass spectra of purified peptides. Solvents used were HPLC grade.

Synthesis and characterizations of metallotexaphyrins MGd, MLu and MMn. Motexafin gadolinium (MGd) was a gift from Pharmacyclics, Inc., recently acquired by Abbvie. MLu and MMn were synthesized using literature protocols.[S1,S2] The purity of MMn, MLu, and MGd was checked by reverse phase-HPLC before use.

1-Octanol: water partition experiments gave the following data: LogP(MGd) = -0.95; LogP(MMn) = 0.69

Solutions of $A\beta_{40}$ peptide. Approximately 2 mg of $A\beta_{40}$ (as a lyophilized powder) was dissolved in 1 mL aqueous NaOH (10 mM). After vortexing and sonication, the solution was filtered through a 0.22 μm PES syringe filter. PBS (pH = 7.15) was added to obtain a final concentration of 100 μM $A\beta_{40}$. The concentration was checked by UV-vis spectrophotometry using the absorbance at 280 nm and a molar absorptivity of 1490 M^{-1} cm^{-1}. A similar procedure was used for the quantitative reactivity studies with the final dilution being in miliQ water to give a stock solution of approximately 600 μM.

Cell uptake experiments. Neuro-2A cells were grown until confluent in T-75 flasks. MMn was added (100 µM) and cells were incubated for 9 h, upon which cells were washed twice with PBS, incubated with trypsin (1X) for 2 minutes, diluted with supplemented EMEM, counted, and pelleted by centrifugation (3 min at 2000 rpm). After supernatant removal, the pellet was suspended/washed one more time with PBS (5 ml) and the pellet was reformed by centrifugation (3 min at 2000 rpm). The cell pellet was digested in concentrated nitric acid (100 µL) for 15 h at 60°C and diluted with miliQ water to a final concentration of 2% HNO_3 before being analyzed by ICP-MS.

Caenorhabditis elegans. The *C. elegans* strains N2 and CL4176 were provided by CGC (nematodes shipped on 35 mm diameter plates), which is funded by the NIH Office of Research Infrastructure Programs (P40-OD010440). *C. elegans* were grown and maintained as described in "WormBook: The Online Review of *C. elegans* Biology" by Theresa Stiernagle (https://www.ncbi.nlm.nih.gov/books/NBK19649/).

Nematode Growth Medium (NGM). NaCl (3 g), agar (17 g) and peptone (2.5 g) were added to 975 ml of H_2O in a 2 L Erlenmeyer flask, which was then autoclaved for 1 h. All work after this was completed under sterile conditions. The flask was cooled and maintained at 60 °C in a water bath to prevent the solution from solidifying. To this heated solution was added $CaCl_2$ (1 ml of a 1 M solution), cholesterol (1 ml of a solution in ethanol at 5 mg/ mL), $MgSO_4$ (1 ml of 1 M solution), and KPO_4 buffer (25 ml of 1 M solution). The NGM solution was dispensed into 10 mm diameter plates (15 ml/plate). The plates were left at room temperature for 1-2 days to allow excess moisture to evaporate. Plates showing contamination were discarded.

***E. coli* OP50 culture & plate.** Lysogeny broth (LB) was prepared by mixing 21 g LB powder (Sigma Aldrich) in 1 L of Milli Q water. LB agar was prepared by adding 17 g/ L of agar (Fisher Sci.) to the LB mixture. Both the broth and agar were autoclaved at 121 °C for 45 minutes. All work after autoclaving was conducted under sterile conditions. A bead of *E. coli* OP50 (BactoBeads, Fisher Sci.) was streaked on an LB agar plate and grown in a static incubator overnight at 37 °C. From this plate, a single colony was isolated and inoculated in 10 mL of LB. The mixture was placed in a shaking incubator heated to 37 °C overnight. From this NMS plates could be prepared by pipetting 1 mL of the overnight culture onto NMS agar. The plate was swirled to spread the culture and the seeded plates were incubated at room temperature overnight. NMR plates were found to have a lawn of *E. coli* OP50 and were stored inverted at 4 °C, with an up to 3-week shelf life.

MMn uptake experiment. After receiving the *C. elegans* animals, N2 and CL4176, from the CGC (University of Minnesota) shipped on petri dishes, agar matrix was cut in small chunks (0.5 x 0.5 cm) with a razor blade and transferred on Nematode Growth Medium (NGM) agar plates previously seeded with *E. coli* strain OP50 as a food source. NGM plates were then placed at 16 °C in order to increase the population of *C. elegans* before drug exposure and prevent significant plaque formation in the CL4176 AD model. After 7 days, MMn was added to plates (1 ml/ plate of a solution at 100 µM), which were incubated at 25 °C for 48 hours. The animals were then transferred to Eppendorf tubes (previously weighted empty on analytical balance) using 1.5 ml of water. The tubes were put on ice for 5 min and centrifuged (14000 rpm for 1 min) and the supernatant was removed. The plate was washed again with 1.5 mL of water and centrifuged again. The resulting pellets were washed twice with 1 ml of DI water. *C. elegans* pellets were lyophilized over a period of 15 h before being weighed (dry pellet weights obtained by lyophilization were used to normalize the MRI and ICP-MS results).

Preparation of peroxynitrite solution. Three separate solutions of $NaNO_2$ (0.6 M), HCl (0.68 M) with H_2O_2 (0.72 M), and NaOH (3.6 M) were prepared and stored in the freezer at -20 °C for thirty minutes. The solutions were transferred to an ice bath and allowed to sit for thirty minutes. The $NaNO_2$ solution was added to a 100 mL beaker placed in an ice bath and stirred at > 500 rpm. The HCl/ H_2O_2 solution and NaOH solutions are added sequentially with the NaOH coming immediately after the HCl/ H_2O_2 solution. *These solutions must be added in sequence (i.e. one then the other) with as little time between the two additions as possible. Best results were obtained by adding the two solutions with a very small offset.* The mixture turned yellow and was allowed to stir in the ice bath for 5 minutes. MnO_2 was added portion wise

until gas evolution ceased. The mixture was split into aliquots, centrifuged, and the peroxynitrite solution was decanted using a glass pipette. The concentration could be calculated using λ_{max} = 302 nm with ε = 1670 $M^{-1}cm^{-1}$. Best results were obtained by centrifugation in a cold room at 4 °C. [peroxynitrite] = 72-86 mM.

Hemin-Serotonin Procedure. Serotonin (1 mM), H_2O_2 (1 mM), hemin (15 μM), and sodium ascorbate without and with MMn (120 μM) or MnTMPyP (120 μM) were incubated in PBS (pH 7.4, 1.5 mL total volume) at 37 °C for 2 hours in the dark. An analogous experiment without sodium ascorbate using the same concentrations of serotonin, H_2O_2, hemin, and MMn or MnTMPyP. The resulting mixture was passed through a reverse phase-tC18 SPE (Waters Sep-Pak, Waters) column containing 10 g of the C-18 substrate. The column was washed with 25 mL of 5% acetonitrile: H_2O to separate the serotonin and 4,4'-serotonin dimer from paramagnetic species. The resulting solution was concentrated using a rotary evaporator. The mixture was taken up in D_2O (0.7 mL) and analyzed via ^1H NMR spectroscopy. NMR spectroscopic yields were calculated by comparing the relative concentration of serotonin to the 4,4'-serotonin dimer. ***Note***: Additional washing of the column failed to yield additional serotonin or the 4,4'-serotonin dimer.

Cu(II) Procedure. Serotonin (1 mM), H_2O_2 (1 mM), $CuCl_2$ (100 μM) without and with MMn (120 μM) or MnTMPyP (120 μM) were incubated in PBS (pH 7.4, 1.5 mL total volume) at 37 °C for 20 hours in the dark. The resulting mixture was passed through a reverse phase-tC18 SPE (Waters Sep-Pak, Waters) column containing 10 g of the C-18 substrate. The column was washed with 25 mL of 5% acetonitrile: H_2O to separate the serotonin and 4,4'-serotonin dimer from paramagnetic species. The resulting solution was concentrated using a rotary evaporator. The mixture was taken up in D_2O (0.7 mL) and analyzed via ^1H NMR spectroscopy. NMR spectroscopic yields were calculated by comparing the relative concentration of serotonin to the 4,4'-serotonin dimer.

Studies of $A\beta_{16}$ & $A\beta_{40}$-hemin-H_2O_2 with and without $NaNO_2$. Hemin (15 μM), $NaNO_2$ (1 mM), H_2O_2 (1 mM), and $A\beta_{16}$ (300 μM) or $A\beta_{40}$ (100 μM) in PBS (pH 7.4, 5 mM, 200 μL total volume) were incubated together at 37 °C for 2 h, unless otherwise specified. The resulting mixtures were subjected to HPLC-MS analysis. Vials were cooled to 4 °C inside the HPLC-MS to prevent additional reactivity.

Studies of $A\beta_{16}$-Cu(II)-H_2O_2. $CuCl_2$ (15, 60, and 150 μM), H_2O_2 (1 mM), and $A\beta_{16}$ (300 μM) with and without MMn (30 and 150 μM) in PBS (pH 7.4, 5 mM, 200 μL total volume) were incubated together at 37 °C for 2 h. The resulting mixtures were subjected to HPLC-MS analysis. Vials were cooled to 4 °C inside the HPLC-MS to prevent additional reactivity.

Studies of $A\beta_{16}$-hemin-peroxynitrite. Hemin (15 μM), peroxynitrite (1 mM) and $A\beta_{16}$ (300 μM) in PBS (200 μL total volume) were incubated together at 37 °C for 30 min. All compounds were added and then vortexed to provide a homogenous mixture. Immediately after adding peroxynitrite, the mixture was vortexed again and allowed to incubate at 37 °C for 30 minutes. The resulting mixtures were immediately subjected to HPLC-MS analysis. Vials were cooled to 4 °C inside the HPLC-MS queue to prevent additional reactivity.

HPLC-MS/MS analyses. Analytical RP-HPLC characterization of peptides was performed using an Agilent Zorbax column 4.6 × 250 mm. An Agilent Technologies 6530 Accurate Mass QT of LC/MS was used for high-resolution mass spectra of purified peptides. Solvents used were HPLC grade. UV-vis spectra were recorded on a Hewlett Packard HP 8453 A diode array spectrophotometer equipped with a thermostated, magnetically stirred cuvette holder. The peptides were studied by direct HPLC-MS/MS analysis. LC-MS and LC-MS/MS data were obtained by using an LCQ DECA ion-trap mass spectrometer with an ESI ion source, coupled with an automatic injector Surveyor HPLC system and controlled by Xcalibur 1.3 software (Thermo-Finnigan, San Jose, CA). The system was run in an automated LC-MS/MS mode and by using a Surveyor HPLC system (Thermo-Finnigan, San Jose, CA, USA) equipped with a BIOBASIC C18 column (5 μM), 150 x 2.1 mm). For analysis of oxidation studies, the acquired MS/MS spectra were automatically searched against the human $A\beta_{16}$ sequence by using the SEQUEST algorithm

to identify the modified residues. This algorithm was incorporated into the Bioworks 3.1 software (Thermo-Finnigan, San Jose, CA). The elution was performed with a 0-55% linear gradient over 65 min with 0.1% formic acid in water as solvent A and 0.1% formic acid in acetonitrile as solvent B with a flow rate of 0.2 mL/min. For analysis of oxidation studies, the acquired MS/MS spectra were automatically searched against the human $A\beta_{16}$ sequence by using the SEQUEST algorithm to identify the modified residues. This algorithm was incorporated into the Bioworks 3.1 software (Thermo-Finnigan, San Jose, CA).

MS/MS spectrometric analyses of the $A\beta_{16}$ ion series samples provided support for the conclusion that nitration occurs to Tyr10. MS/MS fragmentation of $A\beta_{16}$ revealed that His13 or His14 are the major oxidized species with dimerization occurring at Tyr10. MS/MS fragmentation of $A\beta_{40}$ revealed that oxidative modification is predominately comprised of oxidation at His13/His14 and Met35 with Tyr10 oxidation being minimal.

Cell culture. The neuroblastoma cell line Neuro-2A was bought from ATCC. Cells were grown in EMEM medium containing 10% heat inactivated fetal bovine serum (FBS) and antibiotics (100 mg/ ml streptomycin and 100 U/ ml penicillin) at 37 °C, under 5% CO_2 atmosphere. The cells were sub-cultured once every 4 to 5 days.

MTT viability assay (determination of IC_{50} values for MMn, MnTMPyP, and MGd). The proliferation of exponential phase cultures of Neuro-2A was assessed by tetrazolium (MTT) dye reduction. In brief, tumor cells were seeded in 96-well microliter plates at 10,000 cells per well and allowed to adhere overnight in the culture medium described previously. After 15 h, agents were added via serial dilution (from 200 to 0.01 μM) and the plate was incubated at 37°C and 5% CO_2. After 24 h incubation time with the agent in question (2 days overall) 50 μL of a solution of tetrazolium dye in EMEM without FBS (3 mg/ mL) was added to each well. The plate was incubated for 4 hours, whereupon the medium was removed and the formazan product dissolved in 50 μL of DMSO. The absorbance at 570 nm was measured using a microplate reader (Molecular Devices, Sunnyvale, CA), corrected for background and then normalized to wells containing untreated cells to allow plate-to-plate comparisons. The growth inhibition data were fitted to a sigmoidal dose-response curve (logistic function, origin software) to generate IC_{50} values (defined as the concentrations of drug inhibiting cell growth by 50%). The IC_{50} is presented as mean +/- standard deviation.

24-well plate MTT experiments. $A\beta_{40}$ (90 μM) was incubated in PBS alone or in the presence of H_2O_2 (1 mM), hemin (15 μM), $NaNO_2$ (1 mM) (referred to as nitration mixture) for 3 h at 37 °C followed by 24 h at 4 °C. 250 μL of each of the solution was transferred into each well containing about 50,000 Neuro-2A cells (plated 24 h earlier) in 250 μL of supplemented EMEM medium without phenol red. After 24 h, the medium was removed and replaced with 500 μL of fresh EMEM not containing FBS and phenol red. To this was added, 200 μl of an MTT solution at 3 mg/ml in EMEM not containing phenol red and FBS. After 4 h at 37 °C, the supernatant was removed and 200 μl of DMSO was added to each well to dissolve the formazan purple crystals. Aliquot (50 μl) of each well were transferred into a 96-well plate well and the absorbance at 570 nm was read using a plate reader (see description above).

References

[S1] Sessler, J. L., Mody, T. D., Hemmi, G. W., and Lynch, V. (1993) Synthesis and structural characterization of lanthanide(III) texaphyrins. *Inorg. Chem. 32*, 3175-3187.

[S2] Hannah, S., Lynch, V.M., Guldi, D. M., Gerasimchuk, N., MacDonald, C. L. B., Magda, D., and Sessler, J. L. (2002) Late First-Row Transition-Metal Complexes of Texaphyrin. *J. Am. Chem. Soc. 124*, 8416-8427.
