# Supplementary material for: Metallotexaphyrins as MRI-Active Catalytic Antioxidants for Neurodegenerative Disease: A Study on Alzheimer’s Disease
Source: Chem. 2020 Mar 12;6(3):703–24. doi: 10.1016/j.chempr.2019.12.016 (PMC7074011; doi:10.1016/j.chempr.2019.12.016)
Supplement: Document S2. Article plus Supplemental Information [file mmc2.pdf]

Chem

CellPress

Article

Metallotexaphyrins as MRI-Active Catalytic Antioxidants for Neurodegenerative Disease: A Study on Alzheimer's Disease

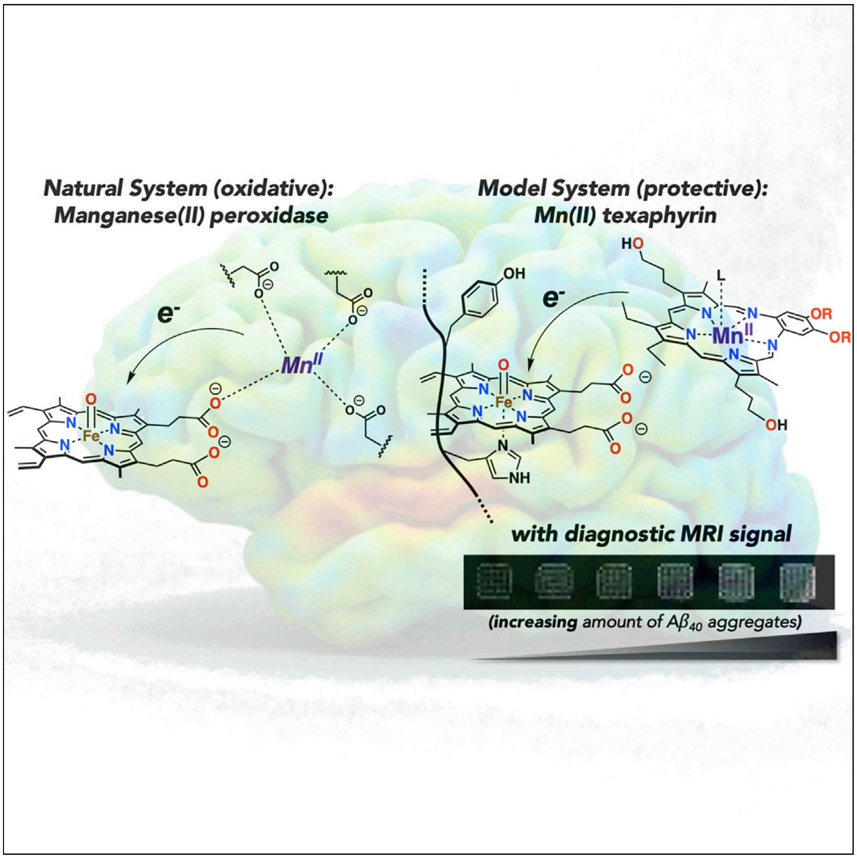

Sessler et al. have studied manganese(II) texaphyrin as a catalytic metalloantioxidant theranostic agent for the detection of Aβ aggregates. A series of *in vitro* and *in vivo* analyses allowed for the inference that metallotexaphyrins could be used for MRI-based detection of Aβ biomarkers. *In vitro* and cellular-based assays coupled with mechanistic analysis demonstrated an efficacious protective effect against oxidative and nitrative damage. The present results could thus guide future efforts in developing new theranostic modalities for use in neurodegenerative disease.

James T. Brewster II, Gregory D. Thiabaud, Peter Harvey, ..., Alan Jasanoff, Luigi Casella, Jonathan L. Sessler

peter.harvey@nottingham.ac.uk (P.H.)
simone.dellacqua@unipv.it (S.D.)
luigi.casella@unipv.it (L.C.)
sessler@cm.utexas.edu (J.L.S.)

HIGHLIGHTS

Manganese texaphyrin binds to Aβ constructs (i.e., monomer, fibrils, and aggregates)

MRI studies demonstrate the utility of metallotexaphyrins for detecting Aβ aggregates

Antioxidant assays show potential utility of MMn for treating CNS neurodegeneration

Brewster et al., Chem 6, 703–724
March 12, 2020 © 2019 The Author(s). Published by Elsevier Inc.
https://doi.org/10.1016/j.chempr.2019.12.016

Chem

CellPress

Article

Metallotexaphyrins as MRI-Active Catalytic Antioxidants for Neurodegenerative Disease: A Study on Alzheimer's Disease

James T. Brewster II,[1,8] Gregory D. Thiabaud,[1,8] Peter Harvey,[2,3,*] Hadiqa Zafar,[1] James F. Reuther,[1,4] Simone Dell'Acqua,[5,*] Rachel M. Johnson,[6] Harrison D. Root,[1] Pedro Metola,[6] Alan Jasanoff,[2] Luigi Casella,[5,*] and Jonathan L. Sessler[1,7,9,*]

SUMMARY

The complex etiology of neurodegeneration continues to stifle efforts to develop effective therapeutics. New agents elucidating key pathways causing neurodegeneration might serve to increase our understanding and potentially lead to improved treatments. Here, we demonstrate that a water-soluble manganese(II) texaphyrin (MMn) is a suitable magnetic resonance imaging (MRI) contrast agent for detecting larger amyloid beta constructs. The imaging potential of MMn was inferred on the basis of *in vitro* studies and *in vivo* detection in Alzheimer's disease *C. elegans* models via MRI and ICP-MS. *In vitro* antioxidant- and cellular-based assays provide support for the notion that this porphyrin analog shows promise as a therapeutic agent able to mitigate the oxidative and nitrative toxic effects considered causal in neurodegeneration. The present report marks the first elaboration of an MRI-active metalloantioxidant that confers diagnostic and therapeutic benefit in Alzheimer's disease models without conjugation of a radioisotope, targeting moiety, or therapeutic payload.

INTRODUCTION

Many seminal advances in physiology and medicine have been made by hewing to a reductionism-centered mantra wherein discrete biological functions are attributed to individual processes. However, an increased understanding of the complex interactions between cellular components (e.g., DNA, RNA, proteins, and small molecules) is leading to a more nuanced view.[1] For instance, it is now recognized that a number of key biological effects arise from interaction webs involving multiple biochemical processes operating within a scale-free network.[2] Such an appreciation is considered particularly relevant to the problem of understanding the central nervous system (CNS) and diseases that affect it.[3]

Among CNS disorders, neurodegeneration is particularly insidious. It defines a series of complex disease states that lead to death. Current therapies, at best, ameliorate the symptoms with limited long-term benefit to patients or relief to caregivers.[4] The complex etiology of neurodegeneration derives from known and unknown initiators operating simultaneously within a feedback loop that is thought to exacerbate downstream toxicity.[5] In particular, Alzheimer's disease (AD) has been postulated to arise from abnormal protein deposits, excitotoxicity, disruption of metal-ion homeostasis, reduction in endogenous antioxidants, and neuroinflammation, among other hypotheses.[6] Most, if not all, of these putative malfunctions are correlated with an increase in reactive oxygen species (ROS) and reactive nitrogen species (RNS).[7]

The Bigger Picture

Neurodegenerative disease is correlated with aberrant oxidation and nitration of small-molecule neurotransmitters, proteins, DNA, and cellular membrane components. Cellular damage enhanced by these modifications propagates disease progression, leading to death. Effective tools that enable insight into the complex etiology of CNS diseases could allow researchers to find a cure. Part of this effort is focused on developing imaging modalities that can assist in understanding these diseases. Here, we demonstrate a new theranostic strategy that shows promise in addressing the oxidative and nitrative malfunctions considered causal in disease progression. The approach detailed in this report also allows the MRI of aggregated Aβ, which could lead to improvements in disease diagnosis. The present work is thus expected to set the stage for the mechanistic-based design of theranostics, useful in understanding and treating neurodegeneration.

© 2019 The Author(s). Published by Elsevier Inc.
This is an open access article under the CC BY license (http://creativecommons.org/licenses/by/4.0/).

Both ROS and RNS are thought to disrupt standard cellular processes giving rise to dysfunction and neuronal cell death.[8] Here, we report that a first-generation water-soluble manganese(II) texaphyrin (MMn) displays cell permeability and allows for the detection of larger order amyloid beta (Aβ) constructs (i.e., aggregates) by means of magnetic resonance imaging (MRI). Inductively coupled plasma-mass spectrometry (ICP-MS) and MRI analyses of *in vivo* (*C. elegans*) AD models provided further support for this notion. Based on *in vitro* mechanistic studies, this texaphyrin system also mitigates the oxidative and nitrative toxicity effects considered causal in CNS neurodegeneration. It thus shows promise as a new tool that may aid in understanding, imaging, and treating neurodegenerative disease.

Deciphering the precise biochemical deviations from a normal network that are associated with CNS neurodegeneration is considered key to finding a cure.[9–11] Within this paradigm, significant efforts have focused on developing accurate biomarkers through cerebrospinal fluid (CSF) protein analysis and positron emission tomography (PET) imaging.[12,13] These test methods have proven useful in furthering our understanding of disease progression; however, they can be invasive and expensive. MRI has emerged as a potential complement to CSF and PET analyses.[14] Recent efforts have also focused on the use of theranostics, wherein a fluorophore, MRI-active metal ion, or radioisotope is conjugated with a therapeutic functionality.[15] Many of these conjugates have relied on either Pittsburgh compound B (PiB)- or curcumin-based systems for imaging combined with an Aβ aggregation inhibitor to provide therapeutic efficacy. MR-based imaging systems for the study of neurodegeneration have generally relied on Gd-DOTA or related aminopolycarboxylate ligands conjugated to antibodies or other targeting moieties. Cell and blood-brain-barrier permeability remains a major issue in such agents and could represent a serious impediment to regulatory approval and ultimate clinical utility.[16]

Expanded porphyrins, porphyrin-like systems containing a larger internal cavity than natural tetrapyrrolic congeners, have received considerable attention in recent years.[17] One such system is texaphyrin, a penta-aza Schiff base macrocycle.[18] Compared with porphyrins, texaphyrins contain an approximately 20% larger core and display a unique electronic structure. These systems also exhibit efficacious biological activity with cell permeability and potential in human medicine.[19] In fact, specific water-soluble gadolinium(III) and lutetium(III) texaphyrin derivatives, known as MGd and MLu, respectively, were explored early on as potential therapeutics; the pro-oxidative MGd was studied through phase III clinical trials as a radiosensitizer, and MLu was studied through phase I clinical trials for photoangioplasty.[20–22] Notably, MGd demonstrated a high maximum tolerated dose (22.3 mg kg^{-1}) and a median half-life of 7.4 h and could be detected within brain metastases even 14 h after administration, as determined by MRI. Recently, dual-modal MGd-platinum(IV) conjugates have shown promise as potential cancer drug leads.[23]

Mn(III) porphyrin and corrole metalloantioxidants have demonstrated an efficacious therapeutic effect that is thought to be mediated via decomposition of peroxynitrite ($ONOO^-$) and, in some instances, superoxide and hydrogen peroxide (H_2O_2) (Figure 1).[24] In particular, AEOL-10150, an octaethyl imidazolium Mn(III) porphyrin, has shown broad-spectrum superoxide dismutase (SOD)-like activity in animal models of amyotrophic lateral sclerosis (ALS), idiopathic pulmonary fibrosis, and nerve agent exposure (Figure 1).[25] AEOL-10150 has also successfully completed a phase I clinical trial in ALS and healthy patients.[26] Recent studies involving a water-soluble Fe(III)-corrole have also demonstrated utility in attenuating Aβ-Cu(II)-mediated ROS.[23] However, the precise therapeutic mechanism remains recondite

[1]Department of Chemistry, the University of Texas at Austin, Austin, TX 78712-1224, USA

[2]Department of Biological Engineering, Massachusetts Institute of Technology, Cambridge, MA 02139, USA

[3]Sir Peter Mansfield Imaging Centre, Division of Clinical Neuroscience, School of Medicine, University of Nottingham, Nottingham NG7 2RD, UK

[4]Department of Chemistry, University of Massachusetts Lowell, Lowell, MA 01854, USA

[5]Department of Chemistry, University of Pavia, Via Taramelli 12, 27100 Pavia, Italy

[6]Accelerated Research Initiative, University of Texas at Austin, Austin, TX 78712, USA

[7]Center for Supramolecular Chemistry and Catalysis, Shanghai University, Shanghai, China

[8]These authors contributed equally

[9]Lead Contact

*Correspondence:
peter.harvey@nottingham.ac.uk (P.H.),
simone.dellacqua@unipv.it (S.D.),
luigi.casella@unipv.it (L.C.),
sessler@cm.utexas.edu (J.L.S.)

https://doi.org/10.1016/j.chempr.2019.12.016

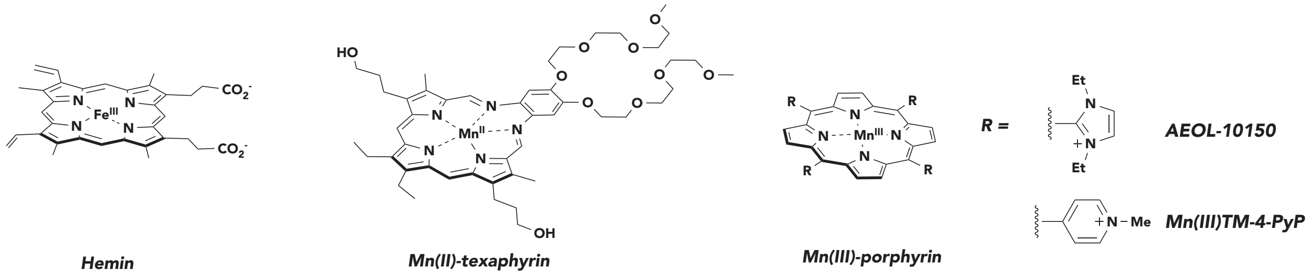

Figure 1. Chemical Structures of Representative Porphyrinoids

As discussed in the text proper, hemin catalyzes oxidative and nitrative damage. Manganese(II) texaphyrin (MMn) is the subject of this study. Manganese(III) tetramethylpyridinium porphyrin (MnTMPyP) is a manganese porphyrin metalloantioxidant. AEOL-10150 has successfully completed a phase I trial in ALS and healthy patients and has shown efficacy in animal models for radiation- and nerve-agent-induced toxicity. Axial ligands are omitted for clarity. See main text for further discussion of these agents.

as Fe(III)- and Mn(III)-porphyrin and corrole complexes also catalyze oxidative transformations (e.g., peroxidase activity).[27,28] Limited cellular uptake and acute toxicity in the case of polycationic pyridinium Mn porphyrins are also an issue.

The interaction of hemin and other water-soluble phthalocyanines, corroles, and porphyrins with Aβ has been noted.[29–32] We thus envisaged that coupling the Aβ-recognition properties of metalloporphyrins with the biological activity of metallotexaphyrins would allow for MRI recognition of Aβ species with attendant utility as a potential metalloantioxidant therapeutic (Figure 2). As detailed further below, we have found that metallotexaphyrins, in particular MMn, interact with amyloid constructs, as inferred from ultraviolet-visible (UV-vis), fluorescence, and circular dichroism (CD) spectroscopies, as well as MRI studies. The protective effect of MMn against oxidative and nitrative damage was also demonstrated by means of *in vitro* studies with Aβ peptides and serotonin as model protein and small-molecule neurotransmitter substrates, respectively, and in cell culture with Neuro-2A cells. The protective and oxidative properties of Mn(III) tetramethylpyridinium porphyrin (MnTMPyP), a gold-standard Mn porphyrin metalloantioxidant, were also studied *in vitro* and in Neuro-2A cell cultures. These studies revealed rapid decomposition of MnTMPyP under oxidative conditions consistent with a sacrificial substrate-based antioxidant mechanism. Studies involving *C. elegans* models were also carried out with MMn and provide support for the notion that this first-generation metallotexaphyrin may be administered and detected in amyloid plaque-bearing AD models. It and related systems could thus have a role to play in the study and eventual management of neurodegenerative disease.

RESULTS AND DISCUSSION

Spectroscopic Analyses of Metallotexaphyrin-Aβ Interactions

Initial studies of the interactions between MMn and $A\beta_{40}$ involved UV-vis spectroscopic analyses. This technique was chosen because changes in the intensity or wavelength (energy) of the Soret band (π-to-π^* transition) of metalloporphyrinoids are often indicative of axial coordination.[33] Reduction in the molar absorptivity can also arise from coordination within a peptide or protein tertiary structure.[34] MMn, like other porphyrinoids, is intensely colored. It is dark green in most media in which it is soluble and characterized by strong Soret- and Q-like bands at λ_{max} = 459 and 726 nm, respectively, in pH 7.4 phosphate-buffered saline (PBS). The addition of monomeric $A\beta_{40}$ (5 μM, 1 equiv) into a PBS solution of MMn (5 μM) resulted in a decrease in spectral intensity for the Soret-like band (ε = 73,700 → 62,500 $M^{-1}cm^{-1}$;

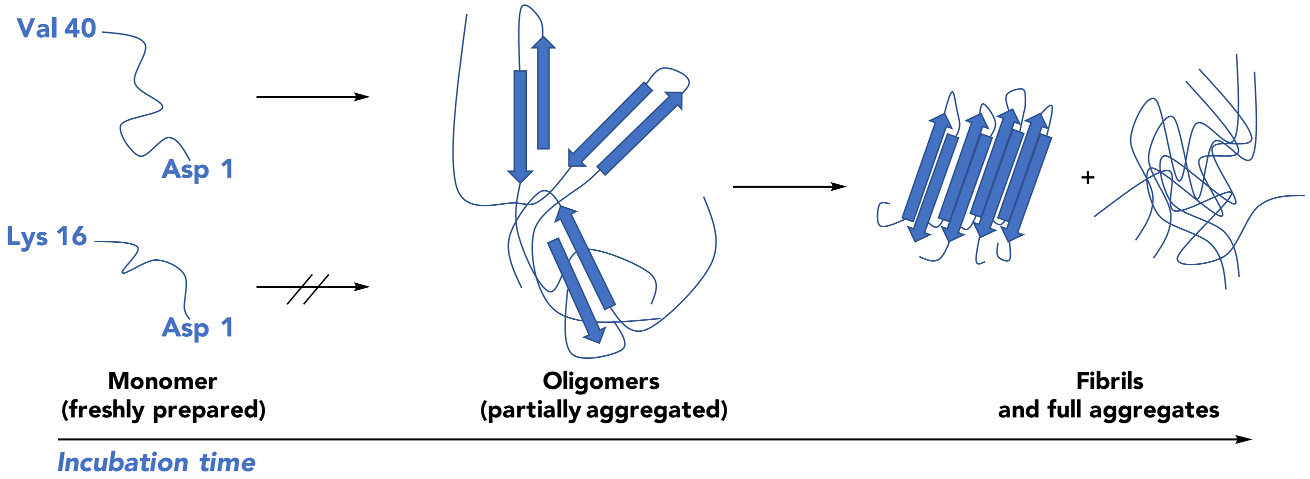

Figure 2. The Aggregation State of Aβ Peptides as a Function of Incubation Time

The three $A\beta_{40}$ constructs utilized in this study: (left) freshly dissolved peptide is predominantly monomeric in solution, (middle) soluble oligomeric species (partially aggregated) are produced after incubation in solution, and (right) fibrils and fully soluble and insoluble aggregates are formed after prolonged incubation. $A\beta_{16}$ remains monomeric in solution.

λ_{max} = 459 nm) (Figure S4). Increasing the ratio of $A\beta_{40}$ led to an enhancement in the molar absorptivity (ε = 72,450 $M^{-1}cm^{-1}$ at 20 equiv) with a slight spectral shift in the absorption maximum also being seen (λ_{max} = 462 nm; Figure 3). UV-vis spectral analyses provided support for the conclusion that the $A\beta_{40}$ peptide (ε = 1,480 $M^{-1}cm^{-1}$ at 280 nm) remains soluble upon addition to MMn. Specifically, no sign of precipitation was observed, even when tested at high concentrations of $A\beta_{40}$ (i.e., 100 μM; Figure S5). A titration of MMn with human serum albumin (HSA) showed a similar reduction in absorbance intensity but yielded no shift in the Soret or Q bands (Figure S6). Considered in tandem, the changes to the UV-vis spectral features of MMn observed in the presence of monomeric $A\beta_{40}$ are consistent with the coordination of one or more axial ligands to the texaphyrin metal center. However, the potential formation of a soluble, higher-order $A\beta_{40}$ species (e.g., a dimeric-MMn complex) in solution cannot be excluded. Under these same conditions, a titration of MnTMPyP, a commercially available porphyrin considered as a gold-standard metalloantioxidant, with $A\beta_{40}$ yielded no change in the observed spectrum (Figure S7). Similar spectral changes to the texaphyrin profile were, however, seen when $A\beta_{40}$ was added to MGd (Figure S7). The marked differences between MMn (and MGd) and MnTMPyP when exposed to $A\beta_{40}$ are believed to arise from differences in the structure and charge of the overall complex. MnTMPyP is decorated with four pyridinium functionalities positioned perpendicular to the porphyrin core, whereas MMn is formally monocationic, planar, and functionalized with flexible pendant substituents capable of hydrogen bonding.

Since $A\beta_{16}$ remains monomeric in solution and conserves the relevant amino acids considered responsible for Aβ coordination chemistry, an analogous titration was carried out between MMn and $A\beta_{16}$ (0–80 equiv) in an effort to differentiate between the effects of aggregation (hydrophobic interactions) and axial coordination on the observed UV-vis spectral changes. UV-vis spectra of MMn recorded as it was exposed to $A\beta_{16}$ were characterized by an initial reduction in spectral intensity, just as was observed in the corresponding $A\beta_{40}$ studies. This decrease was followed by a corresponding increase in spectral intensity. In analogy to what was seen in the case of monomeric $A\beta_{40}$, approximately 17.5 equiv of $A\beta_{16}$ (as opposed to 15 equiv

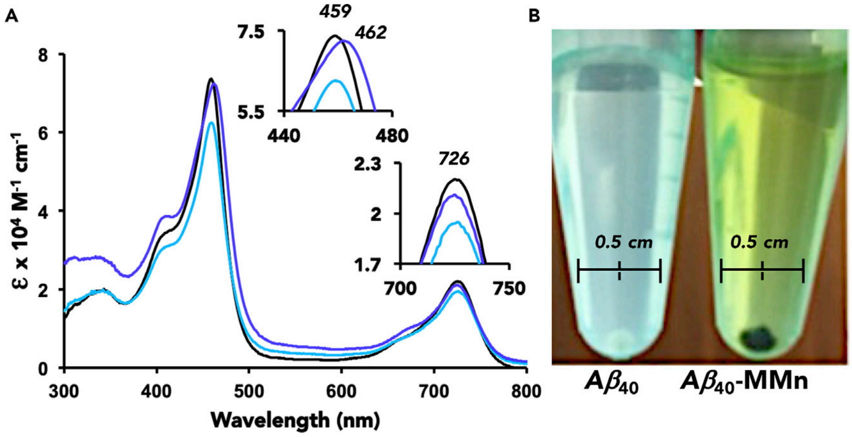

Figure 3. Interaction of MMn with Monomeric and Aggregated Aβ_{40}

(A) Titration with MMn (5 μM) and monomeric Aβ_{40} with 0 (black), 1 (light blue), and 20 (purple) equiv and (B) photos of aggregated Aβ_{40} (100 μM; aged 2 months) in the absence and presence of MMn (100 μM) in PBS and after washing with PBS. The pellet shown in the right-most image was obtained by centrifugation and is in methanol.

for Aβ_{40}) were required to reach an absorbance plateau. Subsequently, no further discernible increase or change in the Soret band (λ_{max}) was observed (i.e., upon addition of >20 equiv Aβ_{16}; cf. Figure S8). The spectral changes induced by Aβ_{16} and monomeric Aβ_{40} were thus attributed to axial ligand binding rather than sequestration by oligomeric or aggregated peptide species.

MMn was also titrated with Aβ_{40} (0–20 equiv) that was pre-incubated for 14 days in PBS at room temperature. This pre-incubated material is believed to contain higher-order oligomeric species (i.e., partial aggregates containing β sheets; cf. Figure 2). Treating MMn with oligomeric Aβ_{40} (1 equiv) engenders a decrease in the UV-vis spectral intensity (ε = 73,700 → ε = 45,600 $M^{-1}cm^{-1}$), a finding attributed to both axial ligand binding and incorporation of MMn within the aggregate structure. Upon adding additional oligomeric Aβ_{40} (up to 20 equiv), an increase in spectral intensity (ε = 50,000 $M^{-1}cm^{-1}$) and a bathochromic shift in the Soret band maximum (to λ_{max} = 462 nm) were observed (Figure S9). Analyses of the relative spectral intensity of the Soret and Q bands (ratio of absorbance at 459 and 726 nm) revealed a minute change in this ratio for aggregated (3.39:1) and monomeric (3.43:1) forms of Aβ_{40}. This was taken as further support for the suggestion that the UV-vis spectral changes seen for MMn upon exposure to Aβ_{40} originate from both axial coordination to the Mn(II) center and texaphyrin encapsulation within Aβ_{40} oligomers, fibrils, and aggregates.

To gain insights into the interactions between MMn and fully aggregated Aβ species, we mixed constructs composed of Aβ_{40} that had been aged for 2 months (a highly aggregated form) with MMn (100 μM, 1 equiv). After centrifugation, a green pellet was obtained. Even after washing and vortex mixing with deionized (DI) water (three times) and resuspension in methanol, the pellet remained green (Figure 3). A time-dependent UV-vis analysis of the spectral changes seen for MMn when exposed to this same aggregated Aβ_{40} species was carried out by following the decrease in the absorbance intensity of the Soret band (λ = 459 nm) as a function of time. Upon addition, an immediate decrease was observed. A further reduction in the absorbance intensity was then seen over the ensuing 30 min (Figure S10).

A competition experiment involving HSA and monomeric Aβ_{40} was also carried out in an effort to discriminate between selectivity for Aβ_{40} versus general biomolecule interactions. The first addition of Aβ_{40} (25 μM) to a preformed MMn (5 μM)-HSA (5.5 μM) complex induced a slight decrease in the absorption intensity of the Soret (5.8%) and Q (6.3%) bands. Subsequent addition of Aβ_{40} (25 μM) again resulted in a further decrease in the absorption intensity of the Soret (4.6%; 10.4% total) and Q (6.8%; 13.1% total) bands with concomitant bathochromic shifts in the Soret band from 459 to 460 nm and Q band from 726 to 728 nm (Figure S11). Increasing the concentration of Aβ_{40} (125 μM) and HSA (600 μM) yielded a green pellet by centrifugation in the presence and absence of HSA after the induction of Aβ fibrillization through storage at 4°C for 48 h (Figure S12).[35] However, the MMn-Aβ_{40} pellet formed in the presence of both HSA and Aβ_{40} was smaller (ca. 0.2 cm) than that formed when MMn was treated with just Aβ_{40} (ca. 0.35 cm). No pellet was formed from MMn in the absence of Aβ_{40}.

Further attempts to probe the interactions between MMn and oligomeric Aβ were made with an Aβ_{40} (aged 1 week) Thioflavin T (ThT) assay.[36] The benzothiazole scaffold for ThT is a recognized binding agent for Aβ sequences conserved among many PET agents (e.g., the FDA-approved Vizamyl [flutemetamol (^{18}F)]) and theranostics used in AD research.[37] New small molecules with binding affinities similar to that of ThT could thus prove useful for clinical application.[30] As important, the ThT assay can provide evidence of binding within Aβ_{40} because of the well-defined ThT-Aβ_{40} emission profile.[36] Changes in the observed fluorescence correspond to ThT displacement or structural modification of the Aβ_{40} aggregate. To exploit this assay, we added MMn (1 to 10 equiv) in aliquots to Aβ_{40} (aged 1 week) containing ThT (ThT-Aβ_{40} constructs). These additions engendered a series of changes, including spectral shifts and an overall decrease in the intensity (Figure S13). Through-space quenching of bound or free MMn, as a result of the matched absorption spectrum of MMn and emission profile of ThT-Aβ_{40} constructs, accounts for some of the initial decrease (Figures S14 and S15). Continued modification of the fluorescence profile was seen upon the addition of each aliquot of MMn, a finding consistent with the presence of multiple binding sites within the Aβ_{40} aggregate. The observed modifications to the fluorescence profile are thus largely attributed MMn-induced structural reorganization that is triggered by binding to aggregated Aβ_{40}.

We carried out high-performance liquid chromatography (HPLC) analyses in an effort to elucidate further the interactions between ThT (100 μM), MMn (100 μM), or MLu (100 μM) and 2-month-aged Aβ_{40} aggregates (100 μM) (Figure S16). After incubation at room temperature for 24 h, 77% ThT remained in the supernatant, as determined by integration against a ThT control (100 μM). Co-incubation with MMn or MLu (24 h at room temperature) effected little significant displacement given that 66.7% ThT and 7% MMn remained in the supernatant, as determined by HPLC analysis using ThT and MMn standards. A similar HPLC study using the diamagnetic MLu revealed that only 5.4% of the MLu remained in the supernatant (Figure S17). NMR spectroscopic experiments carried out with preformed ThT-Aβ_{40} samples (ThT [0.47 mM] and Aβ_{40} [0.40 mM]) in conjunction with MLu (0.49 mM) revealed signs of ThT displacement after incubation for 24 h at room temperature (Figures S18 and S19). Additional NMR spectroscopic analyses using monomeric Aβ_{40} (1.9 mM) and MLu (2.1 mM) in DMSO-d_6 revealed changes to the proton signals ascribed to MLu and the Aβ_{40} peptide. The most notable difference after the addition of Aβ_{40} was a change in the chemical shift for the axial acetate anions originally bound to MLu; this is as would be expected under a scenario where the

A

B

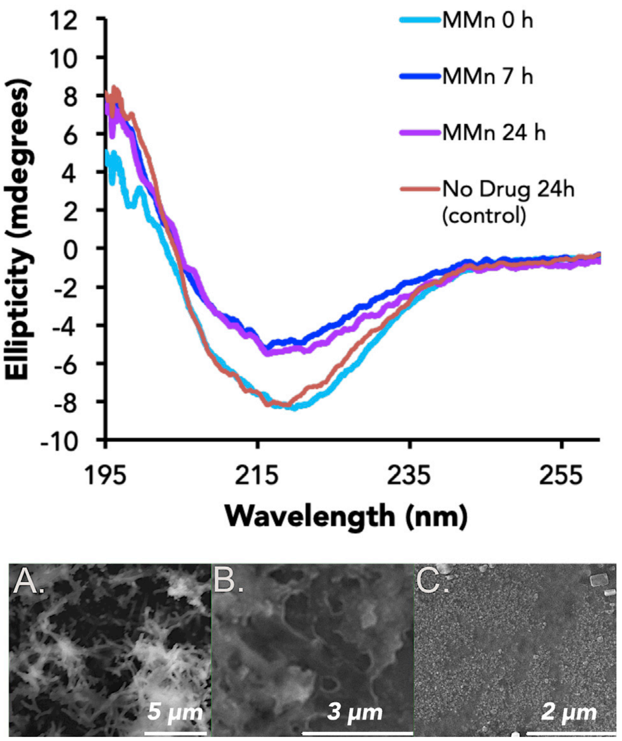

Figure 4. Modification of Aggregated $A\beta_{40}$ by MMn as Monitored by CD Spectroscopy and SEM Analyses

CD spectra (left) of aggregated (aged for 30 days) $A\beta_{40}$ (40 μM) were recorded before and after the addition of MMn (20 μM) at t = 0, 7, and 24 h. SEM photographs (right) of (A) $A\beta_{40}$ aggregate, (B) an $A\beta_{40}$ aggregate incubated with MMn for 24 h, and (C) monomeric $A\beta_{40}$ are shown.

Aβ coordinates the metallotexaphyrin (Figure S20). In concert, these findings are taken as evidence that metallotexaphyrins bind well to aggregated Aβ, most likely through multiple binding sites, even if the specifics of the interaction are not fully elucidated.

Clinical trials aimed at the modification of higher-order oligomeric and aggregated Aβ have yet to yield viable therapeutics.[11] An ability to produce and detect such modifications remains of interest in terms of assessing the potential impact of other treatment modalities. CD studies designed to assess the ability of MMn (20 μM) to interact with and modify the aggregate structure of Aβ (20 μM, aged 30 days) revealed no marked differences in the CD spectrum immediately after mixing. However, noticeable changes relative to the initial CD spectrum were observed 7 and 24 h after mixing (Figure 4). In particular, the intensity of the negative CD band at 220 nm seen for aggregated $A\beta_{40}$ underwent a decrease in intensity of approximately 35% at 24 h with an attendant shift in the wavelength minimum to ca. 218 nm. These data are interpreted in terms of a reduction in the percentage of β sheet composition and modification of the overall aggregate structure.[38] Control studies monitoring the progression over 24 h with aged $A\beta_{40}$ in the absence of MMn revealed no significant change (Figure 4).

Scanning electron microscopy (SEM) provided further support for the proposed modification of the $A\beta_{40}$ aggregate structure (Figure 4). The $A\beta_{40}$ aggregate (40 μM) utilized for CD experiments (aged 30 days plus 24 h) was characterized by

fibril structures, observed at 5 μm (Figure 4A). In contrast, the $A\beta_{40}$ aggregate (aged 30 days) incubated with MMn (20 μM) for 24 h displayed features at 3 μm more closely resembling those of an amorphous species (Figure 4B). Freshly prepared $A\beta_{40}$ showed no discrete features even when magnified to 2 μm (Figure 4C).

In Vitro and *In Vivo* MRI

As a general rule, paramagnetic Gd(III) and high-spin Mn(II) ions enhance the relaxation rates of nearby proton nuclei. Not surprisingly, these cations have been incorporated into MRI contrast agents (CAs).[39] Gd-based systems remain the current clinical standard. However, Mn-based CAs have recently been explored as surrogates for Gd systems and as new intramolecular MRI probes.[40,41] Detailed MRI studies on MGd have previously been reported,[42] including human trials where evidence of tumor-targeted imaging via enhanced drug uptake within tumor cells was obtained.[18,19] However, the congeneric Mn(II) system MMn has yet to be the subject of extensive study in the context of MRI.[43,44]

MRI CAs are generally described by relaxivities, r_1 and r_2, in $mM^{-1}\ s^{-1}$. These parameters are defined as the concentration-dependent enhancement of longitudinal (R_1) and transverse (R_2) water proton relaxation rates, each given in s^{-1}, respectively. At the field used in this study (7 T), MMn displayed exceptional relaxivities in non-coordinating buffers (7 T, HEPES [pH 7.4], 25°C; MMn $r_1 = 4.59 \pm 0.12\ mM^{-1}\ s^{-1}$ and $r_2 = 32.2 \pm 0.3\ mM^{-1}\ s^{-1}$; MGd $r_1 = 16.0 \pm 0.5\ mM^{-1}\ s^{-1}$ and $r_2 = 23.5 \pm 0.6\ mM^{-1}\ s^{-1}$; Gd-DTPA $r_1 = 4.93 \pm 0.06\ mM^{-1}\ s^{-1}$ and $r_2 = 6.65 \pm 0.02\ mM^{-1}\ s^{-1}$) with well-behaved (i.e., linear) relaxation rates as a function of concentration. The longitudinal relaxivity of MMn in the coordinating buffer (i.e., PBS) was slightly reduced at $r_1 = 1.2\ mM^{-1}\ s^{-1}$. Current CAs are known to be limited at these higher fields and previous examples of Aβ-binding MRI agents have, in general, been investigated at a lower magnetic-field strength.[39,40,45] The ability of MMn to engender high relaxivities at a higher field could make such agents of clinical interest in the context of neurodegenerative disease. Studies with Aβ peptides were carried out as a first step toward exploring this potential.

Changes in the relaxation rates (R_1 and R_2) after addition of monomeric and aggregated $A\beta_{40}$ (70 μM) to solutions of MMn (70 or 140 μM) at 25°C in PBS (pH 7.4) were monitored at 7 T with a multi-spin multi-echo scanning sequence. Bovine serum albumin (BSA, 70 μM) was used as a control biomolecule (Figure 5). All of the additives tested gave rise to an increase in the longitudinal relaxation rates of MMn. Control studies demonstrated that $A\beta_{40}$ alone displayed no significant effect on the relaxation rate of the buffer ($R_1 = 0.400 \pm 0.005\ s^{-1}$ versus the PBS control $R_1 = 0.398 \pm 0.005\ s^{-1}$). Relative to the relaxation value generated by MMn alone, aggregated $A\beta_{40}$ induced the largest increase in R_1 (Figure 5A). The modest increase observed for monomeric $A\beta_{40}$ and albumin coupled with the observed enhancement in the R_1 parameter in the case of aggregated Aβ is taken as a further indication that such agents could find utility in the MRI-based detection of Aβ aggregate constructs. Interestingly, with the fully aggregated $A\beta_{40}$, the R_1 increase was substantially larger for 2 equiv of MMn (ca. 62% versus MMn alone) than for 1:1 MMn/$A\beta_{40}$ (ca. 37% versus MMn alone) (Figure S21). Considered in light of the ThT-$A\beta_{40}$ fluorescence assay results described above, these data are taken as a further indication that MMn binds to multiple sites within aggregated $A\beta_{40}$. The MRI properties of Gd(III) texaphyrin (MGd, 70 μM) in the presence of aggregated $A\beta_{28}$ (500 μM) and BSA (600 μM) were also monitored at 7 T with a multi-spin multi-echo scanning sequence at 25°C in PBS (pH 7.4). However, these studies failed to show selectivity or significant changes in either R_1 or R_2 in either case (Figure S22).

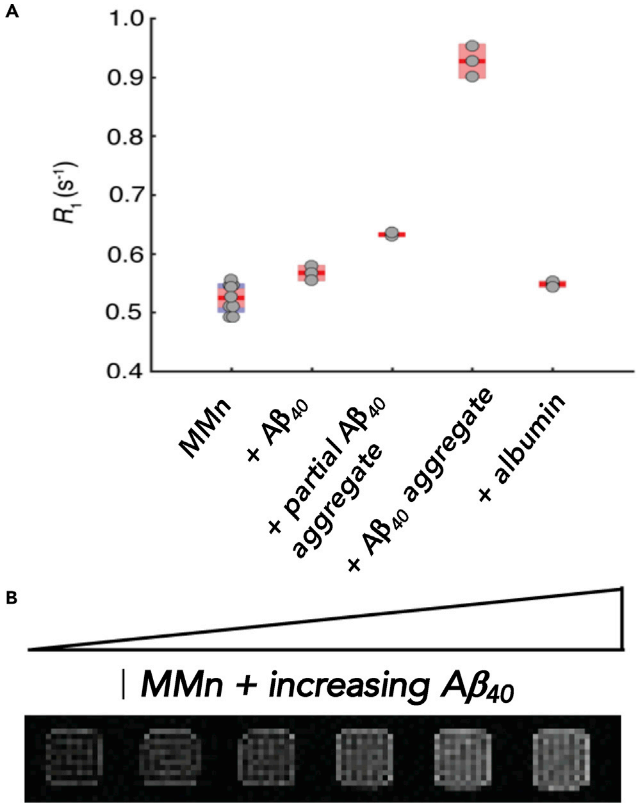

Figure 5. *In Vitro* MRI Analyses of MMn with Various Aβ_{40} Constructs

(A) The longitudinal relaxation rate (R_1) of MMn (140 μM) with monomeric (0.5 equiv, 70 μM), partial (1 week, 0.5 equiv, 70 μM), and aggregated (>2 months) Aβ_{40} (0.5 equiv, 70 μM) in comparison with MMn alone (140 μM) and in the presence of BSA (0.5 equiv, 70 μM). Dots represent individual samples; the mean (red line) and 95% confidence level for the mean (light red) are also displayed. Standard deviations are either indicated in blue or not shown if within the 95% confidence level.

(B) Phantom T1-weighted MRI images of 140 μM MMn were recorded in the presence of increasing concentrations of Aβ_{40} aggregate (0–70 μM) in PBS at 7 T and 25°C. Samples are normalized to MMn alone, shown in the left-most box.

Access to the intracellular intrinsically disordered proteins and peptides (IDPs) remains difficult for most MRI CAs and often comes at the cost of desirable physicochemical properties (e.g., LogP and solubility).[16,40,41] Cell-uptake studies using MMn (30 μM), LogP 0.69, and the Neuro-2A cell line with a 9 h incubation period produced a green cell pellet with a 5.5-fold increase in the Mn content (2.2 ppb per million cells) versus the no-drug control (0.4 ppb per million cells), as quantified by ICP-MS analysis (Figure S23). MRI of the resultant cell pellet suspended in PBS provided additional support for the proposed increase in uptake given that both R_1 (from 0.382 ± 0.004 to 0.412 ± 0.004 s^{-1}) and R_2 (from 2.43 ± 0.03 to 2.71 ± 0.03 s^{-1}) were enhanced in comparison with the no-MMn control.

In order to probe the possible utility of MMn for *in vivo* MRI-based detection of neurodegenerative disease, we incubated an AD model of *C. elegans* (CL4176) expressing amyloid plaques in the presence of MMn (1 mL/100 μM).[46] The resulting MMn content, measured by MRI and ICP-MS, was compared to what was seen in the case of the wild-type strain, N2. On the basis of reported drug absorption and retention within *C. elegans*, we envisaged that MMn would interact and be retained within the amyloid plaques, yielding higher MRI and ICP-MS signals than the

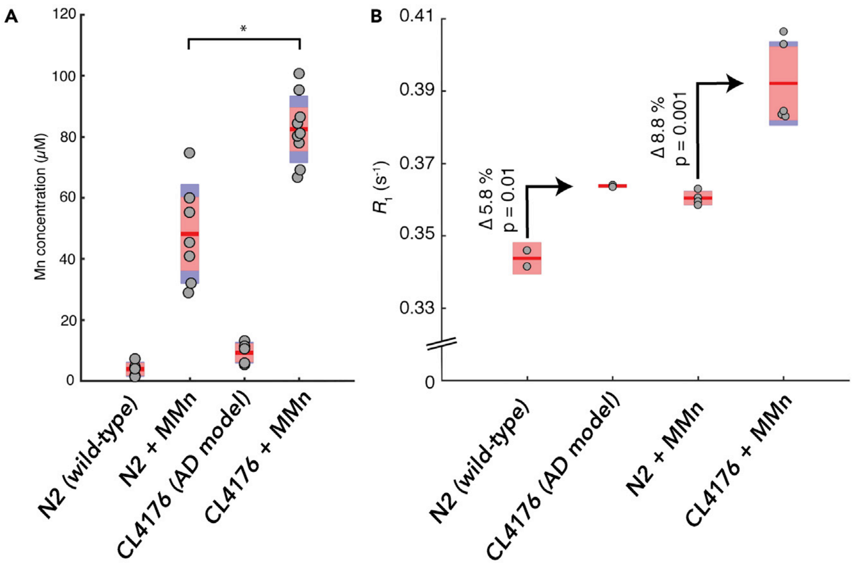

Figure 6. ICP-MS and *In Vivo* MRI Analyses of MMn in *C. elegans* AD Models

Mn concentrations (A), as determined by ICP-MS, and (B) MRI analyses of MMn for pellets of wild-type (N2) and AD model (CL4176) *C. elegans* with and without MMn. Pellets were standardized by dissolving every 1 mg *C. elegans* in 100 μL HEPES and digesting in HNO_3. n = 5–8 per sample, 2 replicates of each for ICP-MS measurements and n = 2–5 for MRI measurements. Dots represent individual samples; the mean (red line) and 95% confidence level for the mean (light red) are shown. Standard deviation (blue) is shown where larger than the 95% confidence level. *Two-sample t test on ICP-MS variance between MMn-wild-type (WT) and -AD models; p = 0.00018.

control.[47] We also used extended incubation times to allow for multiple uptake and excretion cycles. Control experiments using *E. coli* OP50 with MMn and worms without MMn were carried out concurrently with the experiments involving the N2 and CL4176 strains (Figure S24). MRI studies on the resulting pellets suspended in HEPES buffer (10 mM [pH 7.4], 1 mg pellet per 100 μL HEPES) showed an enhanced R_1 signal in the CL4176 AD model (0.394 ± 0.012 s^{-1}) when they were administered MMn versus the N2 treated with MMn in an equivalent way (0.360 ± 0.002 s^{-1}), as well as no-MMn CL4176 (0.364 ± 0.003 s^{-1}), N2 (0.344 ± 0.003 s^{-1}), and *E. coli* with MMn control studies (0.333 ± 0.002 s^{-1}). A similar trend was also seen in the case of the R_2 with relevant values: CL4176 AD model with MMn (3.44 ± 0.20 s^{-1}), N2 with MMn (2.82 ± 0.03 s^{-1}), no-drug CL4176 (3.14 ± 0.02 s^{-1}), no-drug N2 (2.59 ± 0.10 s^{-1}), and *E. coli* with MMn control (2.450 ± 0.04 s^{-1}) (Figure S25). A second batch displayed a similar trend (Figure S26).

As expected, both batches of *C. elegans* incubated with MMn showed higher Mn signal in the N2 and CL4176 strains than did the same strain without the texaphyrin (Figure 6). ICP-MS analyses of pellet suspensions (1 mg *C. elegans* per 100 μL HEPES) revealed Mn uptake values of 82 ± 11 μM (SD) for the CL4176 AD model and 48 ± 11 μM for the wild-type N2 (Figure S27). The increased metal uptake in the AD model is taken as evidence of the accumulation of MMn within the amyloid plaque over multiple uptake-drug-excretion cycles.

In Vitro ROS and RNS Reactivity Studies

Nitrative and oxidative damage is prevalent in AD brains.[48] Oxidized and nitrated neurotransmitters, DNA bases, proteins, metalloenzymes, and cellular membrane components are also correlated with disease progression. These chemical changes are thought to alter up- and downstream biochemical processes via inactivation,

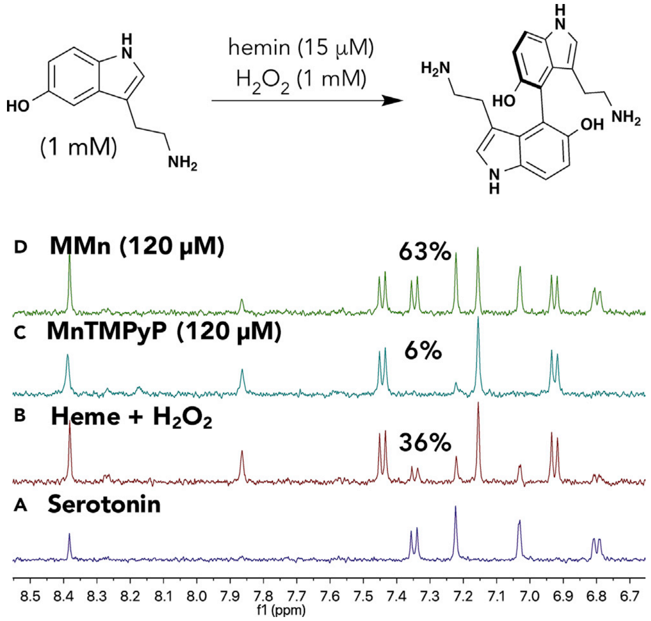

Figure 7. ^{1}H NMR Spectroscopic Study of the Hemin-Mediated Oxidation of Serotonin

NMR yields were calculated by comparing the spectra of (A) serotonin (1 mM) in pH 7.4 PBS at 37°C in the presence of (B) hemin (15 μM) and an oxidative mixture containing H_2O_2 (1 mM) and sodium ascorbate (40 μM), (C) an H_2O_2-ascorbate oxidative mixture plus MnTMPyP (120 μM), and (D) an H_2O_2-ascorbate oxidative mixture plus MMn (120 μM) recorded after a reaction time of 120 min in all cases.

hyperactivation, and off-target effects.[49,50] Recent studies have thus led to the suggestion that oxidation and nitration of the amyloid peptide and tau proteins leads to enhanced cytotoxicity with attendant neuronal cell death.[51,52] MMn is known to display distinct redox behavior in comparison with its related Mn(III)porphyrin and corrole congeners.[53] Previous work on MMn has also illustrated the possibility of deactivating $ONOO^-$ catalytically in the presence of sodium ascorbate (NaAsc) with attendant application in preventing formation of nitrotyrosine in ALS mice models.[54,55] The propensity of metallotexaphyrins to interact with Aβ constructs coupled with the unique redox capabilities of MMn led us to explore whether this specific metallotexaphyrin could be used to mitigate the effects of hemin-mediated oxidation and nitration and copper-mediated oxidation of small-molecule neurotransmitters and amyloid peptides. The pro-oxidative and protective (antioxidant) activity of MnTMPyP has also previously been reported.[24,56] It was thus studied for comparative purposes.

Serotonin was used as the first test substrate. When hemin is activated in the presence of hydrogen peroxide, serotonin undergoes an oxidative transformation to yield the neurotoxic 4,4′-serotonin dimer (5,5′-dihydroxy-4,4′-bitryptamine [DHBT]). The antioxidant (protective) effect of MMn was studied with serotonin (1 mM) with hemin (15 μM) and an oxidative mixture consisting of hydrogen peroxide (1 mM) and NaAsc (40 μM) in PBS (pH 7.4) at 37°C for 2 h. By comparing the changes observed in the corresponding ^{1}H NMR spectra in D_2O, we could compare the antioxidant effect of MMn against that of MnTMPyP, a well-studied Mn(III)-porphyrin metalloantioxidant, and the pro-oxidative hemin control (Figure 7). Under these conditions, MMn proved the most protective, leaving behind 63% of unmodified serotonin. In contrast, the hemin/H_2O_2/ascorbate and hemin/H_2O_2/ascorbate/MnTMPyP mixtures showed a marked increase in serotonin oxidation with 36%

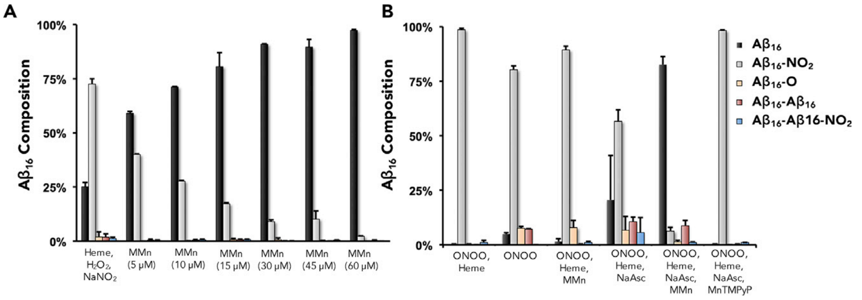

Figure 8. HPLC-MS Analyses of the Oxidative and Nitrative Modification of Aβ$_{16}$

(A) Aβ$_{16}$ incubated under hemin-nitration conditions using heme-H_2O_2-$NaNO_2$ with varying concentrations of MMn. Percent composition of Aβ$_{16}$ is shown in black and, as expected, increases with the [MMn].

(B) The protective effect of MMn was also tested against heme-peroxynitrite-mediated modification of Aβ$_{16}$ in comparison with MnTMPyP. Sodium ascorbate (NaAsc) was added to regenerate the MMn catalyst under the reaction conditions. Reported values are averages with the corresponding standard deviation as obtained from two independent measurements.

and 4% remaining serotonin, respectively. An analogous experiment using hemin (15 μM) and H_2O_2 (1 mM) without NaAsc revealed that MMn was protective, yielding 70% of unmodified serotonin versus 60% and 40% remaining for hemin/H_2O_2 and hemin/H_2O_2/MnTMPyP, respectively (Figure S28). Additional NMR spectroscopic experiments designed to evaluate the protective effect of MMn against Cu(II), another metal ion considered relevant to oxidation in CNS disorders, were also carried out (Figure S28). Qualitative ^{1}H NMR spectroscopic analyses using Cu(II)Cl_2 (100 μM), serotonin (1 mM), and H_2O_2 (1 mM) without and with MMn (120 μM) revealed that prolonged incubation times and high concentrations of the metal salt were necessary for producing low levels of oxidation. Shorter reaction times and lower concentrations of Cu(II) led to no detectable oxidation, as inferred from ^{1}H NMR spectral studies.

MMn also proved efficient at deactivating the oxidative modification of histidine (H13-H14), nitration of tyrosine (Y10), and oxidative dimerization of tyrosine (Y10) in Aβ$_{16}$ by the more reactive hemin, as determined by high-performance liquid chromatography-tandem mass spectrometry (HPLC-MS/MS) analysis. The Aβ$_{16}$ sequence serves as a soluble surrogate for larger amyloid species or other proteins that could suffer degradation when treated with the oxidative mixture of hemin-hydrogen peroxide (15 μM-1 mM) or copper(II) (15, 60, or 150 μM) with hydrogen peroxide (1 mM) or nitrative mixtures of hemin-hydrogen peroxide-sodium nitrite (15 μM-1 mM-1 mM) and hemin-$ONOO^-$ (15 μM-1 mM) at 37°C in phosphate buffer (pH 7.4, 5 mM). A range of reaction variables—such as the use of acidic pH thought relevant to neurodegeneration,[57] solution ionic strength (5 versus 50 mM), reaction time (2–24 h), and the presence or absence of MnTMPyP, MGd, and curcumin—were also studied in the context of hemin-mediated oxidation and nitration of Aβ$_{16}$ (Figures S29–S32). Within the range of a normal physiological pH of 7.4 in low-ionic-strength 5 mM phosphate buffer, only 25.1% ± 2.1% (SD) of the Aβ$_{16}$ remained in the case of the additive-free control, composed of Aβ$_{16}$ (300 μM), hemin (15 μM), H_2O_2 (1 mM), and $NaNO_2$ (1 mM). MMn was found to militate against oxidation and nitration of Aβ$_{16}$ with an IC_{50} = 4.22 μM and a maximal inhibition of 97.4% ± 0.4% at 60 μM MMn under nitrative conditions (Figure 8A). A similar protective effect by MMn was also seen under oxidative conditions of Aβ$_{16}$ (300 μM), hemin (15 μM), H_2O_2 (1 mM), and MMn (30 μM), yielding 98% ± 0.4% of unchanged Aβ$_{16}$. Control experiments without MMn, with MGd, and with the sacrificial antioxidant curcumin

gave rise to 83.5% $\pm$ 5% (Δ = $-$13.9%), 77.5% $\pm$ 5.5% (Δ = $-$19.9%), and 84.5% $\pm$ 2% (Δ = $-$12.9%) of unchanged Aβ_{16}, respectively. At a more acidic pH of 6.3 and the higher ionic strength (i.e., 50 mM phosphate buffer), under nitrative conditions, the amount of remaining Aβ_{16} (300 μM) decreased from 63.5% $\pm$ 1.4% at pH 7.4 to 33.3% at pH 6.3 (heme-H_2O_2-$NaNO_2$; 10 μM-1 mM-1 mM). However, the protective effect of MMn (30 μM) was enhanced such that 98.8% $\pm$ 0.6% of the Aβ_{16} remained intact. This increased benefit is believed to arise from deactivation rates that are accelerated in more acidic media. The results of the Cu(II)-H_2O_2 experiments proved difficult to quantify. This difficulty reflects the minimal oxidative reactivity of Cu(II) at levels appropriate for HPLC-MS analysis (Figure S33). Under conditions analogous to those tested in the case of hemin, Cu(II) (15 μM) with H_2O_2 (1 mM) incubated at 37°C for 2 h in phosphate buffer (pH 7.4, 5 mM) returned 96.9% $\pm$ 1.7% of unchanged Aβ_{16}. The addition of MMn (30 μM) to Aβ_{16} (300 μM), Cu(II) (15 μM), and H_2O_2 (1 mM) yielded 98.7% $\pm$ 0.4% unchanged Aβ_{16}.

It is worth stressing that although it promotes the oxidation of serotonin, the gold-standard control system, MnTMPyP, was found to be protective against the oxidation and nitration of Aβ_{16}. In particular, MnTMPyP showed an excellent protective effect against oxidative modification by yielding 97.4% $\pm$ 0.6% unmodified Aβ_{16}. MnTMPyP also proved protective against nitrative modification by yielding 88.7% $\pm$ 0.6% unmodified Aβ_{16} (Figure S34). These are both similar to the values observed for MMn (98% $\pm$ 0.4% for oxidation and 91% $\pm$ 0.1% for nitration). The observed protective effect of MnTMPyP is thought to arise from rapid oxidative degradation of hemin and MnTMPyP via the iron- and Mn-oxo porphyrin intermediates. This loss in complex arising from MnTMPyP serving as a sacrificial antioxidant would, in turn, we propose, preclude extensive oxidation or nitration of this amyloid peptide (i.e., Aβ_{16}) during the time course of the 2 h reactivity study. UV-vis analyses provided further support for the notion that MnTMPyP (5 μM) serves as a sacrificial substrate in the presence of hemin (15 μM) and H_2O_2 (1 mM) given that only 13% MnTMPyP and 60% hemin remain after incubation for 10 min at 37°C in phosphate buffer (pH 7.4) (Figure S35). A control experiment with hemin alone under the same conditions yielded 56% unmodified hemin.

Reactivity studies designed to test the protective effect against $ONOO^-$-mediated damage of Aβ16 were also carried out. Here, a mixture of hemin (15 μM), Aβ_{16} (300 μM), and $ONOO^-$ (1 mM) were incubated at 37°C for 30 min in phosphate buffer (5 mM, pH 7.4) and yielded 0.2% $\pm$ 0.1% unmodified Aβ_{16}. In the presence of MMn (30 μM) with NaAsc (500 μM) as the reductant, 82.6% $\pm$ 3.7% of the original Aβ_{16} remained. Notably, in the presence of MnTMPyP and NaAsc, only 0.4% Aβ_{16} was observed intact under otherwise identical conditions (Figure 8B). We attribute this lack of protection for MnTMPyP to the rapid oxidative action of $ONOO^-$ and formation of an oxidative high-valent Mn- or Fe-oxo species that could oxidize Aβ_{16} prior to decomposition.[58] According to early mechanistic studies, such oxo species are not thought to be produced when MMn is used to deactivate $ONOO^-$ in the presence of NaAsc.[54] Control experiments with NaAsc (500 μM), MMn (30 μM) alone (not containing a reductant to regenerate the active Mn(II) species), and MGd as a texaphyrin control containing a non-redox-active metal conferred little protection, such that 20.6% $\pm$ 20.4%, 1.6% $\pm$ 1.2%, and 1.6% $\pm$ 0.2% Aβ_{16} remained intact, respectively (Figure S32).

UV-vis spectroscopic studies revealed that upon addition of MMn (1 equiv, 10 μM) to the six-coordinate 2:1 low-spin Aβ_{16}-heme (200-10 μM) complex, formed by the addition of an excess of Aβ_{16} to hemin, a hypsochromic shift in the absorption of

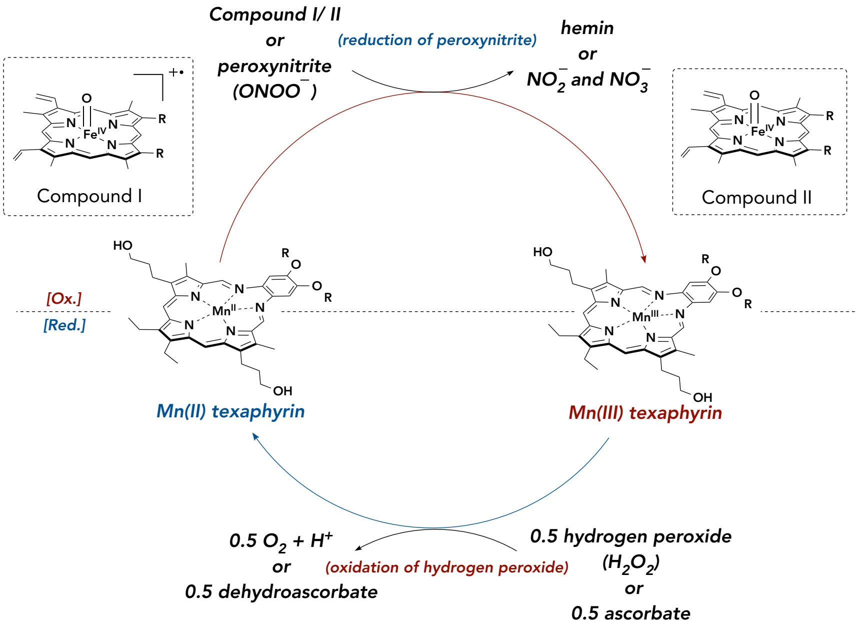

Scheme 1. Proposed Mechanism for the MMn-Based Deactivation of Hemin- and Peroxynitrite ($ONOO^-$)-Mediated Oxidation and Nitration, as well as Oxidative Decomposition of Hydrogen Peroxide (H_2O_2)

Redox cycle of Mn(II/III) texaphyrin. Compounds I and II are generated by the reaction of hemin with H_2O_2. The Aβ peptide and axial ligands are omitted for clarity.

the hemin-derived features from λ_{max} = 412 to 409 nm occurred (Figure S36). Such changes can be ascribed to two possible mechanisms. First, upon addition of MMn, the reversion from 412 to 409 nm in the Soret band could reflect deconstruction of the 2:1 heme-$A\beta_{16}$ construct to yield a mixed low- (2:1) and high-spin (1:1) $A\beta_{16}$-heme species via MMn displacement of the heme bound to $A\beta_{16}$.[59] A separate mechanism could also be operable wherein MMn co-binds within the 2:1 $A\beta_{16}$-heme construct to distrupt the Aβ axial coordination to hemin or modification of the secondary coordination environment responsible for the enhanced peroxidase activity. To such an extent that this latter rationale is correct, it would support a mechanism where MMn-mediated protection results from a proximity effect, as well as an attenuation of the heme peroxidase activity that is normally enhanced upon coordination of hemin to amyloid peptides.[59] Both proposed mechanisms would be expected to induce changes to the UV-vis spectrum of the 2:1 hemin-$A\beta_{16}$ complex.

A proposed mechanism for the deactivation of iron-oxo-mediated oxidation and nitration is shown in Scheme 1. In briefl, the hemin-Aβ complex is activated by H_2O_2 or $ONOO^-$ to yield a highly oxidative porphyrin π-radical cation/iron(IV)-oxo species (compound I in Scheme 1A).[60] The resultant species readily oxidizes hydrogen peroxide or nitrite as well as tyrosine, histidine, or methionine residues in Aβ (producing compound II in Scheme 1). MMn could confer a protective effect by reducing compound I and/or compound II, resulting in the formation of an oxidized Mn(III) texaphyrin. In contrast to Mn peroxidase and certain model Mn complexes, such as Mn(III)TMPyP (*vide infra*), the resulting oxidized Mn(III) texaphyrin fails to carry out additional oxidative chemistry on the peptide or small redox-active molecules. The oxidized Mn(III) texaphyrin can then be reduced by ascorbate or oxidative decomposition of H_2O_2 to O_2 and 2 H^+ (Figure S37).[54] Support for this

H_2O_2-decomposition mechanism comes from UV-vis analyses. Complete reduction of Mn(III) texaphyrin (generated with $ONOO^-$) was observed after the addition of 10 equiv H_2O_2 within 3 min at room temperature in PBS (Figure S38). A control experiment that involved monitoring the reduction of Mn(III) texaphyrin to Mn(II) texaphyrin without H_2O_2 yielded approximately 7% reduced Mn(III) after 20 min when left open to air at room temperature. Under the reaction conditions containing a large excess of H_2O_2 (1 mM) versus MMn (30 μM), it is expected that the H_2O_2 oxidation mechanism will dominate the catalytic cycle to regenerate Mn(II) texaphyrin. No oxidation of Mn(II) texaphyrin to the corresponding Mn(III) species was observed with H_2O_2 alone (Figure S39).

As implied above, these proposed MMn-relevant mechanisms stand in contrast to what is thought to occur with Mn(III)TMPyP. Under oxidizing conditions, such as those employed in this study, the Mn(III) center of MnTMPyP is oxidized to give a Mn(IV)-oxo species that promotes self-decomposition and substrate oxidation. The mixed protective (antioxidant) and oxidative properties of MnTMPyP is thus attributed to variations in the respective redox profile of MnTMPyP versus various biological substrates.[61] Indeed, in instances where the substrate is more prone to oxidative modification (e.g., serotonin or methionine), a pro-oxidative-type mechanism dominates.[55] In cases where the substrate is less labile under oxidative conditions (e.g., histidine and tyrosine) and generation of the oxidative species is slow (i.e., hemin-hydrogen peroxide), MnTMPyP instead acts as an antioxidant engendering a protective effect as a sacrificial substrate. Under more strongly oxidizing conditions, such as hemin-$ONOO^-$, MnTMPyP again fails to convey any protection. The observed toxic effect for MnTMPyP in *in vitro* cell culture could also reflect the release of free Mn as a result of the rapid oxidative decomposition of this particular porphyrin complex seen upon treatment with hemin and H_2O_2.[61]

Cellular Assays and Stability Studies

In vitro toxicity experiments with MMn using Neuro-2A revealed that MMn displays an IC_{50} of 95 μM after an 18 h incubation time at 37°C, as inferred from a standard MTT assay (Figure S40). MMn has been previously determined to be well tolerated *in vivo*, as deduced from studies involving 2.5 mg kg^{-1} daily injections in ALS G93A mice.[55] Notably, in these same studies, HPLC analysis of Mn texaphyrin-treated mice (end of study) revealed lower levels of nitrotyrosine in the spinal cord than in untreated controls. MMn-based nanoparticles have also been evaluated *in vivo* by subjecting healthy female BABL/c mice (n = 5) to high-dose injections (10 mg kg^{-1}).[42] Again, good tolerability was seen.

In vitro stability studies in the presence of ascorbate, albumin, zinc, copper, and citric acid revealed good stability at 37°C over 24 h (Figure S41). In the presence of the oxidative mixture composed of hemin and H_2O_2, MMn displayed increased stability versus hemin and MnTMPyP when incubated at 37°C (Figure S42). MMn, MnTMPyP, and hemin all displayed moderate stability when incubated with $ONOO^-$ (2 equiv) at 37°C for 0.5 h with 80%, 84%, and 71% of these complexes, respectively, after the addition of excess NaAsc (Figure S43).

The cellular protective effect of MMn was demonstrated via MTT assays with Neuro-2A cells. Previous studies have shown that the cytotoxic effects of Aβ are dependent upon the aggregate structure (i.e., fibril, oligomer, and aggregate).[62,63] Oxidative and nitrative modifications of the peptide alters its aggregation properties and can enhance cytotoxic pathways.[64–67] We thus sought to determine how partial oxidation and nitration of $Aβ_{40}$, coupled with modification of the aggregated $Aβ_{40}$

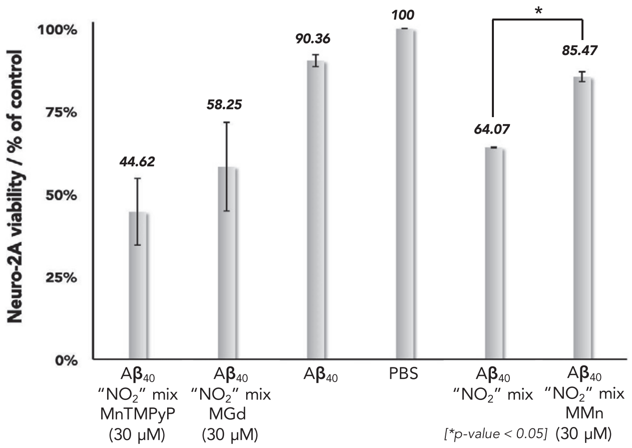

Figure 9. Cellular (Neuro-2A) MTT Assay

Neuro-2A cell viability upon exposure to $A\beta_{40}$ and mixed oxidized-nitrated $A\beta_{40}$ (hemin-H_2O_2-$NaNO_2$) with and without MMn, MGd, and MnTMPyP. Reported values are the average with standard deviation of three independent assays each tested four times. The $A\beta_{40}$ "NO_2" mix is an average of two independent assays each tested four times. *Two-sample t test on percent cell viability (MTT assay) between the $A\beta_{40}$ "NO_2" mix with and without MMn; p = 0.00016.

architecture, affects cell viability. The $A\beta_{40}$ peptide was chosen since, together with $A\beta_{42}$, it is one of the predominant Aβ species found within the AD brain. $A\beta_{40}$ also displays a slower aggregation rate and lower toxicity than $A\beta_{42}$, allowing for enhanced control in cell experiments. We assessed the protective effects of MMn and MnTMPyP by incubating $A\beta_{40}$ for 3 h at 37°C in the presence of a nitration mixture containing hemin, H_2O_2, and $NaNO_2$ in PBS. MGd was utilized as a negative control because of its ability to propagate oxidative damage.[19,23] We then stored the reaction mixture at 4°C overnight to allow the formation of additional toxic fibrils[35] before transferring it to wells containing Neuro-2A cells and subjecting it to incubation for an additional 24 h. In comparison to the unmodified $A\beta_{40}$ not subjected to the nitration conditions (90.4% ± 2%), the mixed oxidized-nitrated $A\beta_{40}$ was more toxic, resulting in only 64.1% ± 0.1% cell viability. In the presence of MMn (30 μM), the neuroblastoma Neuro-2A cell viability was increased to 85.5% ± 1.5%. MGd (30 μM) displayed cytotoxicity similar to that of the mixed oxidized-nitrated $A\beta_{40}$ system, where only 58.25% ± 13% cell viability was observed (Figure 9). MnTMPyP (30 μM) also produced a lower level of cell viability (44.62% ± 10%). On the basis of these experiments, we conclude that MMn provides a protective effect against the oxidative and nitrative modifications that are thought to yield the neurotoxic fibril and oligomeric species that lead to cell death. In the event, the incubation with MMn translates into improved cell viability.

Qualitative HPLC-MS analyses were carried out in an effort to correlate further the level of $A\beta_{40}$ oxidation and the observed survival effects seen in Neuro-2A (Figure S44). A mixture of $A\beta_{40}$ (100 μM), hemin (15 μM), and H_2O_2 (1 mM) was incubated with or without MMn (30 μM), MnTMPyP (30 μM), or MGd (30 μM) in PBS at 37°C for 3 h. MMn conferred the greatest protective effect, yielding an approximately 1:1 ratio of oxidized ($A\beta_{40}$-[O] to native $A\beta_{40}$). This stands in contrast to the sample containing MGd, where the resultant HPLC trace showed a 1.7:1 ratio of $A\beta_{40}$-[O]/$A\beta_{40}$. Finally, MnTMPyP induced a high level of oxidative modification with an approximately 3:1 ratio of $A\beta_{40}$-[O] to $A\beta_{40}$ being seen. Tandem mass spectrometry (MS/MS) analysis proved consistent with oxidation occurring primarily at His13-14 and Met35. When considered in conjunction with the N2 cell data,

the observed toxicity data are found to correlate with the levels of oxidative and nitrative modification in the case of the Aβ_{40} peptide. Previous studies have demonstrated that variations in cell toxicity are correlated with the Aβ_{40} aggregation state, meaning that they could be subject to potential modulation (i.e., enhanced or retarded toxicity) by porphyrinoid additives.[30–32] MMn appears particularly effective in this regard.

Conclusions

In conclusion, we have demonstrated that metallotexaphyrins interact with Aβ peptides and aggregates and could hold promise for the MRI-based recognition of amyloid aggregates. The first-generation water-soluble MMn that is the subject of the present study also protects against oxidative and nitrative damage of small molecules and peptides and reduces Aβ-mediated cell death. MMn was also shown to permeate the cellular membrane and be taken up within *C. elegans* animal models of AD. The present results thus serve to highlight the potential of MRI-active metalloantioxidants as potential treatment- and diagnostic-related modalities for use in the diagnosis and management of neurodegenerative disease.

EXPERIMENTAL PROCEDURES

Synthesis of Aβ_{16} and Aβ_{40}

Aβ peptides were prepared via Fmoc amino solid-phase peptide synthesis with a Liberty Blue microwave peptide synthesizer. Preparative reverse-phase high-performance liquid chromatography (RP-HPLC) purification of peptides was performed with an Agilent Zorbax SB-C_{18} Prep HT column 21.2 × 250 mm. Analytical RP-HPLC characterization of peptides was performed with an Agilent Zorbax column 4.6 × 250 mm. An Agilent Technologies 6530 Accurate Mass QT of LC/MS was used for high-resolution mass spectra of purified peptides. Solvents used were HPLC grade.

Synthesis of MGd, MMn, and MLu

Motexafin gadolinium (gadolinium(III) texaphyrin [MGd]) was a gift from Pharmacyclics Inc., recently acquired by Abbvie. MLu and MMn were synthesized according to literature protocols.[53,68]

Cell-Uptake Studies

Neuro-2A cells were grown until confluent in T-75 flasks. MMn was added (100 μM) and cells were incubated for 9 h, upon which cells were washed twice with PBS, incubated with trypsin (1×) for 2 min, diluted with supplemented Eagle's minimal essential medium (EMEM), counted, and pelleted by centrifugation (3 min at 2,000 rpm). After supernatant removal, the pellet was suspended and washed one more time with PBS (5 mL), and the pellet was reformed by centrifugation (3 min at 2,000 rpm). The cell pellet was digested in concentrated nitric acid (100 μL) for 15 h at 60°C and diluted with miliQ water to a final concentration of 2% HNO_3 before being analyzed by ICP-MS.

C. elegans Culture

The *C. elegans* strains N2 and CL4176 were provided by CGC (nematodes shipped on 35 mm diameter plates), which is funded by the NIH Office of Research Infrastructure Programs (P40-OD010440). *C. elegans* were grown and maintained as described in "WormBook: The Online Review of *C. elegans* Biology" by Theresa Stiernagle (https://www.ncbi.nlm.nih.gov/books/NBK19649/).

C. elegans MMn Uptake

After the *C. elegans* animals, N2 and CL4176, were received from the CGC (University of Minnesota) on petri dishes, agar matrix was cut in small chunks (0.5 × 0.5 cm) with a razor blade and transferred on nematode growth medium (NGM) agar plates previously seeded with *E. coli* strain OP50 as a food source. NGM plates were then placed at 16°C in order to increase the population of *C. elegans* before drug exposure and prevent significant plaque formation in the CL4176 AD model. After 7 days, MMn was added to plates (1 mL per plate of a solution at 100 μM), which were incubated at 25°C for 48 h. The animals were then transferred to Eppendorf tubes (previously weighted empty on an analytical balance) with 1.5 mL of water. The tubes were put on ice for 5 min and centrifuged (14,000 rpm for 1 min), and the supernatant was removed. The plate was washed again with 1.5 mL of water and centrifuged again. The resulting pellets were washed twice with 1 mL of DI water. *C. elegans* pellets were lyophilized over a period of 15 h before being weighed (dry pellet weights obtained by lyophilization were used to normalize the MRI and ICP-MS results).

Preparation of Peroxynitrite

Three separate solutions of $NaNO_2$ (0.6 M), HCl (0.68 M) with H_2O_2 (0.72 M), and NaOH (3.6 M) were prepared and stored in the freezer at −20°C for 30 min. The solutions were transferred to an ice bath and allowed to sit for 30 min. The $NaNO_2$ solution was added to a 100 mL beaker placed in an ice bath and stirred at >500 rpm. The HCl/H_2O_2 solution and NaOH solutions were added sequentially such that the NaOH came immediately after the HCl/H_2O_2 solution. These solutions must be added in sequence (i.e., one then the other) with as little time between the two additions as possible. The best results were obtained by adding the two solutions with a very small offset. The mixture turned yellow and was stirred in the ice bath for 5 min. MnO_2 was added portion-wise until gas evolution ceased. The mixture was split into aliquots and centrifuged, and the peroxynitrite solution was decanted with a glass pipette. The concentration could be calculated with λ_{max} = 302 nm ε = 1,670 $M^{-1}cm^{-1}$. The best results were obtained by centrifugation in a cold room at 4°C. [peroxynitrite] = 72–86 mM.

Serotonin-Hemin Reactivity Studies

Serotonin (1 mM), H_2O_2 (1 mM), hemin (15 μM), and sodium ascorbate (40 μM) without and with MMn (120 μM) or MnTMPyP (120 μM) were incubated in PBS (pH 7.4, 1.5 mL total volume) at 37°C for 2 h in the dark. The resulting mixture was passed through a reverse-phase tC18 SPE (Waters Sep-Pak, Waters) column containing 10 g of the C-18 substrate. The column was washed with 25 mL of 5% acetonitrile: H_2O to separate the serotonin and 4,4′-serotonin dimer from paramagnetic species. The resulting solution was concentrated with a rotary evaporator. The mixture was taken up in D_2O (0.7 mL) and analyzed via ^1H NMR spectroscopy. NMR spectroscopic yields were calculated by comparing the relative concentration of serotonin to the 4,4′-serotonin dimer. Note that additional washing of the column failed to yield additional serotonin or the 4,4′-serotonin dimer.

$A\beta_{16}$ and $A\beta_{40}$ Reactivity Studies

Hemin Oxidative Reactivity Conditions

Hemin (15 μM) and H_2O_2 (1 mM) and $A\beta_{16}$ (300 μM) or $A\beta_{40}$ (100 and 200 μM) in PBS (200 μL total volume) were incubated together at 37°C. The resulting mixtures were immediately subjected to HPLC-MS analysis. Vials were cooled to 4°C inside the HPLC-MS queue to prevent additional reactivity.

Hemin Nitrative Reactivity Conditions

Hemin (15 μM), $NaNO_2$ (1 mM), H_2O_2 (1 mM), and $A\beta_{16}$ (300 μM) or $A\beta_{40}$ (100 and 200 μM) in PBS (200 μL total volume) were incubated together at 37°C. The resulting mixtures were immediately subjected to HPLC-MS analysis. Vials were cooled to 4°C inside the HPLC-MS queue to prevent additional reactivity.

Hemin-Peroxynitrite Nitrative Reactivity Conditions

Hemin (15 μM), peroxynitrite (1 mM), and $A\beta_{16}$ (300 μM) in PBS (200 μL total volume) were incubated together at 37°C. All compounds were added and then vortexed to provide a homogenous mixture. Immediately after peroxynitrite was added, the mixture was vortexed again and allowed to incubate at 37°C for 30 min. The resulting mixtures were immediately subjected to HPLC-MS analysis. Vials were cooled to 4°C inside the HPLC-MS queue to prevent additional reactivity.

Cell Culture

The neuroblastoma cell line Neuro-2A was bought from ATCC. Cells were grown in EMEM containing 10% heat-inactivated fetal bovine serum (FBS) and antibiotics (100 mg/mL streptomycin and 100 U/mL penicillin) at 37°C under 5% CO_2 atmosphere. The cells were sub-cultured once every 4 to 5 days.

Neuro-2A MTT Assay

$A\beta_{40}$ (90 μM) was incubated in PBS alone or in the presence of H_2O_2 (1 mM), hemin (15 μM), and $NaNO_2$ (1 mM) (referred to as nitration mixture) for 3 h at 37°C followed by 24 h at 4°C. 250 μL of each of the solutions was transferred into each well containing about 50,000 Neuro-2A cells (plated 24 h earlier) in 250 μL of supplemented EMEM without phenol red. After 24 h, the medium was removed and replaced with 500 μL of fresh EMEM not containing FBS or phenol red. To this was added 200 μL of an MTT solution at 3 mg/mL in EMEM not containing phenol red or FBS. After 4 h at 37°C, the supernatant was removed and 200 μL of DMSO was added to each well to dissolve the formazan purple crystals. Aliquots (50 μL) of each well were transferred into a 96-well plate well, and the absorbance at 570 nm was read with a plate reader (see description above).

SUPPLEMENTAL INFORMATION

Supplemental Information can be found online at https://doi.org/10.1016/j.chempr.2019.12.016.

ACKNOWLEDGMENTS

The work in Austin was supported by the National Institutes of Health (grant RO1 CA68682 to J.L.S.) and the Robert A. Welch Foundation (F-0018 to J.L.S.). The work at MIT was supported by NIH grant UF1 NS107712 to A.J. and P.H. In addition, P.H. would like to thank a Wellcome Trust-MIT Postdoctoral Research Fellowship (105932/Z/14/Z) and a Nottingham Research Fellowship at the University of Nottingham for support. S.D. and L.C. would like to thank the Italian Ministry of Education, University, and Research (MIUR) for a Research Project of National Interest (PRIN) 2015, Prot. 2015T778JW. NMR probes at the University of Texas at Austin (UT Austin) were purchased with the shared NIH grant 1-S10-OD021508-01. The C. *elegans* strains N2 and CL4176 were provided by CGC (nematodes shipped on 35-mm-diameter plates), which is funded by the NIH Office of Research Infrastructure Programs (P40-OD010440). J.T.B. would like to thank the UT Austin for a Global Research Fellowship at the University of Pavia, Italy, and a Scientist in Residence Fellowship. H.D.R. would like to thank the UT Austin for a Provost's Graduate

Excellence Fellowship and a Scientist in Residence Fellowship. J.T.B. and G.D.T. would like to thank Dr. Ian Riddington and Andrew Kalamarides (UT Austin) for assistance with MS analyses, Professor Jonathan Pierce and Momo Sae-Lee (UT Austin) for helpful discussions on *C. elegans* work, Dr. Timothy Johnstone and Professor Kelvin Gee (UC Irvine) for helpful discussions on CNS disorders, and Dr. Steve Sorey (UT Austin) for assistance with NMR analyses.

AUTHOR CONTRIBUTIONS

J.T.B. and G.D.T. contributed equally. J.T.B. and G.D.T. conceived the project. J.F.R. synthesized the $A\beta_{16}$ and $A\beta_{40}$ peptides. J.T.B. and G.D.T. designed and performed the spectroscopic analyses and serotonin reactivity studies. G.D.T., J.T.B., and H.Z. carried out UV-vis studies. J.T.B. and G.D.T. designed and performed the Neuro-2A reactivity studies. P.H. designed and performed all of the MRI experiments. J.T.B., G.D.T., and P.H. analyzed the NMR, fluorescence, CD, reactivity studies, MRI, *C. elegans* uptake data, and ICP-MS analyses. G.D.T., H.Z., and J.W. carried out the MTT assay and cell-uptake studies. H.D.R. performed and analyzed the SEM studies. J.T.B., G.D.T., S.D., E.M., and L.C. designed and analyzed the *in vitro* HPLC-MS reactivity studies. J.T.B and S.D. performed the HPLC-MS reactivity studies. J.T.B., G.D.T., H.Z., R.M.J., and P.M. designed and performed the *C. elegans* studies. J.T.B., G.D.T, P.H., S.D., L.C., and J.L.S. prepared the manuscript. All authors approved the final version of the manuscript.

DECLARATION OF INTERESTS

The authors declare no competing interests.

Received: July 23, 2019
Revised: October 28, 2019
Accepted: December 13, 2019
Published: January 9, 2020

REFERENCES

1. Hopkins, A.L. (2008). Network pharmacology: the next paradigm in drug discovery. Nat. Chem. Biol. *4*, 682–690.
2. Albert, R. (2005). Scale-free networks in chemical biology. Journal of Cell Science *118*, 4847–4857.
3. Gitler, A.D., Dhillon, P., and Shorter, J. (2017). Neurodegenerative disease: models, mechanisms, and a new hope. Dis. Models Mech *10*, 499–502.
4. Wyss-Coray, T. (2016). Ageing, neurodegeneration, and brain rejuvenation. Nature *539*, 180–186.
5. Przedborski, S., Vila, M., and Jackson-Lewis, V. (2003). Neurodegeneration: what is it and where are we? J. Clin. Invest *1*, 3–10.
6. Monzani, E., Nicolis, S., Dell'Acqua, S., Capucciati, A., Bacchella, C., Zucca, F.A., Mosharov, E.V., Sulzer, D., Zecca, L., and Casella, L. (2019). Dopamine, oxidative stress and protein-quinone modifications in Parkinson's and other neurodegenerative diseases. Angew. Chem. Int. Ed. Engl. *58*, 6512–6527.
7. Dos Santos Picanco, L.C., Ozela, P.F., de Fatima de Brito Brito, M., Pinheiro, A.A., Padilha, E.C., Braga, F.S., de Paula da Silva, C.H.T., Dos Santos, C.B.R., Rosa, J.M.C., and da Silva Hage-Melim, L.I. (2018). Alzheimer's disease: a review from the pathophysiology to diagnosis, new perspectives for pharmacological treatment. Curr. Med. Chem. *25*, 3141–3159.
8. Kumar, A., Singh, A., and Ekavali. (2015). A review on Alzheimer's disease pathophysiology and its management: an update. Pharmacol. Rep *67*, 195–203.
9. Parikshak, N.N., Gandal, M.J., and Geschwind, D.H. (2015). Systems biology and gene networks in neurodevelopmental and neurodegenerative disorders. Nat. Rev. Genet. *16*, 441–458.
10. Arneson, D., Zhang, Y., Yang, X., and Narayanan, M. (2018). Shared mechanisms among neurodegenerative diseases: From genetic factors to gene networks. J. Genet. *97*, 795–806.
11. Cummings, J. (2018). Lessons learned from Alzheimer disease: clinical trials with negative outcomes. Clin. Transl. Sci. *11*, 147–152.
12. Yang, Z.L., and Zhang, L.J. (2016). PET/MRI of central nervous system: current status and future perspective. Eur. Radiol *26*, 3534–3541.
13. Rosén, C., Hansson, O., Blennow, K., and Zetterberg, H. (2013). Fluid biomarkers in Alzheimer's disease – current concepts. Mol. Neurodegener *8*, 20.
14. Frisoni, G.B., Fox, N.C., Jack, C.R., Scheltens, P., and Thompson, P.M. (2010). The clinical use of structural MRI in Alzheimer disease. Nat. Rev. Neurol *6*, 67–77.
15. Bolognesi, M.L., Gandini, A., Prati, F., and Uliassi, E. (2016). From companion diagnostics to theranostics: a new avenue for Alzheimer's disease? J. Med. Chem. *59*, 7759–7770.
16. Wang, X., Wang, X., and Guo, Z. (2018). Metal-involved theranostics: an emerging strategy for fighting Alzheimer's disease. Coord. Chem. Rev. *362*, 72–84.
17. Sessler, J.L., and Wegnorn, S.J. (1997). Expanded, Contracted & Isomeric Porphyrins (Elsevier).
18. Sessler, J.L., Hemmi, G., Mody, T.D., Murai, T., Burrell, A., and Young, S.W. (1994). Texaphyrins: synthesis and applications. Acc. Chem. Res. *27*, 43–50.
19. Preihs, C., Arambula, J.F., Magda, D., Jeong, H., Yoo, D., Cheon, J., Siddik, Z.H., and Sessler, J.L. (2013). Recent developments in texaphyrin

chemistry and drug discovery. Inorg. Chem. *52*, 12184–12192.

20. Mehta, M.P., Shapiro, W.R., Phan, S.C., Gervais, R., Carrie, C., Chabot, P., Patchell, R.A., Glantz, M.J., Recht, L., Sur, R.K., et al. (2009). Motexafin gadolinium combined with prompt whole brain radiotherapy prolongs time to neurologic progression in non-small-cell lung cancer patients with brain metastases: results of a phase III trial. Int. J. Radiat. Oncol. Biol. Phys. *73*, 1069–1076.

21. Rosenthal, D.I., Nurenberg, P., Becerra, C.R., Frenkel, E.P., Carbone, D.P., Lum, B.L., Miller, R., Engel, J., Young, S., Miles, D., et al. (1999). A phase I single-dose trial of gadolinium texaphyrin (Gd-Tex), a tumor selective radiation sensitizer detectable by magnetic resonance imaging. Clin. Cancer Res. *5*, 739–745.

22. Kereiakes, D.J., Szyniszewski, A.M., Wahr, D., Herrmann, H.C., Simon, D.I., Rogers, C., Kramer, P., Shear, W., Yeung, A.C., Shunk, K.A., et al. (2003). Phase I drug and light dose-escalation trial of motexafin lutetium and far red light activation (phototherapy) in subjects with coronary artery disease undergoing percutaneous coronary intervention and stent deployment: procedural and long-term results. Circulation *108*, 1310–1315.

23. Thiabaud, G., McCall, R., He, G., Arambula, J.F., Siddik, Z.H., and Sessler, J.L. (2016). Activation of platinum(IV) prodrugs by motexafin gadolinium as a redox mediator. Angew. Chem. Int. Ed. Engl. *55*, 12626–12631.

24. Batinic-Haberle, I., Tovmasyan, A., and Spasojevic, I. (2018). Mn porphyrin-based redox-active drugs- Differential effects as cancer therapeutics and protectors of normal tissue against oxidative injury. Antioxid. Redox Signal *29*, 1691–1724.

25. Zhang, X.R., Zhou, W.X., and Zhang, Y.X. (2018). Improvements in SOD mimic AEOL-10150, a potent broad-spectrum antioxidant. Mil. Med. Res. *5*, 30.

26. WCG's FDAnews (2005). Aeolus announces optimistic AEOL 10150 Phase I trial interim results, March 30, 2005. https://www.fdanews.com/articles/70464-aeolus-announces-optimistic-aeol-10150-phase-i-trial-interim-results.

27. Abu-Omar, M.M. (2011). High-valent iron and manganese complexes of corrole and porphyrin in atom transfer and dioxygen evolving catalysis. Dalton Trans. *40*, 3435–3444.

28. Gross, Z. (2001). High-valent corrole metal complexes. J. Biol. Inorg. Chem. *6*, 733–738.

29. Ghosh, C., Seal, M., Mukherjee, S., and Ghosh Dey, S.G. (2015). Alzheimer's disease: A heme-Aβ perspective. Acc. Chem. Res. *48*, 2556–2564.

30. Valiente-Gabioud, A.A., Riedel, D., Outeiro, T.F., Menacho-Márquez, M.A., Griesinger, C., and Fernández, C.O. (2018). Binding modes of phthalocyanines to amyloid β peptide and their effects on amyloid fibril formation. Biophys. J. *114*, 1036–1045.

31. Fan, Y., Wu, D., Yi, X., Tang, H., Wu, L., Xia, Y., Wang, Z., Liu, Q., Zhou, Z., and Wang, J. (2017). TMPyP inhibits amyloid-β aggregation and alleviates amyloid-induced cytotoxicity. ACS Omega *2*, 4188–4195.

32. Gomes, L.M.F., Mahammed, A., Prosser, K.E., Smith, J.R., Silverman, M.A., Walsby, C.J., Gross, Z., and Storr, T. (2019). A catalytic antioxidant for limiting amyloid-beta peptide aggregation and reactive oxygen species generation. Chem. Sci. *10*, 1634–1643.

33. Brothers, P.J. (2001). Organoelement chemistry of main-group porphyrin complexes. Adv. Organomet. Chem. *48*, 289–342.

34. Nienhaus, K., and Nienhaus, G.U. (2005). Probing heme protein-ligand interactions by UV/visible absorption spectroscopy. Methods Mol. Biol. *305*, 215–242.

35. Stine, W.B., Jungbauer, L., Yu, C., and LaDu, M.J. (2010). Preparing Synthetic Aβ in Different Aggregation States (Humana Press).

36. Xue, C., Lin, T.Y., Chang, D., and Guo, Z. (2017). Thioflavin T as an amyloid dye: fibril quantification, optimal concentration and effect on aggregation. R. Soc. Open Sci. *4*, 160696.

37. Alavez, S., Vantipalli, M.C., Zucker, D.J.S., Klang, I.M., and Lithgow, G.J. (2011). Amyloid-binding compounds maintain protein homeostasis during ageing and extend lifespan. Nature *472*, 226–229.

38. Cukalevski, R., Yang, X., Meisl, G., Weininger, U., Bernfur, K., Frohm, B., Knowles, T.P.J., and Linse, S. (2015). The Aβ40 and Aβ42 peptides self-assemble into separate homomolecular fibrils in binary mixtures but cross-react during primary nucleation. Chem. Sci. *6*, 4215–4233.

39. Wahsner, J., Gale, E.M., Rodríguez-Rodríguez, A., and Caravan, P. (2019). Chemistry of MRI contrast agents: current challenges and new frontiers. Chem. Rev. *119*, 957–1057.

40. Gale, E.M., Atanasova, I.P., Blasi, F., Ay, I., and Caravan, P. (2015). A Manganese alternative to gadolinium for MRI contrast. J. Am. Chem. Soc. *137*, 15548–15557.

41. Barandov, A., Bartelle, B.B., Gonzalez, B.A., White, W.L., Lippard, S.J., and Jasanoff, A. (2016). Membrane-permeable Mn(III) complexes for molecular magnetic resonance imaging of intracellular targets. J. Am. Chem. Soc. *138*, 5483–5486.

42. Sessler, J.L., Mody, T.D., Hemmi, G.W., Lynch, V., Young, S.W., and Miller, R.A. (1993). Gadolinium(III) texaphyrin: a novel MRI contrast agent. J. Am. Chem. Soc. *115*, 10368–10369.

43. Keca, J.M., Chen, J., Overchuk, M., Muhanna, N., MacLaughlin, C.M., Jin, C.S., Foltz, W.D., Irish, J.C., and Zheng, G. (2016). Nanotexaphyrin: one-pot synthesis of a manganese texaphyrin-phospholipid nanoparticle for magnetic resonance imaging. Angew. Chem. Int. Ed. Engl. *55*, 6187–6191.

44. Keca, J.M., Valic, M.S., Cheng, M.H.Y., Jiang, W., Overchuk, M., Chen, J., and Zheng, G. (2019). Mixed and matched metallo-nanotexaphyrin for customizable biomedical imaging. Adv. Healthc. Mater *8*, e1800857.

45. Martins, A.F., Morfin, J.-F., Geraldes, C.F.C., and Toth, E. (2014). Gd^{3+} complexes conjugated to Pittsburgh compound B: potential MRI markers of β-amyloid plaques. J Biol Inorg Chem. *19*, 281–295.

46. Dostal, V., and Link, C.D. (2010). Assaying β-amyloid toxicity using a transgenic *C. elegans* model. J. Vis. Exp *44*, e2252.

47. Zheng, S.Q., Ding, A.J., Li, G.P., Wu, G.S., and Luo, H.R. (2013). Drug absorption efficiency in *Caenorhbditis elegans* delivered by different methods. PLoS One *8*, e56877.

48. Mangialasche, F., Polidori, M.C., Monastero, R., Ercolani, S., Camarda, C., Cecchetti, R., and Mecocci, P. (2009). Biomarkers of oxidative and nitrosative damage in Alzheimer's disease and mild cognitive impairment. Ageing Res. Rev. *8*, 285–305.

49. Radi, R. (2013). Protein tyrosine nitration: biochemical mechanisms and structural basis of functional effects. Acc. Chem. Res. *46*, 550–559.

50. Fiedorowicz, M., and Grieb, P. (2012). Nitrooxidative stress and neurodegeneration. In Brain Damage – Bridging Between Basic Research and Clinics, A. Gonzalez-Quevedo, ed. (InTech Open), pp. 130–162.

51. Zhang, Y.J., Xu, Y.F., Chen, X.Q., Wang, X.C., and Wang, J.Z. (2005). Nitration and oligomerization of tau induced by peroxynitrite inhibit its microtubule-binding activity. FEBS Lett. *579*, 2421–2427.

52. Butterfield, D.A., Swomley, A.M., and Sultana, R. (2013). Amyloid β-Peptide (1-42)-induced oxidative stress in Alzheimer disease: importance in disease pathogenesis and progression. Antioxid. Redox Signal *19*, 823–835.

53. Hannah, S., Lynch, V., Guldi, D.M., Gerasimchuk, N., MacDonald, C.L.B., Magda, D., and Sessler, J.L. (2002). Late first-row transition-metal complexes of texaphyrin. J. Am. Chem. Soc. *124*, 8416–8427.

54. Shimanovich, R., Hannah, S., Lynch, V., Gerasimchuk, N., Mody, T.D., Magda, D., Sessler, J.L., and Groves, J.T. (2001). Mn(II)-texaphyrin as a catalyst for the decomposition of peroxynitrite. J. Am. Chem. Soc. *123*, 3613–3614.

55. Crow, J.P. (2005). Administration of Mn porphyrin and Mn texaphyrin at symptom onset extends survival of ALS mice. In Medicinal inorganic chemistry, ACS Symp. S., J.L. Sessler, S.R. Doctrow, J.T. McMurry, and S.J. Lippard, eds., pp. 295–318.

56. Groves, J.T., and Marla, S.S. (1995). Peroxynitrite-induced DNA strand scission mediated by a manganese porphyrin. J. Am. Chem. Soc. *117*, 9578–9579.

57. Chesler, M. (2003). Regulation and modulation of pH in the brain. Physiol. Rev. *83*, 1183–1221.

58. Arasasingham, R.D., He, G.X., and Bruice, T.C. (1993). Mechanism of manganese porphyrin-catalyzed oxidation of alkenes. Role of manganese(IV)-oxo species. J. Am. Chem. Soc. *115*, 7985–7991.

59. Thiabaud, G., Pizzocaro, S., Garcia-Serres, R., Latour, J.M., Monzani, E., and Casella, L. (2013). Heme binding induces dimerization and nitration of truncated β-amyloid peptide Aβ16

under oxidative stress. Angew. Chem. Int. Ed. Engl *52*, 8041–8044.

60. Hiner, A.N.P., Raven, E.L., Thorneley, R.N.F., García-Cánovas, F., and Rodríguez-López, J.N. (2002). Mechanisms of compound I formation in heme peroxidases. J. Inorg. Biochem *91*, 27–34.

61. Batinić-Haberle, I., Benov, L., Spasojević, I., and Fridovich, I. (1998). The *Ortho* effect makes manganese(III)*meso*-tetrakis(*N*-Methylpyridinium-2-yl)porphyrin a powerful and potentially useful superoxide dismutase mimic. J. Biol. Chem. *273*, 24521–24528.

62. Davies, M.J. (2016). Protein oxidation and peroxidation. Biochem. J. *473*, 805–825.

63. Irie, K., Murakami, K., Masuda, Y., Morimoto, A., Ohigashi, H., Ohashi, R., Takegoshi, K., Nagao, M., Shimizu, T., and Shirasawa, T. (2005). Structure of β-amyloid fibrils and its relevance to their neurotoxicity: implications for the pathogenesis of Alzheimer's disease. J. Biosci. Bioeng *99*, 437–447.

64. Kummer, M.P., Hermes, M., Delekarte, A., Hammerschmidt, T., Kumar, S., Terwel, D., Walter, J., Pape, H.C., König, S., Roeber, S., et al. (2011). Nitration of tyrosine 10 critically enhances amyloid β aggregation and plaque formation. Neuron *71*, 833–844.

65. Yuan, C., Yi, L., Yang, Z., Deng, Q., Huang, Y., Li, H., and Gao, Z. (2012). Amyloid beta-heme peroxidase promoted protein nitrotyrosination: relevance to widespread protein nitration in Alzheimer's disease. J. Biol. Inorg. Chem. *17*, 197–207.

66. Hou, L., Shao, H., Zhang, Y., Li, H., Menon, M.K., Neuhaus, E.B., Brewer, J.M., Byeon, J.M., Ray, D.G., Vitek, M.P., et al. (2004). Solution NMR studies of the Aβ(1–40) and Aβ(1–42) peptides establish that the Met35 oxidation state affects the mechanism of amyloid formation. J. Am. Chem. Soc. *126*, 1992–2005.

67. Guivernau, B., Bonet, J., Valls-Comamala, V., Bosch-Morató, M., Godoy, J.A., Inestrosa, N.C., Perálvarez-Marín, A., Fernández-Busquets, X., Andreu, D., Oliva, B., et al. (2016). Amyloid-β peptide nitrotyrosination stabilizes oligomers and enhances NMDAR-mediated toxicity. J. Neurosci *36*, 11693–11703.

68. Sessler, J.L., Mody, T.D., Hemmi, G.W., and Lynch, V. (1993). Synthesis and structural characterization of lanthanide(III) texaphyrins. Inorg. Chem. *32*, 3175–3187.

Chem, Volume *6*

Supplemental Information

Metallotexaphyrins as MRI-Active Catalytic Antioxidants for Neurodegenerative Disease: A Study on Alzheimer's Disease

James T. Brewster II, Gregory D. Thiabaud, Peter Harvey, Hadiqa Zafar, James F. Reuther, Simone Dell'Acqua, Rachel M. Johnson, Harrison D. Root, Pedro Metola, Alan Jasanoff, Luigi Casella, and Jonathan L. Sessler

Supplemental Data Items

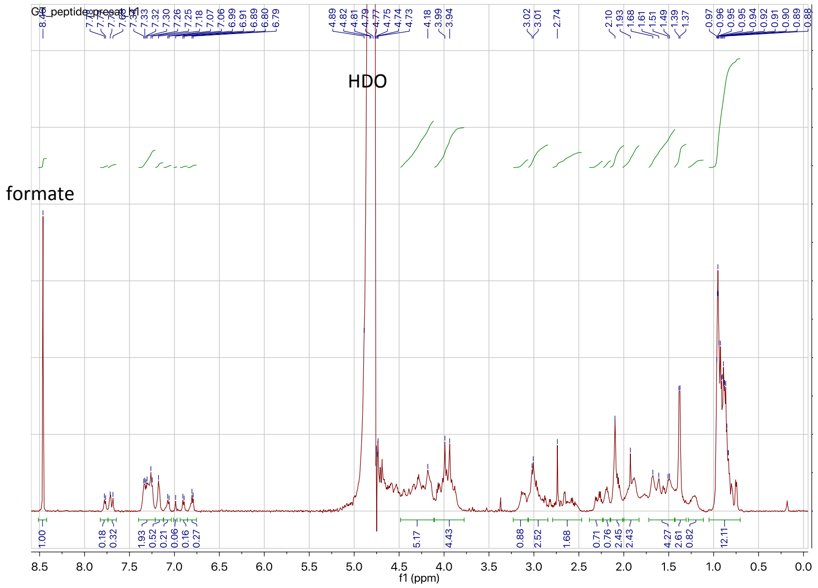

Figure S1: ^{1}H NMR spectrum of Aβ_{40} peptide (10% D_2O in PBS, 600 MHz, 298 K)

Target Compound Screening Report

Results Acquired by The University of Texas at Austin Mass Spectrometry Facility

**Data File**	MSF18-1297(GT923)_hrESIposcolumn1.d	**Sample Name**	1297(GT923)	**Comment**	1297(GT923)
**Position**	P1-B6	**Instrument Name**	Instrument 1	**User Name**	
**Acq Method**	New_pos_column_12min.m	**Acquired Time**	9/19/2018 12:30:45 PM	**DA Method**	KS.m

MS Zoomed Spectrum

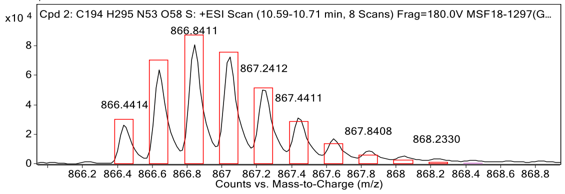

MS Spectrum Peak List

Obs. m/z	Calc. m/z	Charge	Abundance	Formula	Ion Species	Tgt Mass Error (ppm)
866.4414	866.4370	5	27333	C194H295N53O58S	(M+5H)+5	-5.17
866.6411	866.6375	5	64013	C194H295N53O58S	(M+5H)+5	-4.07
866.8411	866.8381	5	81273	C194H295N53O58S	(M+5H)+5	-3.51
867.0408	867.0386	5	73297	C194H295N53O58S	(M+5H)+5	-2.53
867.2412	867.2390	5	52042	C194H295N53O58S	(M+5H)+5	-2.45
867.4411	867.4395	5	31967	C194H295N53O58S	(M+5H)+5	-1.87
867.6407	867.6400	5	17455	C194H295N53O58S	(M+5H)+5	-0.9
867.8408	867.8404	5	9429	C194H295N53O58S	(M+5H)+5	-0.47
868.0397	868.0409	5	5678	C194H295N53O58S	(M+5H)+5	1.37
868.2330	868.2413	5	3975	C194H295N53O58S	(M+5H)+5	9.54

--- End Of Report ---

Agilent Technologies — Page 1 of 1 — Printed at: 1:00 PM on:9/19/2018

Figure S2: High-resolution mass spectrum (ESI) of Aβ_{40} peptide as $[M+5H^{+}]^{5+}$ species

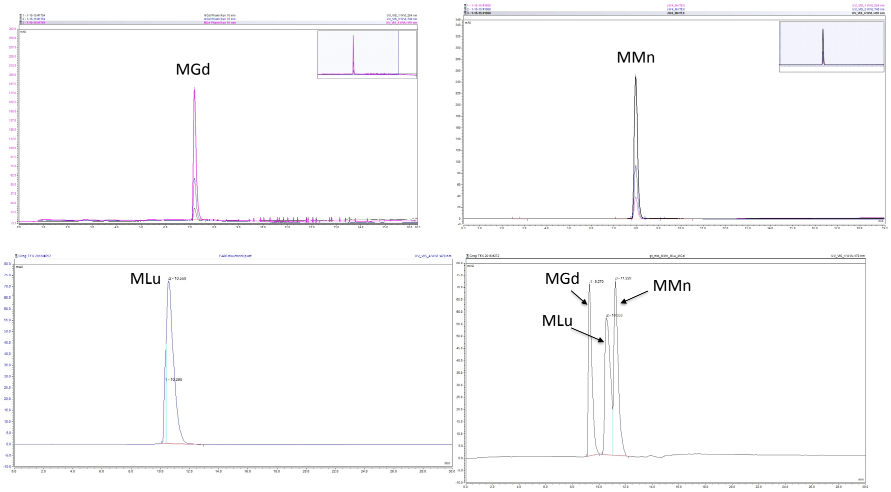

Figure S3: RP-HPLC chromatograms (detector set at 254, 470, and 740 nm) of MGd (top left), MLu (bottom left), MMn (top right), and co-elution of the three metallotexaphyrins (bottom right).

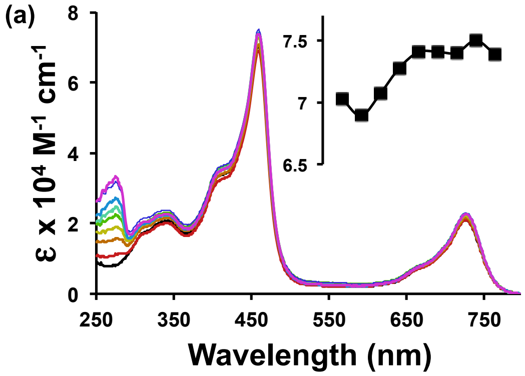

Figure S4. UV-vis spectra of MMn (5 μM) recorded upon the addition of Aβ_{40} (0-20 equiv.; black to pink) in PBS (pH 7.15) at 23 °C and insert of the molar absorptivity at $\lambda = 459$ nm (0-20 equiv.; in 2.5 equiv. aliquots). *An Aβ_{40} stock solution was prepared, the appropriate equivalents were added to a 1 mL Eppendorf tube, lyophilized, and re-dissolved in 1 mL of an MMn (5 μM) solution. The calculated value for the 17.5 equivalent data point is approximately 19 equivalents and was attributed to the method of preparation (i.e., transfer and lyophilize).*

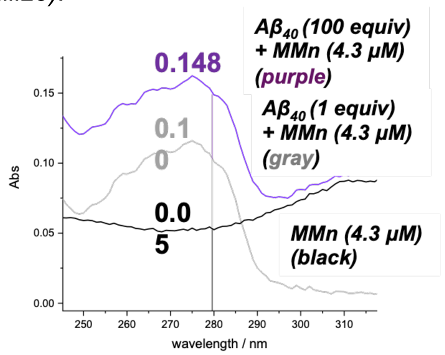

$$Abs_{MMn} + Abs_{Ab40} \approx Abs_{Ab40+MMn}$$

⇔ almost no precipitation of Aβ_{40}

Figure S5. UV-vis spectra of MMn (4.3 μM; *black*), Aβ_{40} (67 μM; *gray*), and MMn plus Aβ_{40} (*purple*) in PBS (pH 7.15) at 23 °C. Note: The molar absorptivity of the Aβ_{40} peptide (ε) is 1,480 $M^{-1}cm^{-1}$ at 280 nm.

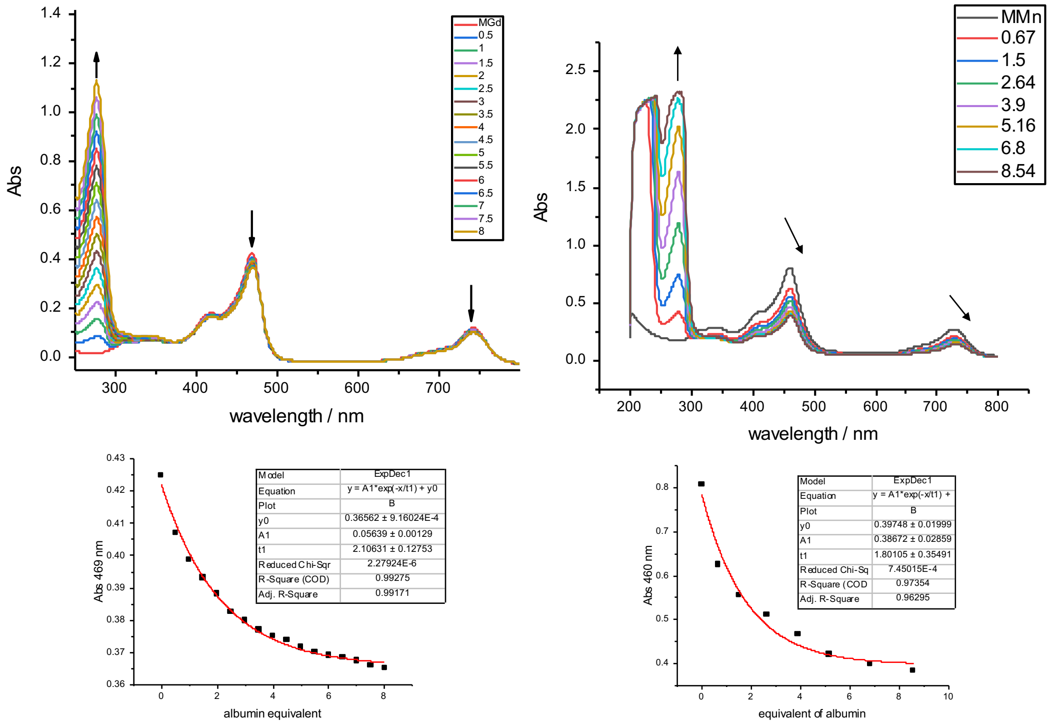

Figure S6. UV-vis (PBS, 300 K) spectral titrations of (left) MGd (5.6 μM) and (right) MMn (11.8 μM) with human serum albumin (HSA). Under each titration is plotted the evolution of the λ_{max} (Soret band) intensity against the number of equivalents of albumin added.

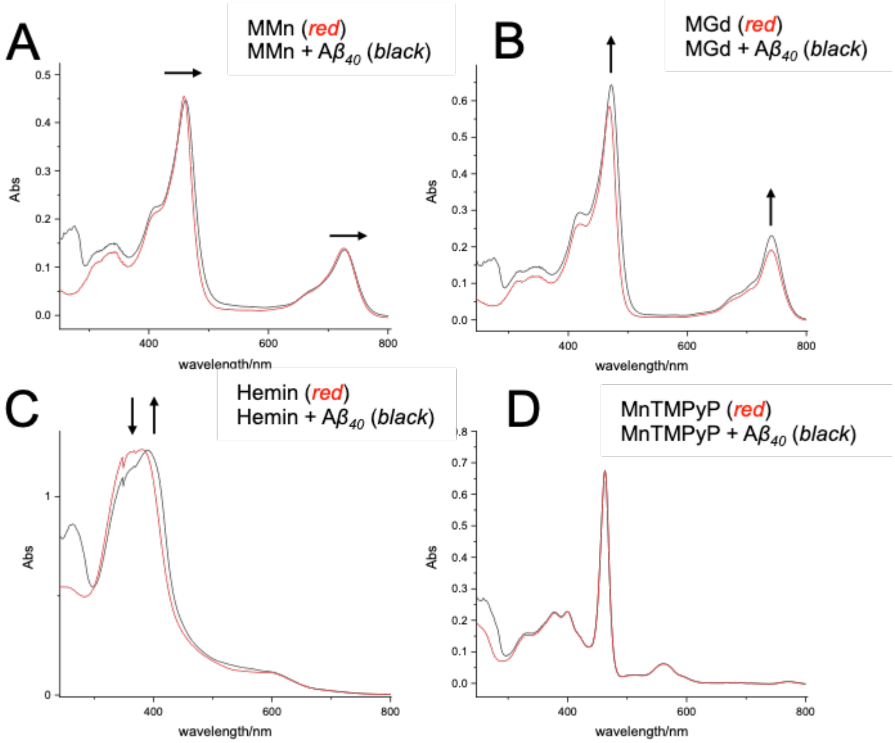

Figure S7. UV-vis spectra (300 K, PBS) of (a) MMn (7 μM), (b) MGd (7 μM), (c) hemin (14μM), and (d) Mn^{III}TM-4-PyP (7 μM) before (red) and after (black) addition of monomeric $A\beta_{40}$ (10 equiv.).

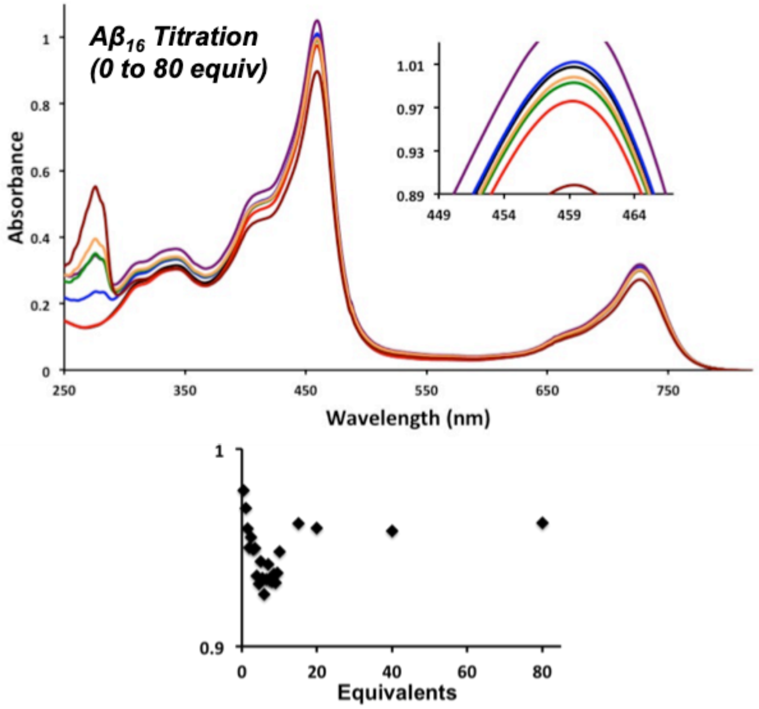

Figure S8. UV-vis spectra of MMn (5 μM) recorded upon the addition of $A\beta_{16}$ [0-80 equiv.; *black* (0 equiv.), *red* (1 equiv.), *blue* (10 equiv.), *purple* (20 equiv.), *green* (30 equiv.), *yellow* (40 equiv.), and *brown-red* (80 equiv.)] in PBS (pH 7.15) at 23 °C and insert of the molar absorptivity focused on $\lambda = 459$ nm (0-80 equiv.). Also shown is a plot of the absorbance at $\lambda = 459$ nm as a function of added $A\beta_{16}$ (0-80 equiv.).

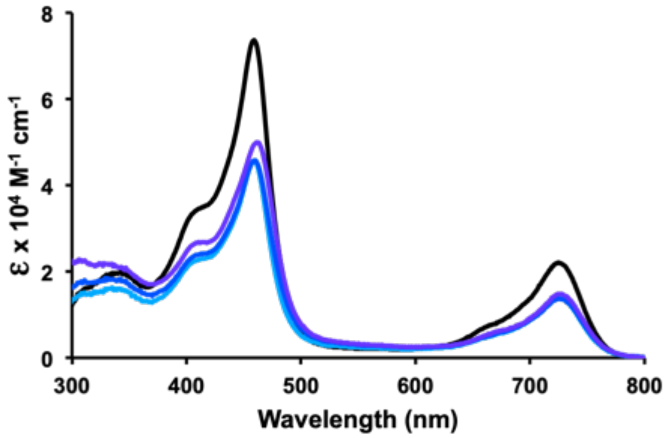

Figure S9. UV-vis spectra of MMn (5 μM) recorded upon the addition of oligomeric $A\beta_{40}$; 0 (black), 1 (light blue), 5 (blue), and 20 equiv. (purple) in PBS (pH 7.15) at 23 °C.

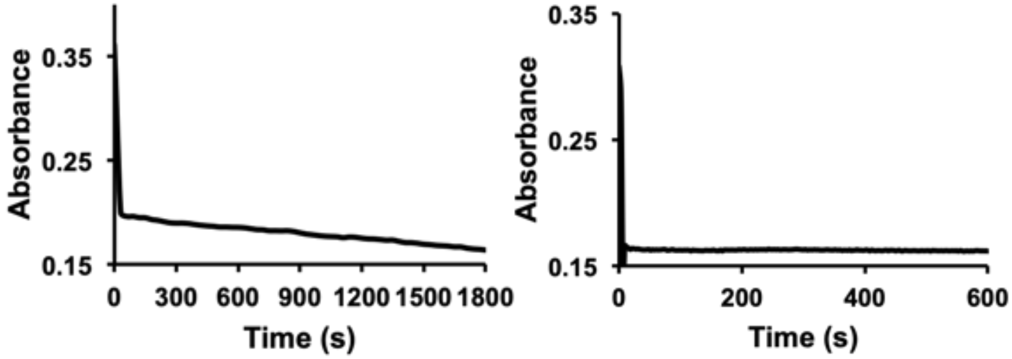

Figure S10. (left) Inferred binding of MMn (5 μM) to 2-month aged Aβ_{40} aggregates (100 μM) as determined by monitoring the changes in UV-vis spectral intensity (Abs at 460 nm in PBS at 300 K). Also shown (right) is the corresponding trace for MMn alone, which is taken as evidence that the change seen in the left is not due to photobleaching.

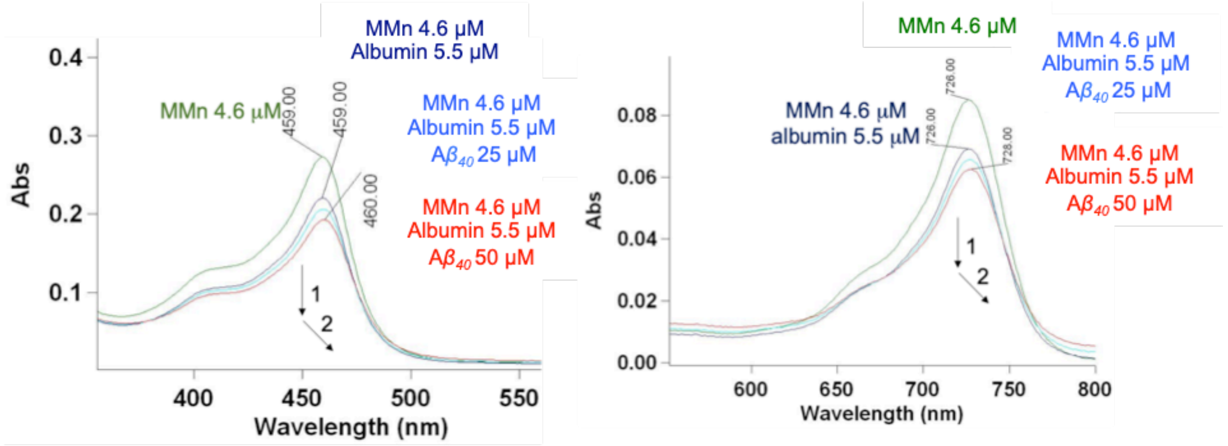

Figure S11. UV-vis spectra of MMn (4.6 μM) recorded in the presence and absence of albumin (5.5 μM) and with and without Aβ_{40} (25 and 50 μM). (left) Soret and (right) Q-band.

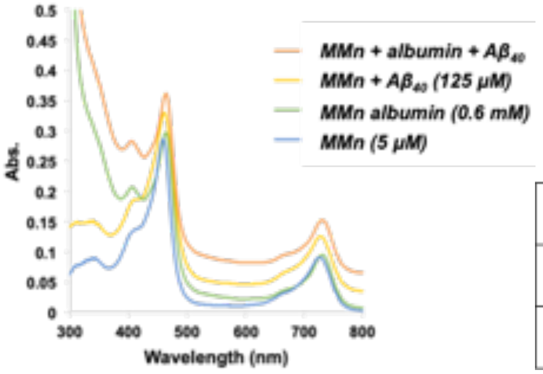

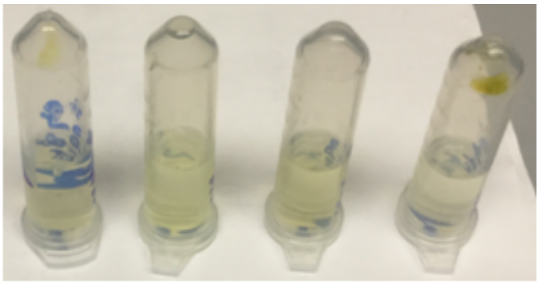

MMn 5 μM	+	+	+	+
Albumin 0.6 mM	+		+	
Aβ40 125 μM	+			+

Figure S12. (left) UV-vis spectra (PBS, 300 K) of MMn (5 μM) recorded in the absence and in the presence of albumin (0.6 mM) and with and without Aβ_{40} (125 μM) added in solid form. (right) The picture displays the same solutions after storing at 4 °C for 48 h. Pellets were obtained by centrifugation at 14000 rpm for 5 min. ***Note***: *the baseline shows the absorbance seen upon the addition of amyloid peptide and is thought to reflect its partial insolubility*.

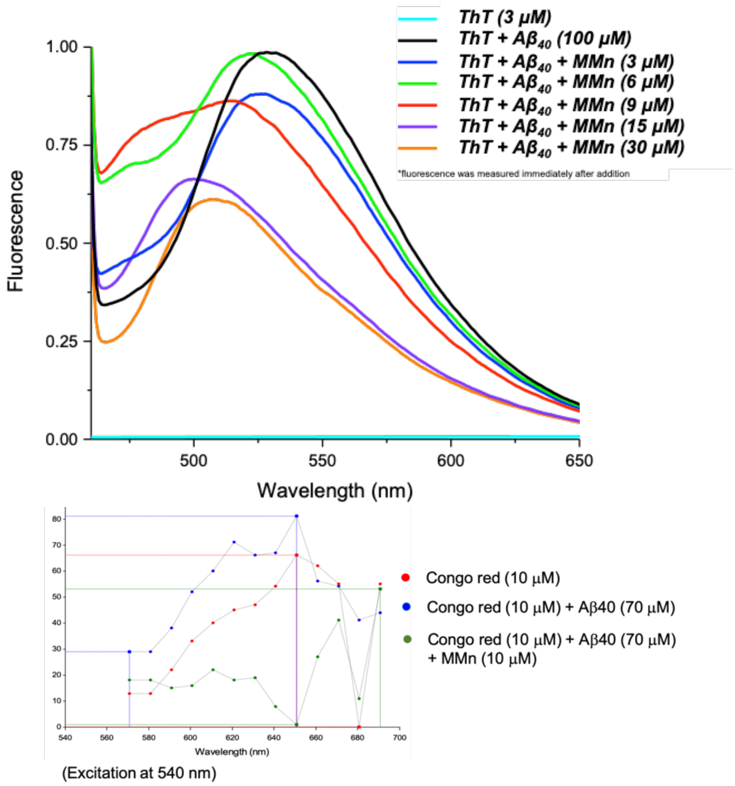

Figure S13. (top) Thioflavin T (ThT) fluorescence displacement assay using ThT (3 μM) with 1-week aged oligomeric Aβ_{40} (100 μM) and changes seen upon the addition of MMn (0 to 10 equiv.). (bottom) Congo Red (CR) fluorescence displacement assay using CR (10 μM) with 1-month aged aggregated Aβ_{40} (70 μM) and changes seen upon the addition of MMn (10 μM).

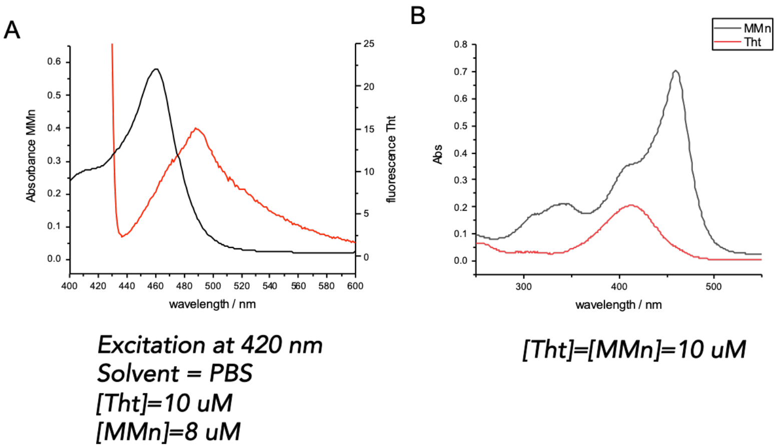

Figure S14. (a) Overlaid fluorescence and (b) UV-vis spectra of MMn (*black*) and ThT (*red*).

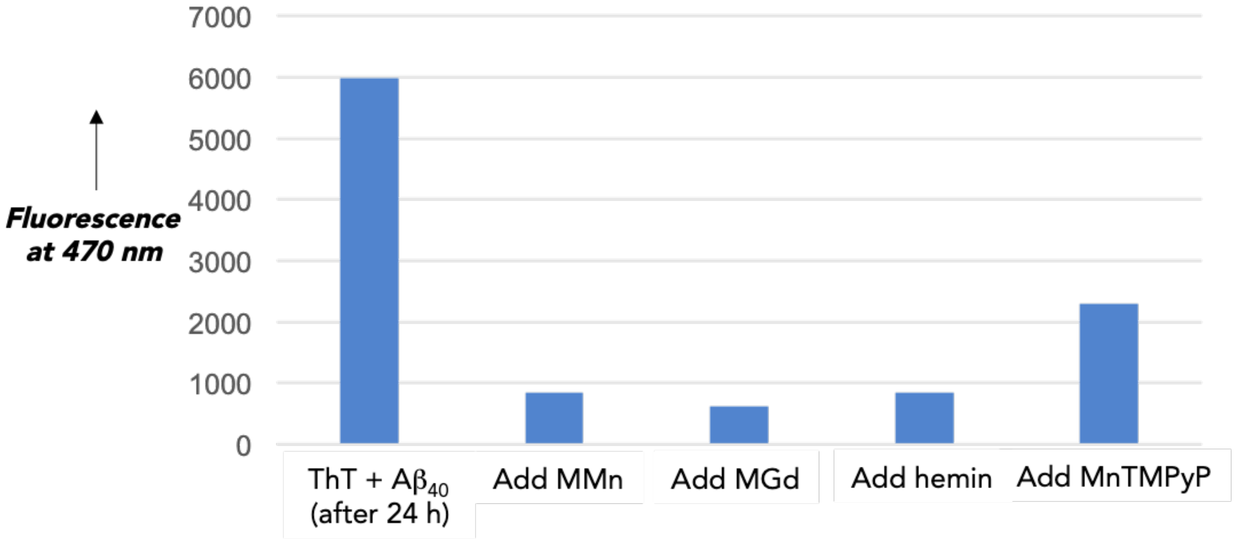

Figure S15. Fluorescence intensity values for ThT-Aβ_{40} alone and with MMn, MGd, hemin, or MnTMPyP.

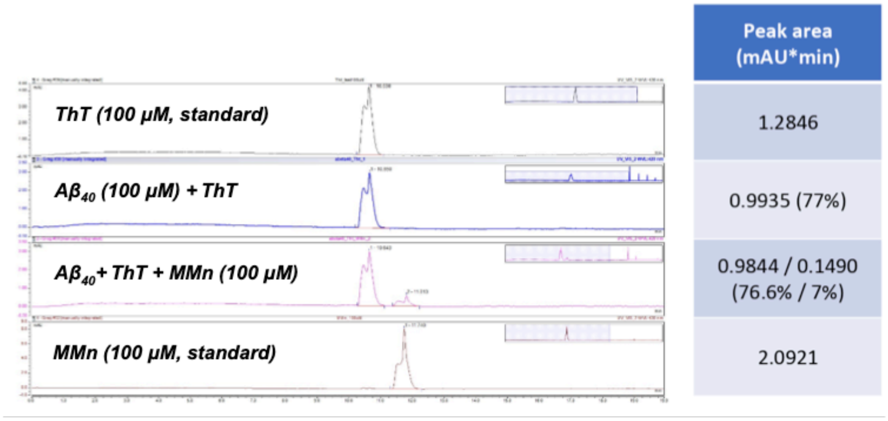

Figure S16. HPLC-MS analysis of ThT (100 μM) and MMn (100 μM) with 2-month aged Aβ_{40} (100 μM). Samples were incubated in PBS at 37 °C for 24 h then centrifuged before the supernatant was analyzed by HPLC. The area under the curve for ThT was measured at 420 nm while a wavelength of 470 nm used for MMn.

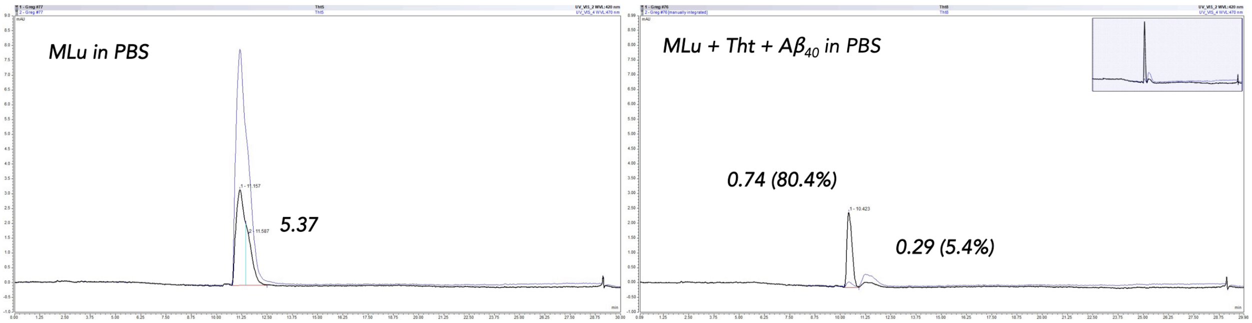

Figure S17. HPLC-MS analysis of ThT (100 μM) and MLu (100 μM) uptake within 2-month aged Aβ_{40} (67 μM). Samples were incubated in PBS at 37 °C for 40 h then centrifuged before the supernatant was analyzed by HPLC. The area under the curve for ThT was measured at 420 nm while a wavelength of 470 nm used for MMn.

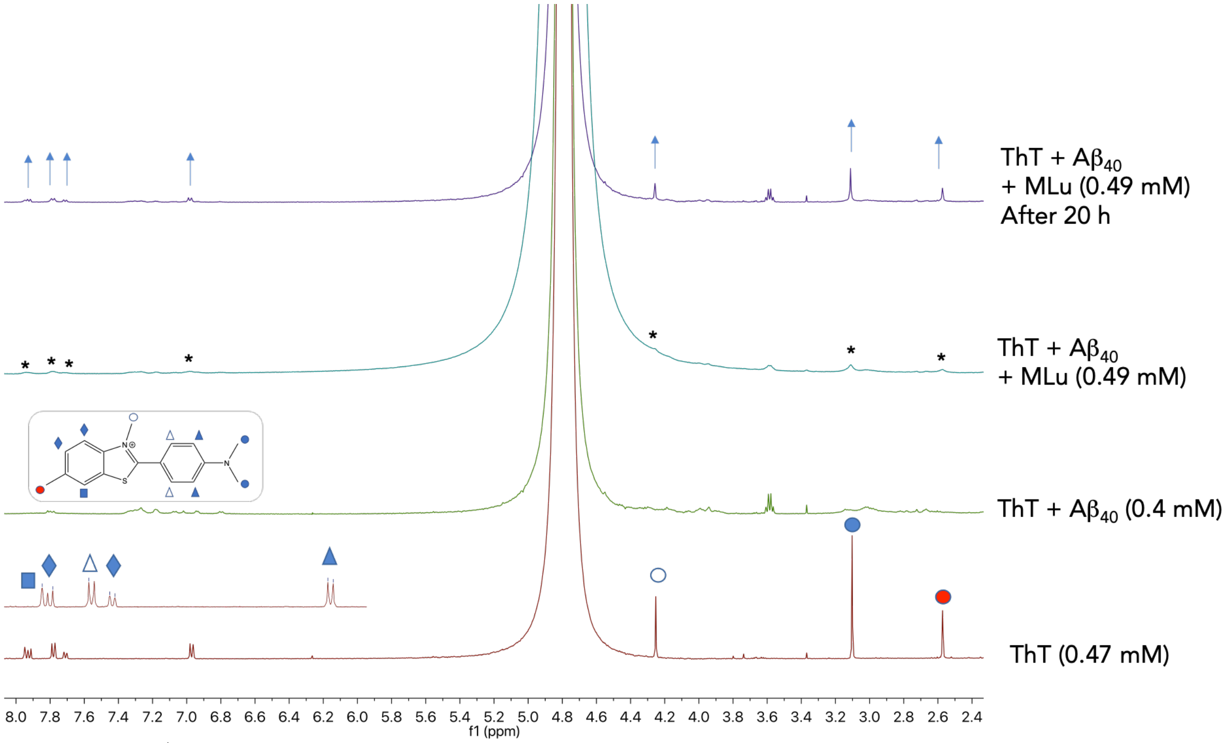

Figure S18. ^{1}H NMR spectra (500 MHz, 300 K, D_2O) of ThT with $A\beta_{40}$ and with $A\beta_{40}$ and MLu. The spectrum of the latter sample was recorded immediately after addition and after a 20 h incubation period at room temperature.

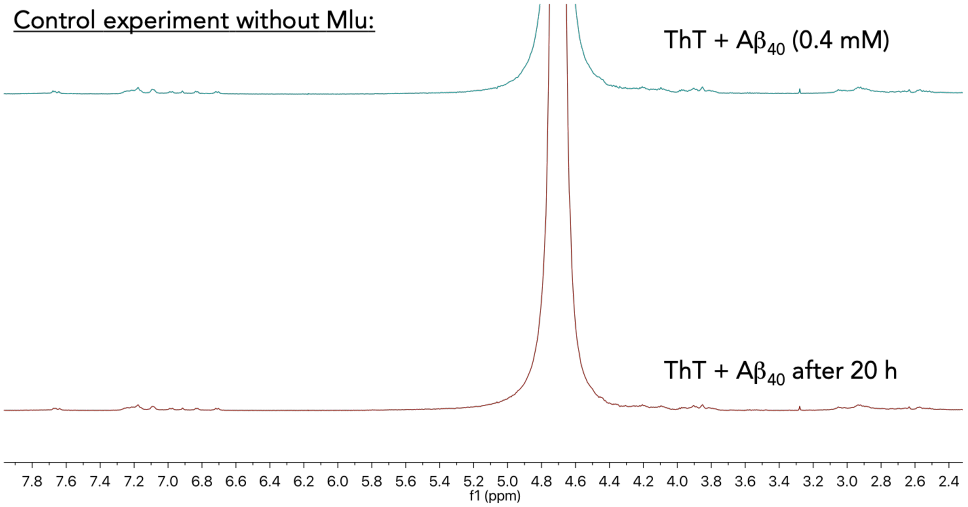

Figure S19. Control experiment showing ThT is not displaced when incubated with $A\beta_{40}$ under the same conditions.

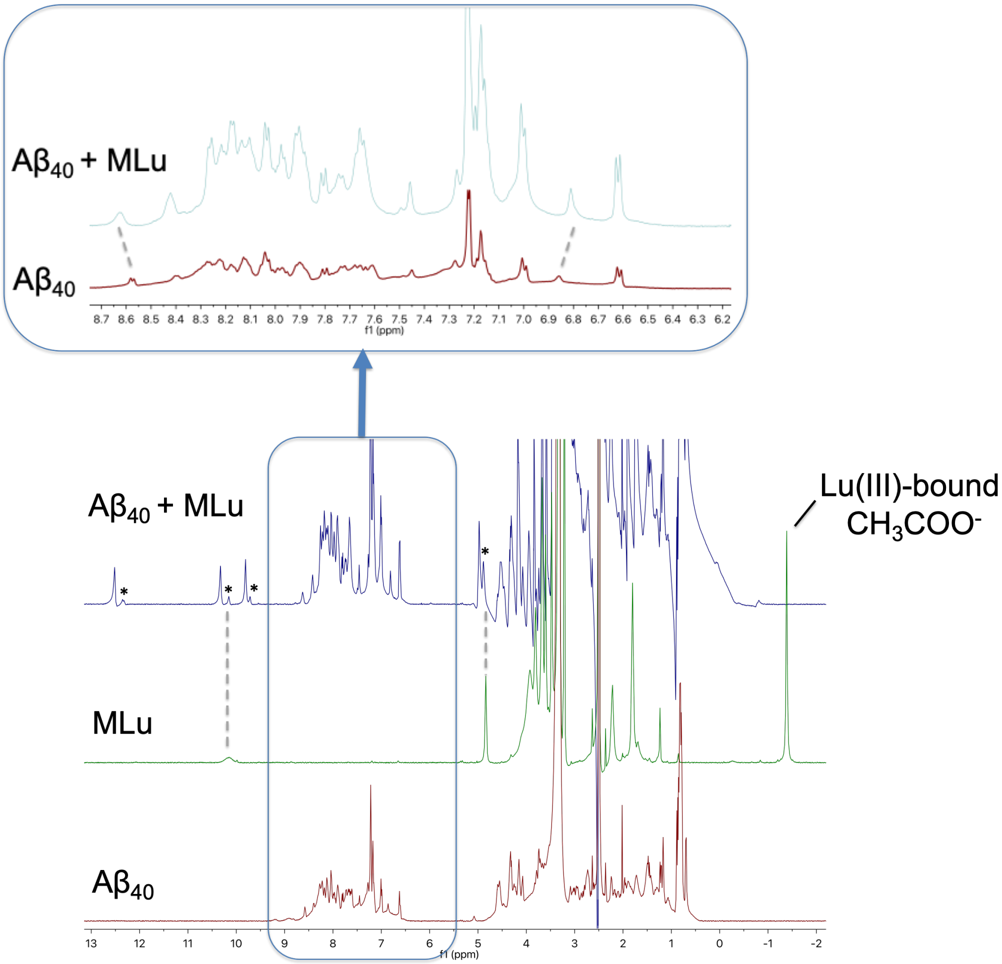

Figure S20. ^{1}H NMR spectra (500 MHz, 300 K, DMSO-d_6) of Aβ_{40} (0.66 mM, freshly prepared), MLu (2.78 mM), and a mixture of Aβ_{40} with MLu ([Aβ_{40}] = 1.9 mM, [MLu] = 2.1 mM). *Upon addition of the Aβ_{40} peptide, the acetate peak was observed to shift from its original position at -1.5 ppm.*

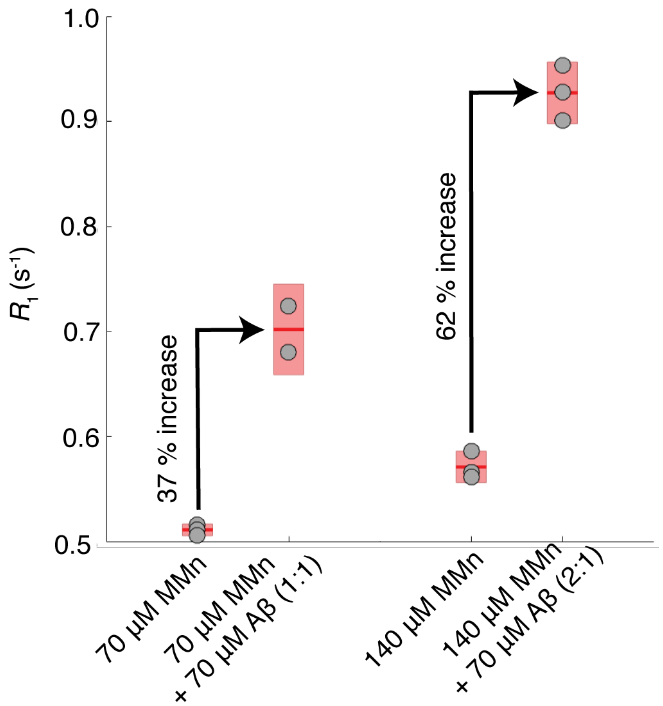

Figure S21. Variation in R_1 seen upon the addition of one and two equiv. of MMn to fully aggregated Aβ_{40} (aged >2 months).

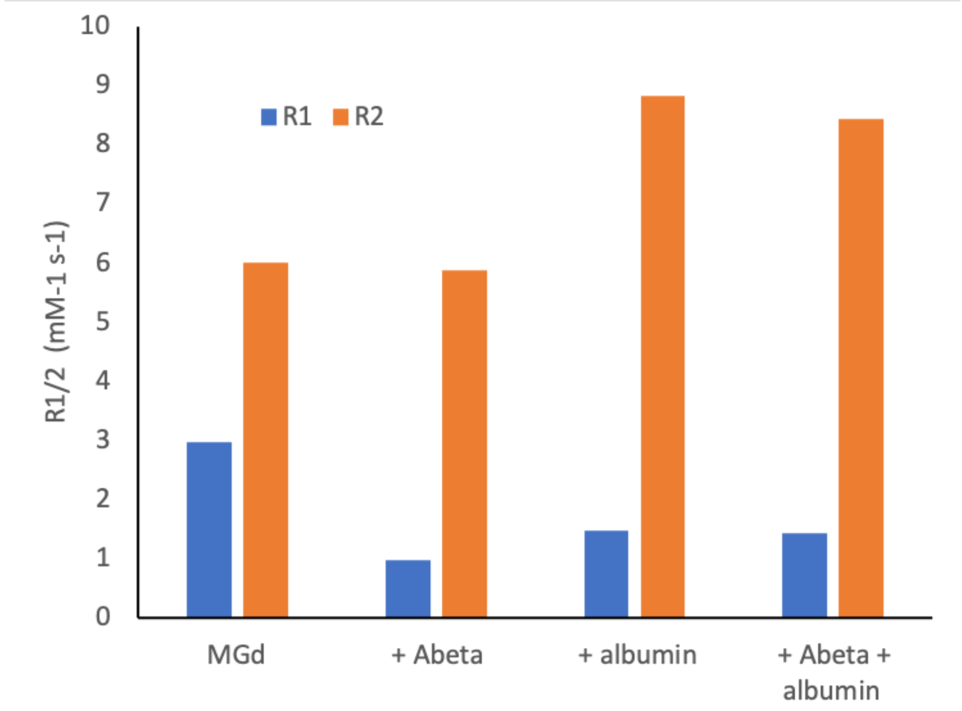

Figure S22. MRI (R_1 & R_2) analyses of MGd alone and in the presence of aggregated Aβ_{28} (500 μM), BSA (600 μM), and a combination of Aβ_{28} and BSA.

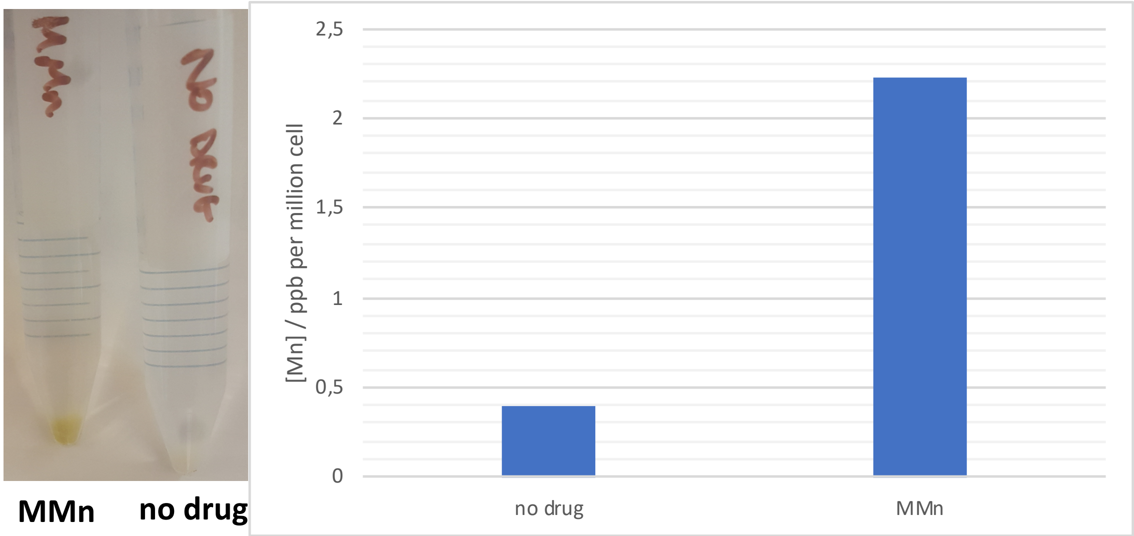

Figure S23. (left) Neuro-2A cell pellet images and (right) Mn concentration of the MMn ("drug") and no drug cell pellets measured by ICP-MS. Values are in ppb per million cells. Neuro-2A cells were grown in 5% CO_2 at 37 °C in EMEM supplemented with 10% fetal bovine serum (Invitrogen-Gibco) for 9 hours. MMn was dosed at 30 μM.

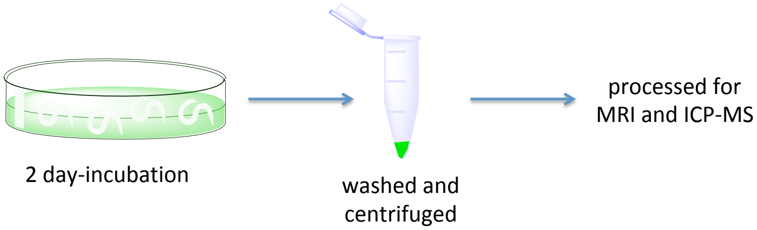

Figure S24. Preparation of *C. elegans* for MRI and ICP-MS analysis. *Pellets weighing over 2.5 mg were discarded as these samples were more than one standard deviation from the mean pellet weight.*

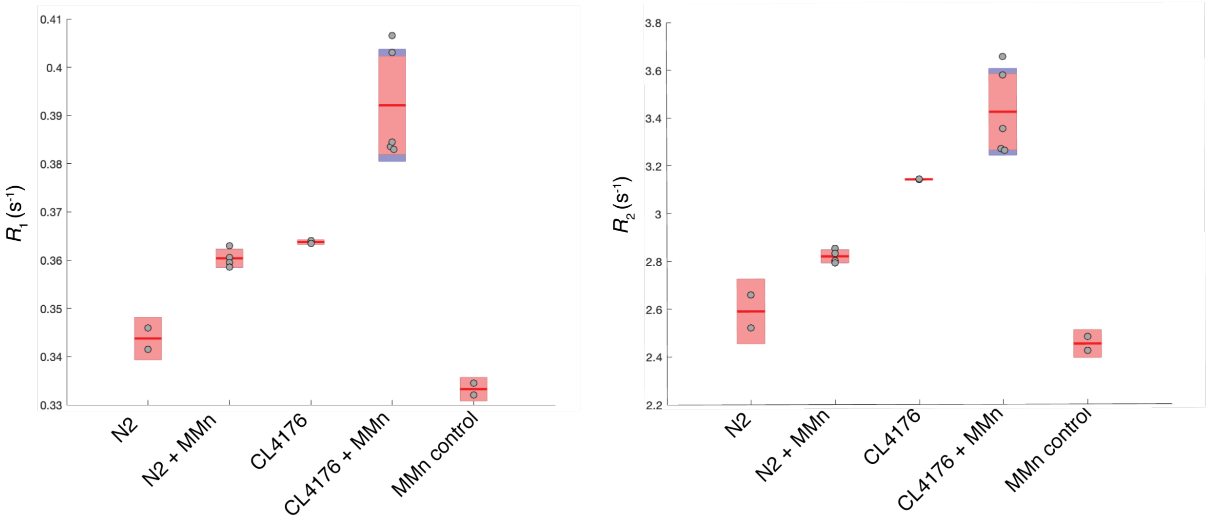

Figure S25. MRI data of *C. elegans* samples in HEPES buffer (1 mg pellet/ 100 μL HEPES).

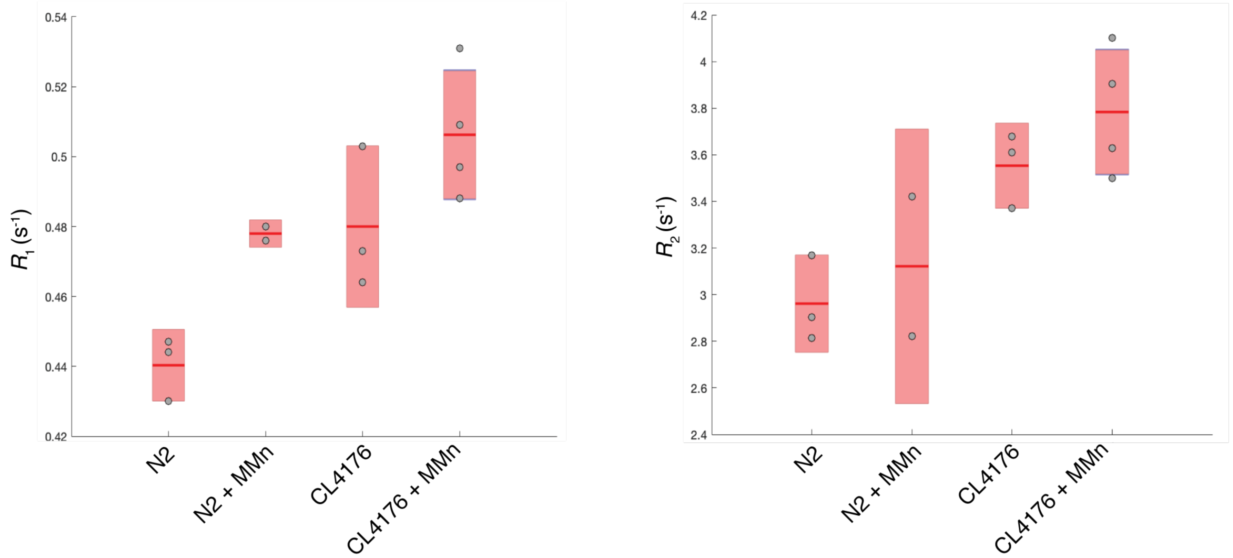

Figure S26. MRI data of a second batch *C. elegans* samples in HEPES buffer (1 mg pellet/ 100 µL HEPES). Differences between experiments may reflect varying levels of metabolic degradation or decomposition following the experiment yielding varying levels of MMn and free manganese ions with a different MRI signal.

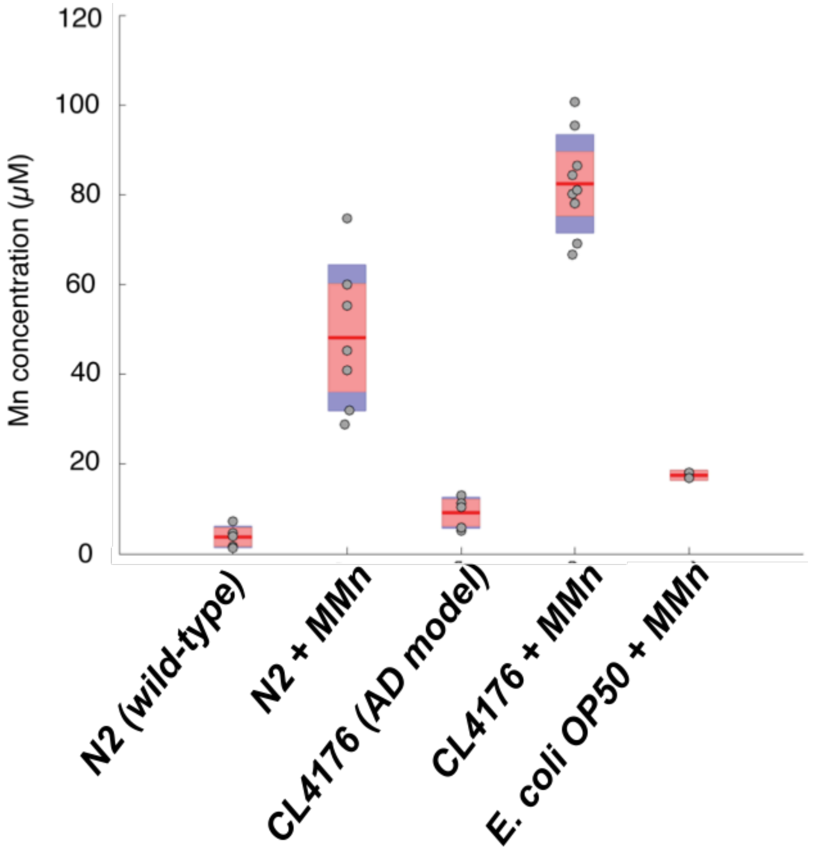

Figure S27. Complete ICP-MS analysis showing wild-type (N2) with and without MMn, AD model (CL4176) with and without MMn, and the "No Worm" control containing only the *E. coli* food source incubated with MMn.

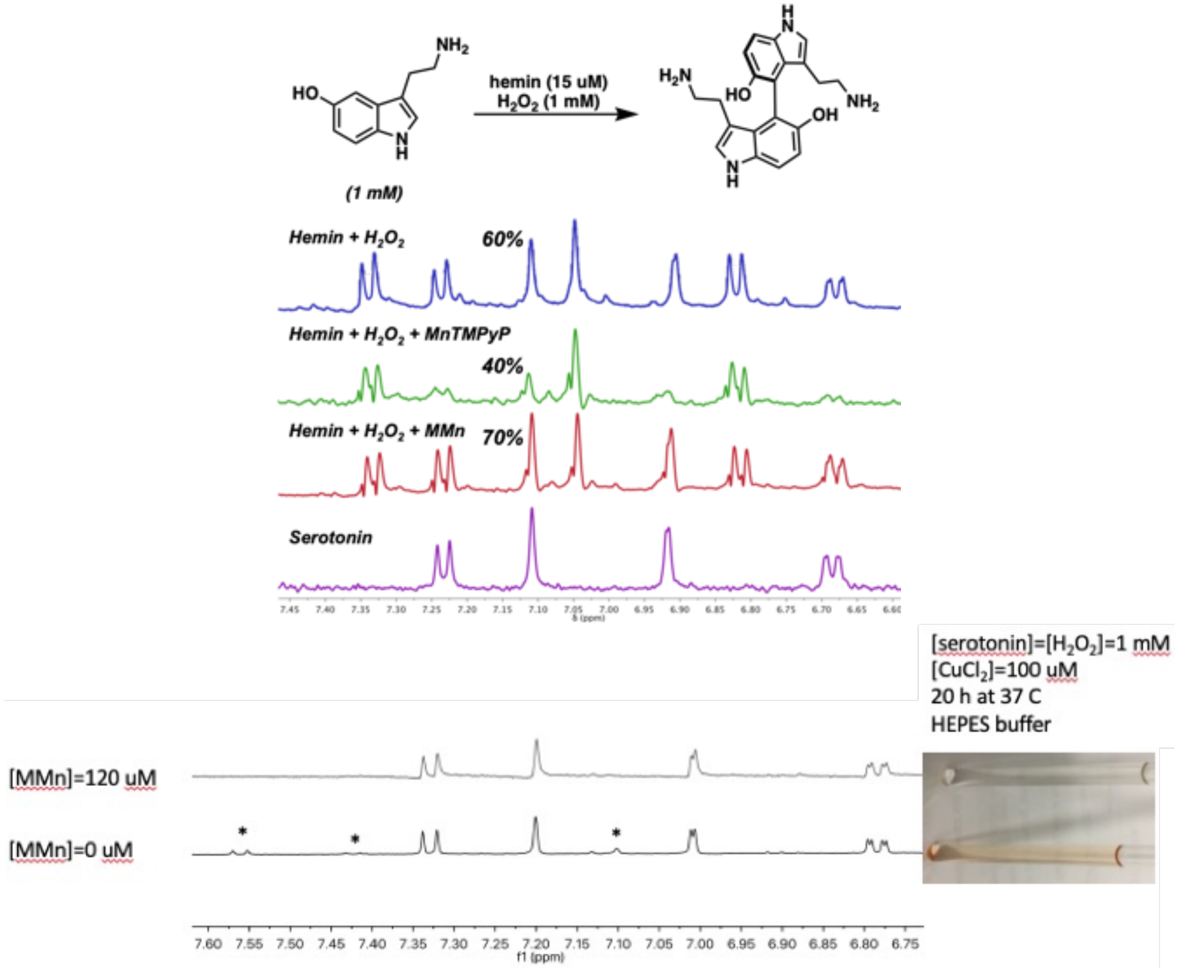

Figure S28. (top) Quantitative ^{1}H NMR spectral analysis of hemin-mediated oxidation of serotonin with MMn and MnTMPyP. (bottom) Qualitative ^{1}H NMR spectral analysis of Cu(II)-mediated oxidation of serotonin with and without MMn.

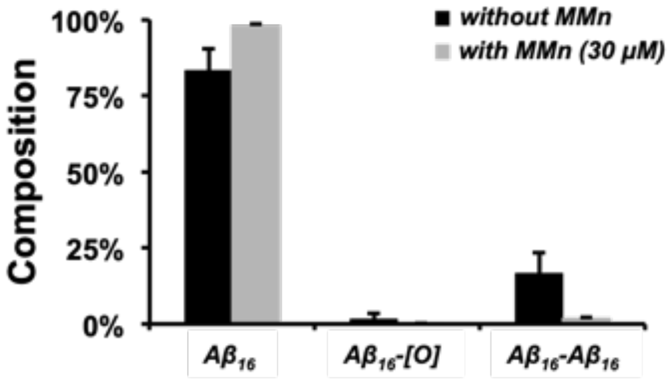

Figure S29. Oxidative modification of Aβ_{16} (300 μM) using hemin (15 μM) and H_2O_2 (1 mM) in 5 mM phosphate buffer (pH 7.4). Precise values with standard deviations were calculated for Aβ_{16}, Aβ_{16} containing an oxidized histidine residue (H13/H14), and the Aβ_{16}-Aβ_{16} tyrosine dimer. Each experiment is an average of two independent runs. MS/MS spectra of the ion series obtained upon peptide fragmentation confirms that nitration occurs to Tyr10. MS/MS fragmentation also indicated that His13 or His14 are oxidized.

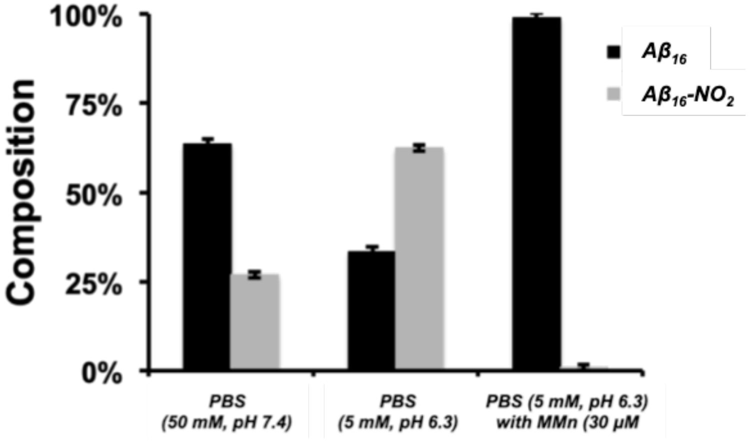

Figure S30. Nitrative modification of Aβ16 (300 μM) using hemin (15 μM), H_2O_2 (1 mM), and $NaNO_2$ (1 mM) using 5 and 50 mM phosphate buffer at pH 7.4 and 6.3. Precise values with standard deviations were calculated for Aβ16 and Aβ16 containing a nitrated tyrosine residue. Each experiment is an average of two independent runs.

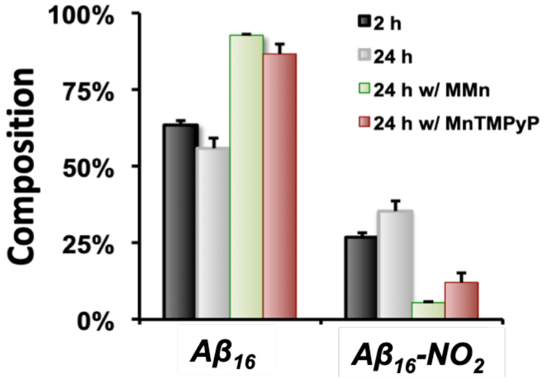

Figure S31. Nitrative modification of Aβ16 (300 μM) using hemin (15 μM), H_2O_2 (1 mM), and $NaNO_2$ (1 mM) in 5 mM phosphate buffer at pH 7.4 with and without MMn (30 μM) or MnTMPyP (30 μM). Precise values with standard deviations were calculated for Aβ16 and Aβ16 containing a nitrated tyrosine residue. Each experiment is an average of two independent runs.

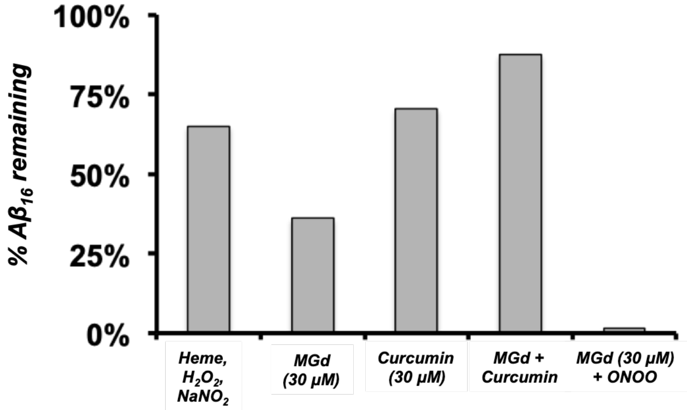

Figure S32. Oxidative and nitrative modification of Aβ16 (300 μM) using hemin (10 μM), H_2O_2 (1 mM), and $NaNO_2$ (1 mM) in 5 mM phosphate buffer at pH 7.4 with and without MGd (30 μM), curcumin (30 μM), or MGd and curcumin. Precise values were calculated for Aβ16 and oxidized products.

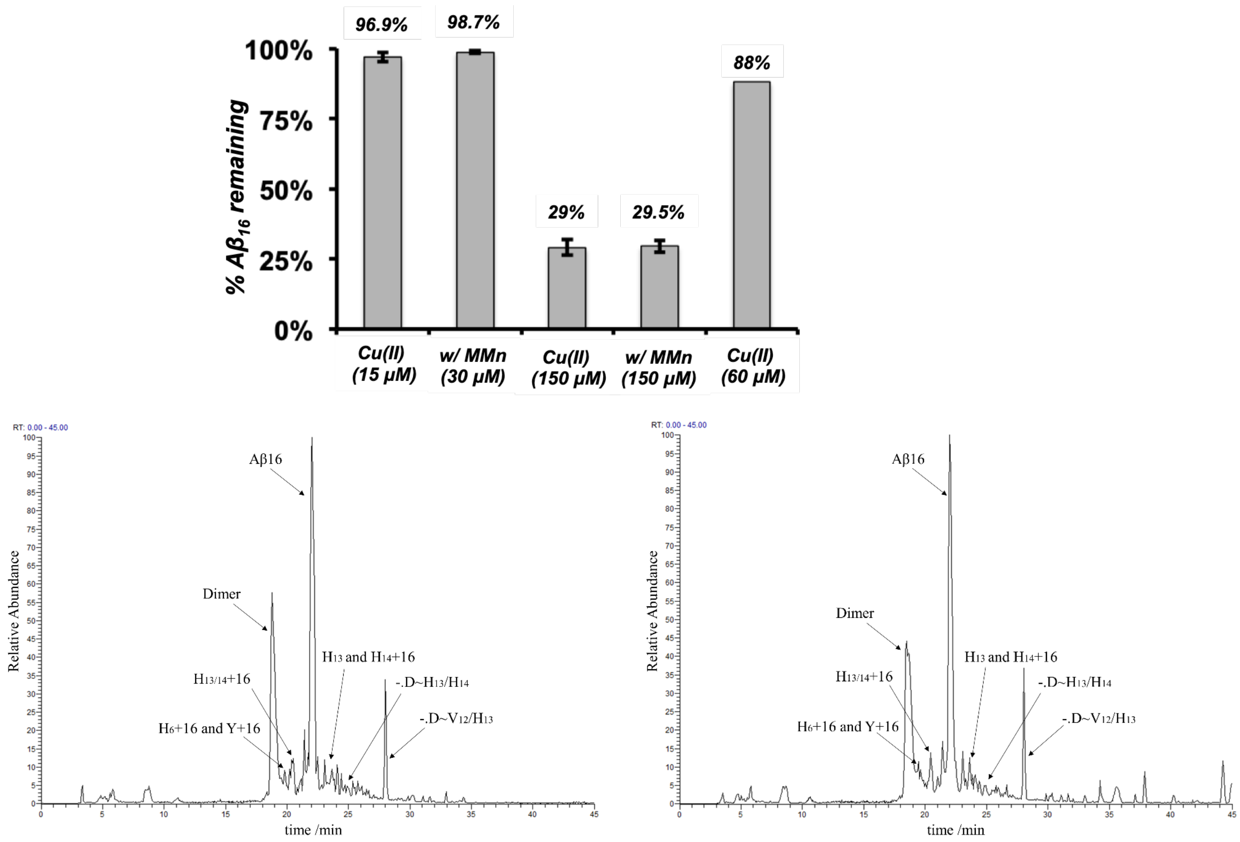

Figure S33. (top) Oxidative modification of Aβ16 (300 μM) using Cu(II) and H_2O_2 (1 mM) in 5 mM phosphate buffer at pH 7.4 with and without MMn (30 μM). Precise values with standard deviations were calculated for Aβ16 and Aβ16 containing a nitrated tyrosine residue. (bottom) HPLC traces using Cu(II) (150 μM) with (*left*) and without MMn (150 μM) (*right*).

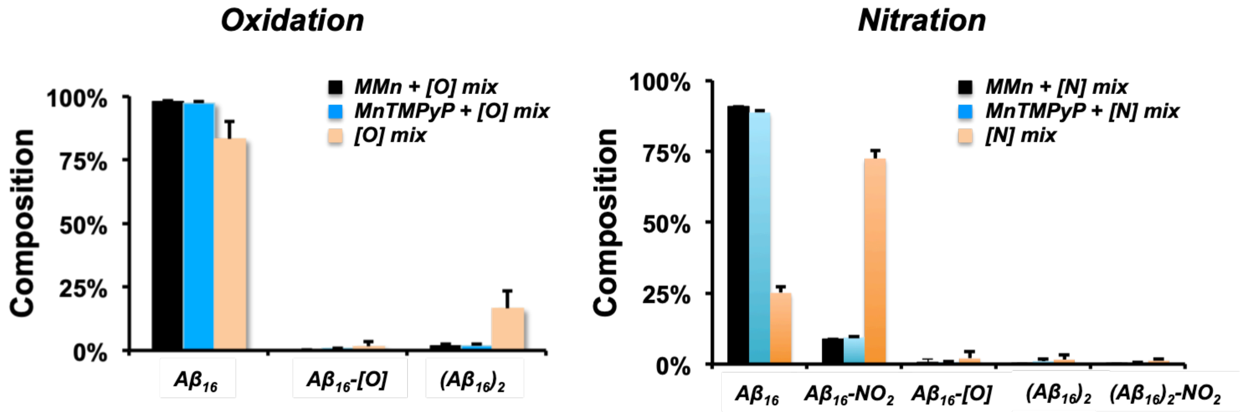

Figure S34. Oxidative and nitrative modification of Aβ16 (300 μM) using hemin (15 μM), H_2O_2 (1 mM), and $NaNO_2$ (1 mM) in 5 mM phosphate buffer at pH 7.4 with and without MMn (30 μM) or MnTMPyP (30 μM). Precise values were calculated for the shown species (*x-axis*). Each experiment is an average of two independent runs.

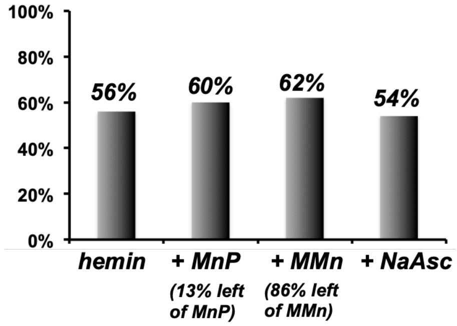

Figure S35. Time dependent percentages for the oxidative decomposition of hemin (15 μM) with and without MnTMPyP (denoted MnP; 5 μM), MMn (5 μM), and sodium ascorbate (40 μM) in PBS (pH 7.15) incubated at 37 °C for 10 minutes.

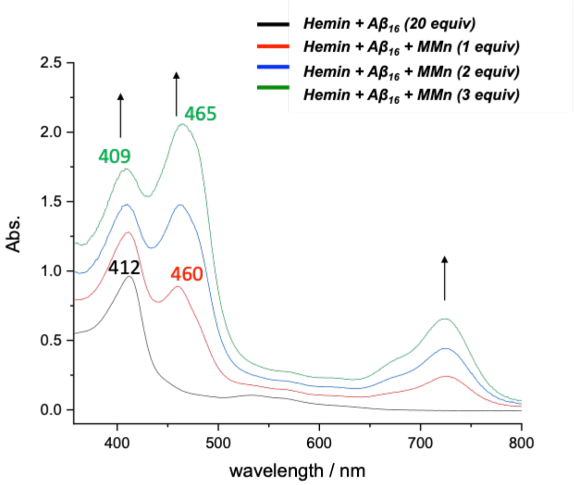

Figure S36. UV-vis spectra (300 K, PBS) showing the effect of MMn (1, 2, and 3 equiv.) on the spectral features of hemin-($A\beta_{16}$)$_2$. The 2:1 adduct was formed by adding 200 μM of $A\beta_{16}$ to 10 μM of hemin as inferred from the observation of the λ_{max} = 412 nm.

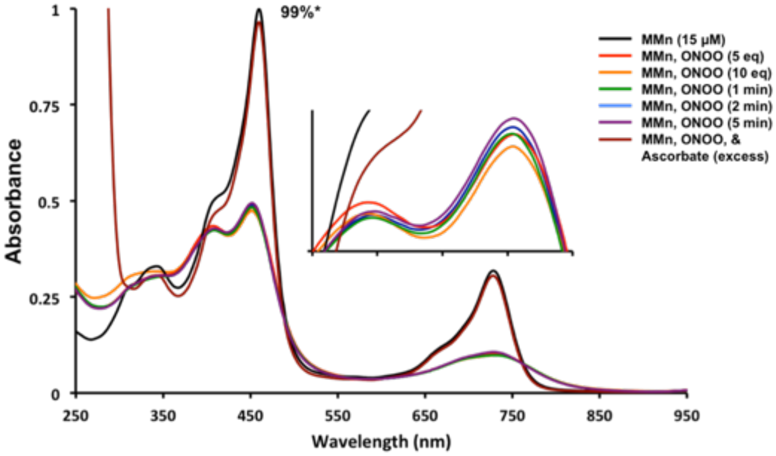

Figure S37. UV-vis spectra of MMn in the presence of peroxynitrite (5 and 10 equiv.) followed by addition of sodium ascorbate (excess) and incubating 5 minutes at room temperature. The insert shows changes to the Mn(**III**) texaphyrin Soret band. **Attributed to dilution*

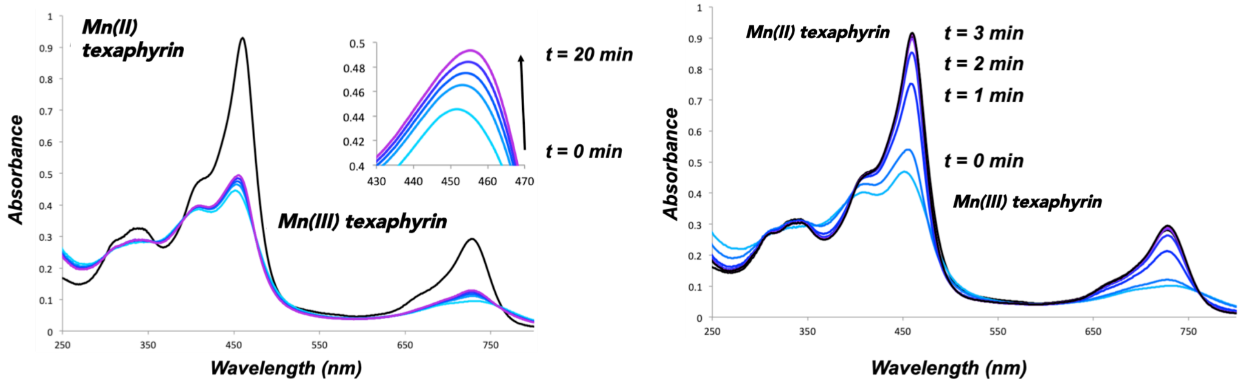

Figure S38. Oxidative disproportionation of H_2O_2 by Mn(III) texaphyrin (15 μM). Mn(III) texaphyrin was prepared by oxidation of Mn(II) texaphyrin with peroxynitrite (150 μM). Auto-reduction in the presence of air going from black (MMn) to light blue to purple (top). Reduction mediated by H_2O_2 going from black (MMn) to light blue to purple (bottom).

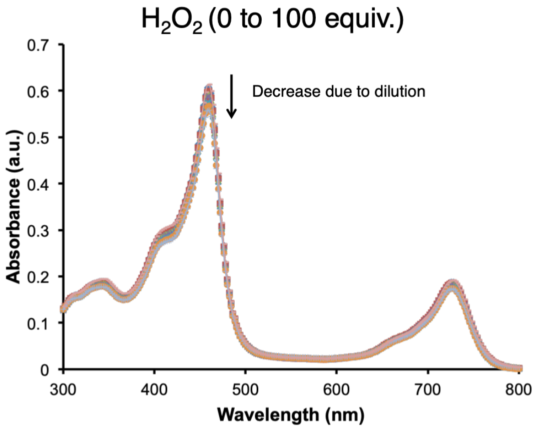

Figure S39. UV-vis spectroscopic titration of Mn(II) texaphyrin (10 μM) with increasing concentrations of H_2O_2 (0 to 100 equiv.).

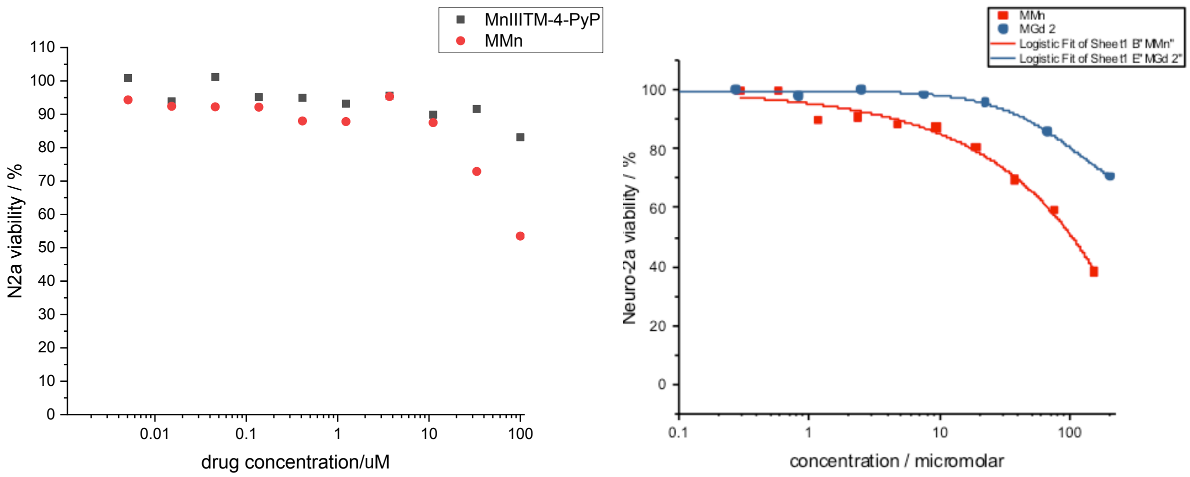

Figure S40. MTT assay measuring the antiproliferative activity against Neuro-2A cells seen after a 24 h exposure time using MMn (red curves, right & left), MnTMPyP (grey square dots, right), and MGd (blue, right).

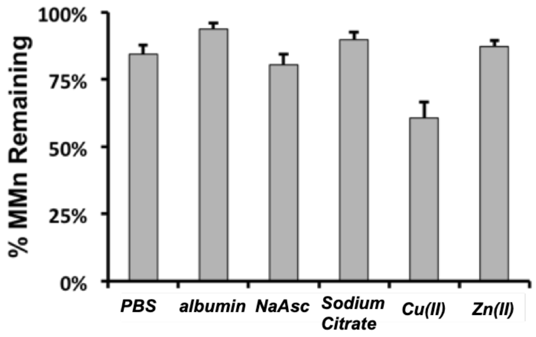

Figure S41. Stability of MMn (5 μM) evaluated by UV-vis spectroscopy after incubating for 24 hours at 37 °C in PBS (5 mM, pH 7.4) while exposed to air: (**1**) in PBS alone, (**2**) with albumin (600 μM), (**3**) with sodium ascorbate (40 μM), (**4**) with sodium citrate (100 μM) as a chelating agent, (**5**) with copper(**II**) (8 μM), and (**6**) with zinc(**II**) (10 μM). Data is an average of three independent runs with error bars showing the standard deviations (SD).

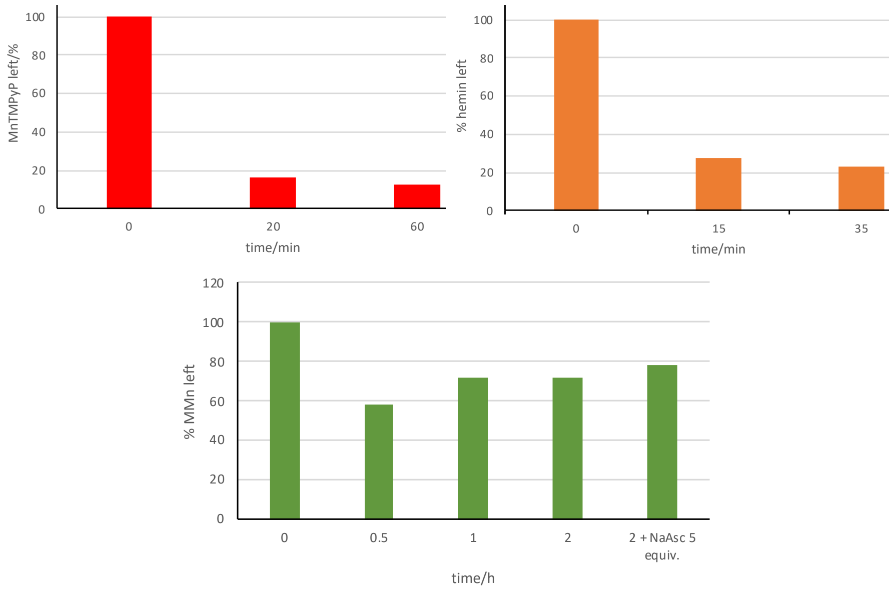

Figure S42. Stability of MnTMPyP (top left), hemin (top right), and MMn (bottom) under oxidative conditions (peroxidase-like activity, [hemin] = 15 □M, [H_2O_2] = 1 mM) with incubation at 37 °C as monitored by means of UV-visible spectroscopy in PBS.

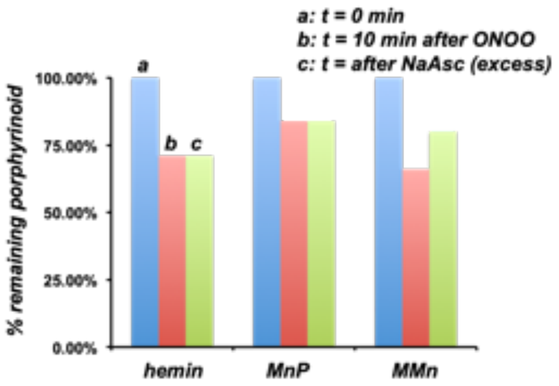

Figure S43. Stability of MnTMPyP (top left), hemin (top right), and MMn (bottom) under oxidative and nitrative conditions using [hemin] = 15 □M, [$ONOO^-$] = 30 □M) with incubation at 37 °C and monitoring by UV-visible spectroscopy in PBS.

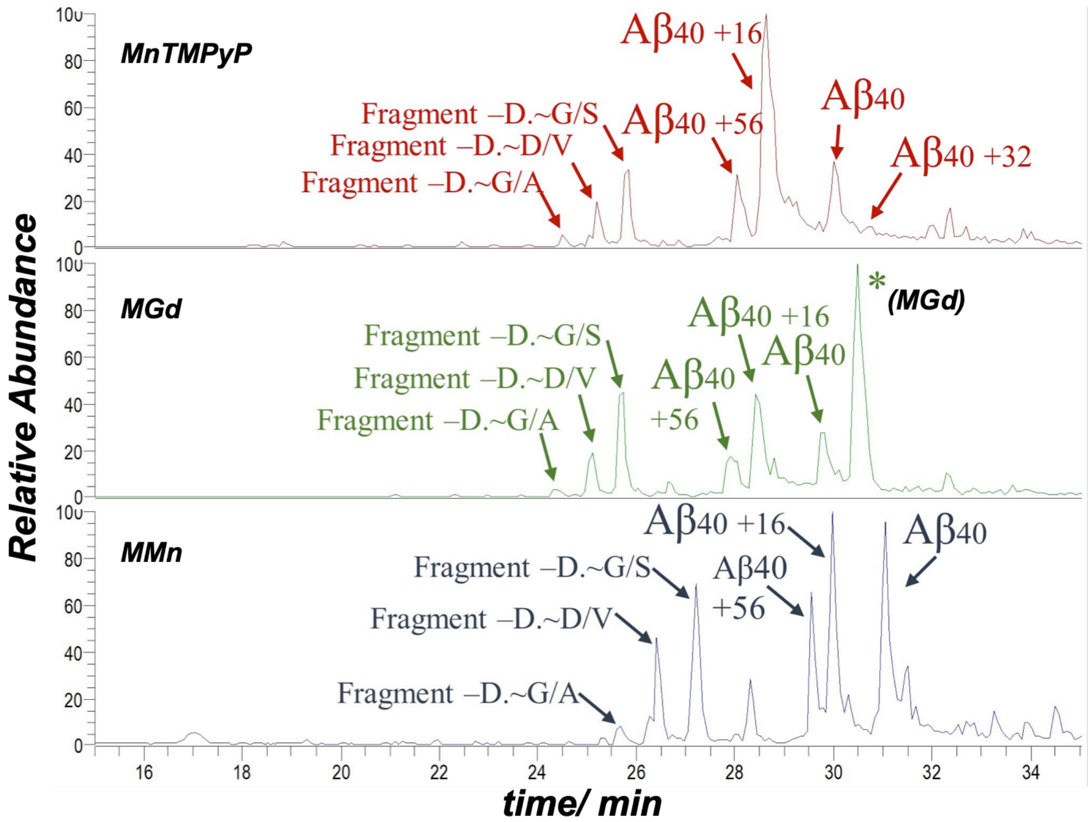

Figure S44. Qualitative HPLC-MS carried out to correlate the level of oxidation of the $A\beta_{40}$ peptide with the cytotoxicity towards N2 cells seen in the MTT assay. MS/MS fragmentation of $A\beta_{40}$+16 and +32 revealed that oxidative modification is predominately comprised of oxidation at His13/His14 and Met35 with a minor contribution from oxidation at Tyr10.

Supplemental Experimental Procedures

General procedures

Starting materials were purchased from Fisher Scientific or Sigma Aldrich and used without further purification unless otherwise specified. Solvents were purified using a solvent purifier system (Vacuum Atmospheres). Dichloromethane (DCM) was freshly distilled after being dried over CaH_2 under argon. Reaction progress was monitored by thin-layer chromatography (TLC silica gel 60 F_{254}, Silicycle UltraPure Silica gels). Analytical RP-HPLC analyses for MMn and MLu were performed on a Thermo scientific Dionex Ultimate 3000 equipped with a PDA detector. The analytical column was a Syncronis C18 column, 5 μm, 4.6 x 250 mm (Thermo Scientific); the mobile phase consisted of a increasing gradient (from 10% to 99 % in 20 min) of acetonitrile into water, both containing 0.1% acetic acid. In this case the flow rate was 1.2 ml / min. Texaphyrins were monitored at 254, 470, and 740 nm. MMn, MGd, and MLu were purified on reverse phase-tC18 SPE (Waters Sep-Pak, Waters) columns containing 10 g of C-18 using an increasing gradient of acetonitrile in either 0.1 M ammonium acetate/ 1% acetic acid aqueous solution or 0.1 M potassium nitrate aqueous solution as the eluent, depending on which counter anion (AcO^- or NO_3^-) was desired as the ancillary ligand. Mass spectrometric analyses were carried out in The University of Texas at Austin Mass Spectrometry Facility. Low-resolution and high-resolution electro- spray mass spectrometric (ESI-MS) analyses were carried out using Thermo Finnigan LTQ and Qq-FTICR (7 Telsa) instruments, respectively. Inductively coupled plasma mass spectrometry (ICP-MS) was carried out at the Bioimaging and Chemical Analysis Facilities Core of the Center for Environmental Health Sciences at MIT using an Agilent 7900 ICP-MS system. In general, samples were digested for 2 hours at 70 °C in

concentrated nitric acid, before being diluted into 2% nitric acid to a working concentration of 10-100 ppb. Nine external standards (0-500 ppb) of the target metal were used to calibrate the instrument and 10 ppb Er was used as an internal standard in each sample. Samples were run in duplicate or triplicate to minimize systematic errors. ^{1}H-NMR spectra were recorded using a Varian 400 and 600 (solvent suppression) MHz as well as a Bruker AVII HD 500 with Prodigy liquid nitrogen cryoprobe (purchased with NIH grant 1-S10-OD021508-01). UV-Vis spectra were recorded using a Varian Cary 5000 spectrophotometer at room temperature. Fluorescence spectra were recorded on a Photon Technology International Fluorescence Master fluorimeter. The source was a 75 W Xenon short arc lamp. A cell length of 10 mm was used in all UV-Vis and fluorescence studies. Phantom MRI data were acquired in a 12 cm outer diameter birdcage transceiver for imaging in a 20 cm bore Bruker 7 T Avance III MRI scanner. Samples at varying concentrations (0 up to 500 μM), as determined by inductively coupled plasma mass spectrometry (ICP-MS) in HEPES (10 mM, pH 7.4) or Dulbecco's PBS (as supplied) were loaded into the wells of a 384-well clear polystyrene plate (Thermo Scientific™ Nunc™), which had been pre-cut in half to optimally fit the coil. Unused wells were filled with buffer. A 2 mm slice was imaged through the samples with the field of view of 5 x 5 cm and the data matrices were 256 x 256 points. Longitudinal (R_1) and transverse (R_2) relaxation measurements were acquired using multi-spin multi-echo (MSME) sequences (flip angle = 180°). R_1 parameters; TE = 12 ms, TR = 100, 200 300, 400, 500, 600, 700 800, 900, 1000, 1200, 1500, 3000, 5000 ms. R_2 parameters; TR = 5000 ms, TE = 12, 24, 36, 48, 60, 72, 84, 96, 108, 120, 132, 144, 156, 168, 280, 192, 204, 216, 228, 240, 252, 264, 276, 288, 300, 312, 324, 336, 348, 360 ms. Custom routines written in Matlab (Mathworks, Natick, MA) were used to reconstruct the images and compute relaxation time constants by fitting image intensity data to exponential decay curves. Relaxivity values (r_1 & r_2) were generated from the gradient of the slope obtained by plotting the calculated relaxation rates against the concentration of the agent in each well. Electron Paramagnetic Resonance (EPR) spectroscopy was carried out using a Bruker EMX Plus spectrometer, X-band (9.5 GHz). Experimental settings were amplitude modulation = 10 G and microwave power = 2 mW. The observed spectra were taken as an average of four scans. Circular dichroism (CD) analyses were carried out using a Jasco J-815 CD spectrometer in The University of Texas at Austin Targeted Therapeutic Drug Discovery & Development Program (Ti3D). The data shown in this report was taken as an average of 3 scans from 260 to 195 nm using a 0.05 nm wavelength step. SEM analyses were prepared by drop casting 20 μM samples onto silica wafers (University Wafers) then gently dried with nitrogen. Images were taken on a FEI Quanta 650 ESEM in The University of Texas at Austin-Texas Materials Institute. There are inherent difficulties in working with $A\beta_{40}$ that are often ignored (e.g., aggregation state, solubility, left over additive to solubilize the peptide, etc.). The methods discussed in the ESI proved reproducible throughout the studies and when carried out by multiple authors.

Peptide synthesis. Aβ peptides were prepared via Fmoc amino solid-phase peptide synthesis using a Liberty Blue microwave peptide synthesizer. Preparative RP-HPLC purification of peptides was performed using an Agilent Zorbax SB-C_{18} Prep HT column 21.2 × 250 mm. Analytical RP-HPLC characterization of peptides was performed using an Agilent Zorbax column 4.6 × 250 mm. An Agilent Technologies 6530 Accurate Mass QT of LC/MS was used for high-resolution mass spectra of purified peptides. Solvents used were HPLC grade.

Synthesis and characterizations of metallotexaphyrins MGd, MLu and MMn. Motexafin gadolinium (MGd) was a gift from Pharmacyclics, Inc., recently acquired by Abbvie. MLu and MMn were synthesized using literature protocols.[S1,S2] The purity of MMn, MLu, and MGd was checked by reverse phase-HPLC before use.

1-Octanol: water partition experiments gave the following data: LogP(MGd) = -0.95; LogP(MMn) = 0.69

Solutions of $A\beta_{40}$ peptide. Approximately 2 mg of $A\beta_{40}$ (as a lyophilized powder) was dissolved in 1 mL aqueous NaOH (10 mM). After vortexing and sonication, the solution was filtered through a 0.22 μm PES syringe filter. PBS (pH = 7.15) was added to obtain a final concentration of 100 μM $A\beta_{40}$. The concentration was checked by UV-vis spectrophotometry using the absorbance at 280 nm and a molar absorptivity of 1490 M^{-1} cm^{-1}. A similar procedure was used for the quantitative reactivity studies with the final dilution being in miliQ water to give a stock solution of approximately 600 μM.

Cell uptake experiments. Neuro-2A cells were grown until confluent in T-75 flasks. MMn was added (100 μM) and cells were incubated for 9 h, upon which cells were washed twice with PBS, incubated with trypsin (1X) for 2 minutes, diluted with supplemented EMEM, counted, and pelleted by centrifugation (3 min at 2000 rpm). After supernatant removal, the pellet was suspended/washed one more time with PBS (5 ml) and the pellet was reformed by centrifugation (3 min at 2000 rpm). The cell pellet was digested in concentrated nitric acid (100 μL) for 15 h at 60°C and diluted with miliQ water to a final concentration of 2% HNO_3 before being analyzed by ICP-MS.

Caenorhabditis elegans. The *C. elegans* strains N2 and CL4176 were provided by CGC (nematodes shipped on 35 mm diameter plates), which is funded by the NIH Office of Research Infrastructure Programs (P40-OD010440). *C. elegans* were grown and maintained as described in "WormBook: The Online Review of *C. elegans* Biology" by Theresa Stiernagle (https://www.ncbi.nlm.nih.gov/books/NBK19649/).

Nematode Growth Medium (NGM). NaCl (3 g), agar (17 g) and peptone (2.5 g) were added to 975 ml of H_2O in a 2 L Erlenmeyer flask, which was then autoclaved for 1 h. All work after this was completed under sterile conditions. The flask was cooled and maintained at 60 °C in a water bath to prevent the solution from solidifying. To this heated solution was added $CaCl_2$ (1 ml of a 1 M solution), cholesterol (1 ml of a solution in ethanol at 5 mg/ mL), $MgSO_4$ (1 ml of 1 M solution), and KPO_4 buffer (25 ml of 1 M solution). The NGM solution was dispensed into 10 mm diameter plates (15 ml/plate). The plates were left at room temperature for 1-2 days to allow excess moisture to evaporate. Plates showing contamination were discarded.

***E. coli* OP50 culture & plate.** Lysogeny broth (LB) was prepared by mixing 21 g LB powder (Sigma Aldrich) in 1 L of Milli Q water. LB agar was prepared by adding 17 g/ L of agar (Fisher Sci.) to the LB mixture. Both the broth and agar were autoclaved at 121 °C for 45 minutes. All work after autoclaving was conducted under sterile conditions. A bead of *E. coli* OP50 (BactoBeads, Fisher Sci.) was streaked on an LB agar plate and grown in a static incubator overnight at 37 °C. From this plate, a single colony was isolated and inoculated in 10 mL of LB. The mixture was placed in a shaking incubator heated to 37 °C overnight. From this NMS plates could be prepared by pipetting 1 mL of the overnight culture onto NMS agar. The plate was swirled to spread the culture and the seeded plates were incubated at room temperature overnight. NMR plates were found to have a lawn of *E. coli* OP50 and were stored inverted at 4 °C, with an up to 3-week shelf life.

MMn uptake experiment. After receiving the *C. elegans* animals, N2 and CL4176, from the CGC (University of Minnesota) shipped on petri dishes, agar matrix was cut in small chunks (0.5 x 0.5 cm) with a razor blade and transferred on Nematode Growth Medium (NGM) agar plates previously seeded with *E. coli* strain OP50 as a food source. NGM plates were then placed at 16 °C in order to increase the population of *C. elegans* before drug exposure and prevent significant plaque formation in the CL4176 AD model. After 7 days, MMn was added to plates (1 ml/ plate of a solution at 100 μM), which were incubated at 25 °C for 48 hours. The animals were then transferred to Eppendorf tubes (previously weighted empty on analytical balance) using 1.5 ml of water. The tubes were put on ice for 5 min and centrifuged (14000 rpm for 1 min) and the supernatant was removed. The plate was washed again with 1.5 mL of water and centrifuged again. The resulting pellets were washed twice with 1 ml of DI water. *C. elegans* pellets were lyophilized over a period of 15 h before being weighed (dry pellet weights obtained by lyophilization were used to normalize the MRI and ICP-MS results).

Preparation of peroxynitrite solution. Three separate solutions of $NaNO_2$ (0.6 M), HCl (0.68 M) with H_2O_2 (0.72 M), and NaOH (3.6 M) were prepared and stored in the freezer at -20 °C for thirty minutes. The solutions were transferred to an ice bath and allowed to sit for thirty minutes. The $NaNO_2$ solution was added to a 100 mL beaker placed in an ice bath and stirred at > 500 rpm. The HCl/ H_2O_2 solution and NaOH solutions are added sequentially with the NaOH coming immediately after the HCl/ H_2O_2 solution. *These solutions must be added in sequence (i.e. one then the other) with as little time between the two additions as possible. Best results were obtained by adding the two solutions with a very small offset.* The mixture turned yellow and was allowed to stir in the ice bath for 5 minutes. MnO_2 was added portion wise

until gas evolution ceased. The mixture was split into aliquots, centrifuged, and the peroxynitrite solution was decanted using a glass pipette. The concentration could be calculated using λ_{max} = 302 nm with ε = 1670 $M^{-1}cm^{-1}$. Best results were obtained by centrifugation in a cold room at 4 °C. [peroxynitrite] = 72-86 mM.

Hemin-Serotonin Procedure. Serotonin (1 mM), H_2O_2 (1 mM), hemin (15 μM), and sodium ascorbate without and with MMn (120 μM) or MnTMPyP (120 μM) were incubated in PBS (pH 7.4, 1.5 mL total volume) at 37 °C for 2 hours in the dark. An analogous experiment without sodium ascorbate using the same concentrations of serotonin, H_2O_2, hemin, and MMn or MnTMPyP. The resulting mixture was passed through a reverse phase-tC18 SPE (Waters Sep-Pak, Waters) column containing 10 g of the C-18 substrate. The column was washed with 25 mL of 5% acetonitrile: H_2O to separate the serotonin and 4,4'-serotonin dimer from paramagnetic species. The resulting solution was concentrated using a rotary evaporator. The mixture was taken up in D_2O (0.7 mL) and analyzed via ^1H NMR spectroscopy. NMR spectroscopic yields were calculated by comparing the relative concentration of serotonin to the 4,4'-serotonin dimer. ***Note***: Additional washing of the column failed to yield additional serotonin or the 4,4'-serotonin dimer.

Cu(II) Procedure. Serotonin (1 mM), H_2O_2 (1 mM), $CuCl_2$ (100 μM) without and with MMn (120 μM) or MnTMPyP (120 μM) were incubated in PBS (pH 7.4, 1.5 mL total volume) at 37 °C for 20 hours in the dark. The resulting mixture was passed through a reverse phase-tC18 SPE (Waters Sep-Pak, Waters) column containing 10 g of the C-18 substrate. The column was washed with 25 mL of 5% acetonitrile: H_2O to separate the serotonin and 4,4'-serotonin dimer from paramagnetic species. The resulting solution was concentrated using a rotary evaporator. The mixture was taken up in D_2O (0.7 mL) and analyzed via ^1H NMR spectroscopy. NMR spectroscopic yields were calculated by comparing the relative concentration of serotonin to the 4,4'-serotonin dimer.

Studies of $A\beta_{16}$ & $A\beta_{40}$-hemin-H_2O_2 with and without $NaNO_2$. Hemin (15 μM), $NaNO_2$ (1 mM), H_2O_2 (1 mM), and $A\beta_{16}$ (300 μM) or $A\beta_{40}$ (100 μM) in PBS (pH 7.4, 5 mM, 200 μL total volume) were incubated together at 37 °C for 2 h, unless otherwise specified. The resulting mixtures were subjected to HPLC-MS analysis. Vials were cooled to 4 °C inside the HPLC-MS to prevent additional reactivity.

Studies of $A\beta_{16}$-Cu(II)-H_2O_2. $CuCl_2$ (15, 60, and 150 μM), H_2O_2 (1 mM), and $A\beta_{16}$ (300 μM) with and without MMn (30 and 150 μM) in PBS (pH 7.4, 5 mM, 200 μL total volume) were incubated together at 37 °C for 2 h. The resulting mixtures were subjected to HPLC-MS analysis. Vials were cooled to 4 °C inside the HPLC-MS to prevent additional reactivity.

Studies of $A\beta_{16}$-hemin-peroxynitrite. Hemin (15 μM), peroxynitrite (1 mM) and $A\beta_{16}$ (300 μM) in PBS (200 μL total volume) were incubated together at 37 °C for 30 min. All compounds were added and then vortexed to provide a homogenous mixture. Immediately after adding peroxynitrite, the mixture was vortexed again and allowed to incubate at 37 °C for 30 minutes. The resulting mixtures were immediately subjected to HPLC-MS analysis. Vials were cooled to 4 °C inside the HPLC-MS queue to prevent additional reactivity.

HPLC-MS/MS analyses. Analytical RP-HPLC characterization of peptides was performed using an Agilent Zorbax column 4.6 × 250 mm. An Agilent Technologies 6530 Accurate Mass QT of LC/MS was used for high-resolution mass spectra of purified peptides. Solvents used were HPLC grade. UV-vis spectra were recorded on a Hewlett Packard HP 8453 A diode array spectrophotometer equipped with a thermostated, magnetically stirred cuvette holder. The peptides were studied by direct HPLC-MS/MS analysis. LC-MS and LC-MS/MS data were obtained by using an LCQ DECA ion-trap mass spectrometer with an ESI ion source, coupled with an automatic injector Surveyor HPLC system and controlled by Xcalibur 1.3 software (Thermo-Finnigan, San Jose, CA). The system was run in an automated LC-MS/MS mode and by using a Surveyor HPLC system (Thermo-Finnigan, San Jose, CA, USA) equipped with a BIOBASIC C18 column (5 μM), 150 x 2.1 mm). For analysis of oxidation studies, the acquired MS/MS spectra were automatically searched against the human $A\beta_{16}$ sequence by using the SEQUEST algorithm

to identify the modified residues. This algorithm was incorporated into the Bioworks 3.1 software (Thermo-Finnigan, San Jose, CA). The elution was performed with a 0-55% linear gradient over 65 min with 0.1% formic acid in water as solvent A and 0.1% formic acid in acetonitrile as solvent B with a flow rate of 0.2 mL/min. For analysis of oxidation studies, the acquired MS/MS spectra were automatically searched against the human $A\beta_{16}$ sequence by using the SEQUEST algorithm to identify the modified residues. This algorithm was incorporated into the Bioworks 3.1 software (Thermo-Finnigan, San Jose, CA).

MS/MS spectrometric analyses of the $A\beta_{16}$ ion series samples provided support for the conclusion that nitration occurs to Tyr10. MS/MS fragmentation of $A\beta_{16}$ revealed that His13 or His14 are the major oxidized species with dimerization occurring at Tyr10. MS/MS fragmentation of $A\beta_{40}$ revealed that oxidative modification is predominately comprised of oxidation at His13/His14 and Met35 with Tyr10 oxidation being minimal.

Cell culture. The neuroblastoma cell line Neuro-2A was bought from ATCC. Cells were grown in EMEM medium containing 10% heat inactivated fetal bovine serum (FBS) and antibiotics (100 mg/ ml streptomycin and 100 U/ ml penicillin) at 37 °C, under 5% CO_2 atmosphere. The cells were sub-cultured once every 4 to 5 days.

MTT viability assay (determination of IC_{50} values for MMn, MnTMPyP, and MGd). The proliferation of exponential phase cultures of Neuro-2A was assessed by tetrazolium (MTT) dye reduction. In brief, tumor cells were seeded in 96-well microliter plates at 10,000 cells per well and allowed to adhere overnight in the culture medium described previously. After 15 h, agents were added via serial dilution (from 200 to 0.01 μM) and the plate was incubated at 37°C and 5% CO_2. After 24 h incubation time with the agent in question (2 days overall) 50 μL of a solution of tetrazolium dye in EMEM without FBS (3 mg/ mL) was added to each well. The plate was incubated for 4 hours, whereupon the medium was removed and the formazan product dissolved in 50 μL of DMSO. The absorbance at 570 nm was measured using a microplate reader (Molecular Devices, Sunnyvale, CA), corrected for background and then normalized to wells containing untreated cells to allow plate-to-plate comparisons. The growth inhibition data were fitted to a sigmoidal dose-response curve (logistic function, origin software) to generate IC_{50} values (defined as the concentrations of drug inhibiting cell growth by 50%). The IC_{50} is presented as mean +/- standard deviation.

24-well plate MTT experiments. $A\beta_{40}$ (90 μM) was incubated in PBS alone or in the presence of H_2O_2 (1 mM), hemin (15 μM), $NaNO_2$ (1 mM) (referred to as nitration mixture) for 3 h at 37 °C followed by 24 h at 4 °C. 250 μL of each of the solution was transferred into each well containing about 50,000 Neuro-2A cells (plated 24 h earlier) in 250 μL of supplemented EMEM medium without phenol red. After 24 h, the medium was removed and replaced with 500 μL of fresh EMEM not containing FBS and phenol red. To this was added, 200 μl of an MTT solution at 3 mg/ml in EMEM not containing phenol red and FBS. After 4 h at 37 °C, the supernatant was removed and 200 μl of DMSO was added to each well to dissolve the formazan purple crystals. Aliquot (50 μl) of each well were transferred into a 96-well plate well and the absorbance at 570 nm was read using a plate reader (see description above).

References

[S1] Sessler, J. L., Mody, T. D., Hemmi, G. W., and Lynch, V. (1993) Synthesis and structural characterization of lanthanide(III) texaphyrins. *Inorg. Chem. 32*, 3175-3187.

[S2] Hannah, S., Lynch, V.M., Guldi, D. M., Gerasimchuk, N., MacDonald, C. L. B., Magda, D., and Sessler, J. L. (2002) Late First-Row Transition-Metal Complexes of Texaphyrin. *J. Am. Chem. Soc. 124*, 8416-8427.
